# Deep learning imputes DNA methylation states in single cells and enhances the detection of epigenetic alterations in schizophrenia

**DOI:** 10.1016/j.xgen.2025.100774

**Published:** 2025-02-21

**Authors:** Jiyun Zhou, Chongyuan Luo, Hanqing Liu, Matthew G. Heffel, Richard E. Straub, Joel E. Kleinman, Thomas M. Hyde, Joseph R. Ecker, Daniel R. Weinberger, Shizhong Han

**Affiliations:** 1 Lieber Institute for Brain Development, Johns Hopkins Medical Campus, Baltimore, MD 21287, USA; 2 Department of Human Genetics, University of California, Los Angeles, Los Angeles, CA 90095, USA; 3 Genomic Analysis Laboratory, The Salk Institute for Biological Studies, La Jolla, CA 92037, USA; 4 Bioinformatics Interdepartmental Program, University of California, Los Angeles, Los Angeles, CA 90095, USA; 5 Howard Hughes Medical Institute, The Salk Institute for Biological Studies, La Jolla, CA 92037, USA; 6 Department of Psychiatry and Behavioral Sciences, Johns Hopkins University School of Medicine, Baltimore, MD 21287, USA; 7 Department of Genetic Medicine, Johns Hopkins University School of Medicine, Baltimore, MD 21205, USA; 8 Department of Neuroscience, Johns Hopkins University School of Medicine, Baltimore, MD 21205, USA; 9 Department of Neurology, Johns Hopkins University School of Medicine, Baltimore, MD 21205, USA

**Keywords:** DNA methylation, deep learning, transformer, imputation, schizophrenia

## Abstract

DNA methylation (DNAm) is a key epigenetic mark with essential roles in gene regulation, mammalian development, and human diseases. Single-cell technologies enable profiling DNAm at cytosines in individual cells, but they often suffer from low coverage for CpG sites. We introduce scMeFormer, a transformer-based deep learning model for imputing DNAm states at each CpG site in single cells. Comprehensive evaluations across five single-nucleus DNAm datasets from human and mouse demonstrate scMeFormer’s superior performance over alternative models, achieving high-fidelity imputation even with coverage reduced to 10% of original CpG sites. Applying scMeFormer to a single-nucleus DNAm dataset from the prefrontal cortex of patients with schizophrenia and controls identified thousands of schizophrenia-associated differentially methylated regions that would have remained undetectable without imputation and added granularity to our understanding of epigenetic alterations in schizophrenia. We anticipate that scMeFormer will be a valuable tool for advancing single-cell DNAm studies.

## Highlights

• scMeFormer is a deep learning model for single-cell DNA methylation data imputation
• Outperforms state-of-the-art methods in accuracy and efficiency
• Includes a filtering metric for quality control of imputed data
• Identifies thousands of schizophrenia-associated DMRs undetectable without imputation

## Graphical abstract

**Figure:**
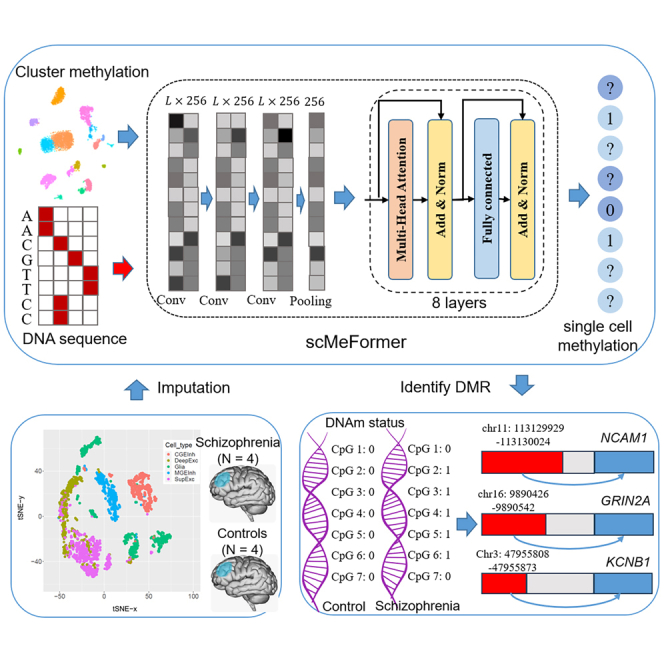

## Teaser

Zhou et al. developed scMeFormer, a deep learning model for imputing single-cell DNA methylation datasets. It outperforms existing methods in accuracy and efficiency. Applying scMeFormer to single-nucleus DNAm data from the prefrontal cortex of patients with schizophrenia and controls uncovered thousands of schizophrenia-associated differentially methylated regions that were undetectable without imputation.

## Introduction

DNA methylation (DNAm) is a fundamental epigenetic mechanism that involves the addition of a methyl group to cytosines and plays a crucial role in gene regulation, mammalian development, and various human diseases.^1^ Single-cell technologies enable the profiling of DNAm states at cytosines within the DNA sequence of individual cells, which complements single-cell transcriptome studies in understanding cellular heterogeneity, developmental processes, and disease states.^2^^,^^3^ However, due to the limited DNA material available from individual cells and inherent technical limitations, current technologies often suffer from sparse coverage for CpG sites, typically measuring less than 10% of CpG sites in a single cell.^4^^,^^5^^,^^6^^,^^7^ This limits their full potential to uncover the epigenetic landscape at single-cell resolution.

In order to address the challenge of sparse coverage in the single-cell DNAm dataset, computational methods have been developed for imputing DNAm states for CpG sites in individual cells. One such method is Melissa, a Bayesian model that draws inferences solely from DNAm profiles,^8^ but it was designed for genomic regions of interest rather than genome-wide imputation. LightCpG is a traditional machine learning model that uses a light gradient boosting machine (LightGBM) to build prediction models with handcrafted features derived from CpG sites and DNA sequences to impute missing methylation states.^9^ Similarly, CaMelia employs a CatBoost gradient boosting algorithm to predict methylation states based on the local similarity of methylation patterns across cells.^10^ EpiScanpy,^11^ an extension of the Scanpy toolkit^12^ tailored for single-cell epigenomic data analysis, imputes DNAm levels in genomic intervals by replacing missing values with the average methylation across all cells for those intervals.

In recent years, deep-learning-based models have emerged for imputing single-cell DNAm data, automatically extracting features from DNAm profiles and DNA sequences. The first such model, DeepCpG,^13^ is based on a traditional recurrent neural network. While DeepCpG learns features from both DNAm profiles and DNA sequences, it is limited by its ability to capture long-range dependencies and is computationally intensive. Another deep learning model, CpG Transformer,^14^ builds upon the transformer model initially designed for language processing, which has the key advantage of capturing long-range dependency through an attention mechanism. CpG Transformer demonstrated superior performance compared to DeepCpG, but both models exhibit limitations in scalability that can only process up to several hundred cells, making them impractical for datasets involving thousands of cells—a scenario increasingly prevalent in the field.^4^^,^^5^^,^^6^^,^^7^ The most recent model, GraphCpG,^15^ employed a graph-based deep learning design and achieved state-of-the-art performance. However, GraphCpG only utilized the DNAm profile and did not consider DNA sequence features. Moreover, due to the single prediction task design of GraphCpG, this model can predict only one CpG in one cell at a time, making it challenging to scale to thousands of cells.

Here, we introduce scMeFormer, a transformer-based deep learning model, to efficiently impute DNAm states for each CpG site across thousands of single cells within a multi-task prediction framework, leveraging information from both local DNA sequences and DNAm profiles across cells. We demonstrate the superior performance of scMeFormer compared to alternative models across five single-nucleus DNAm datasets from the human brain and mouse embryo,^4^^,^^5^^,^^6^^,^^7^^,^^16^ achieving high-fidelity imputation even with coverage reduced to 10% of original CpG sites. We further applied scMeFormer to a single-nucleus DNAm dataset we generated from the prefrontal cortex of four patients with schizophrenia (SCZ) and neurotypical controls. This enabled the identification of thousands of differentially methylated regions (DMRs) associated with SCZ that would have remained undetectable without imputation and added granularity to our understanding of epigenetic alterations in SCZ within specific cell types.

## Results

### scMeFormer predicts DNAm states of CpG sites in single cells

**Figure 1:**
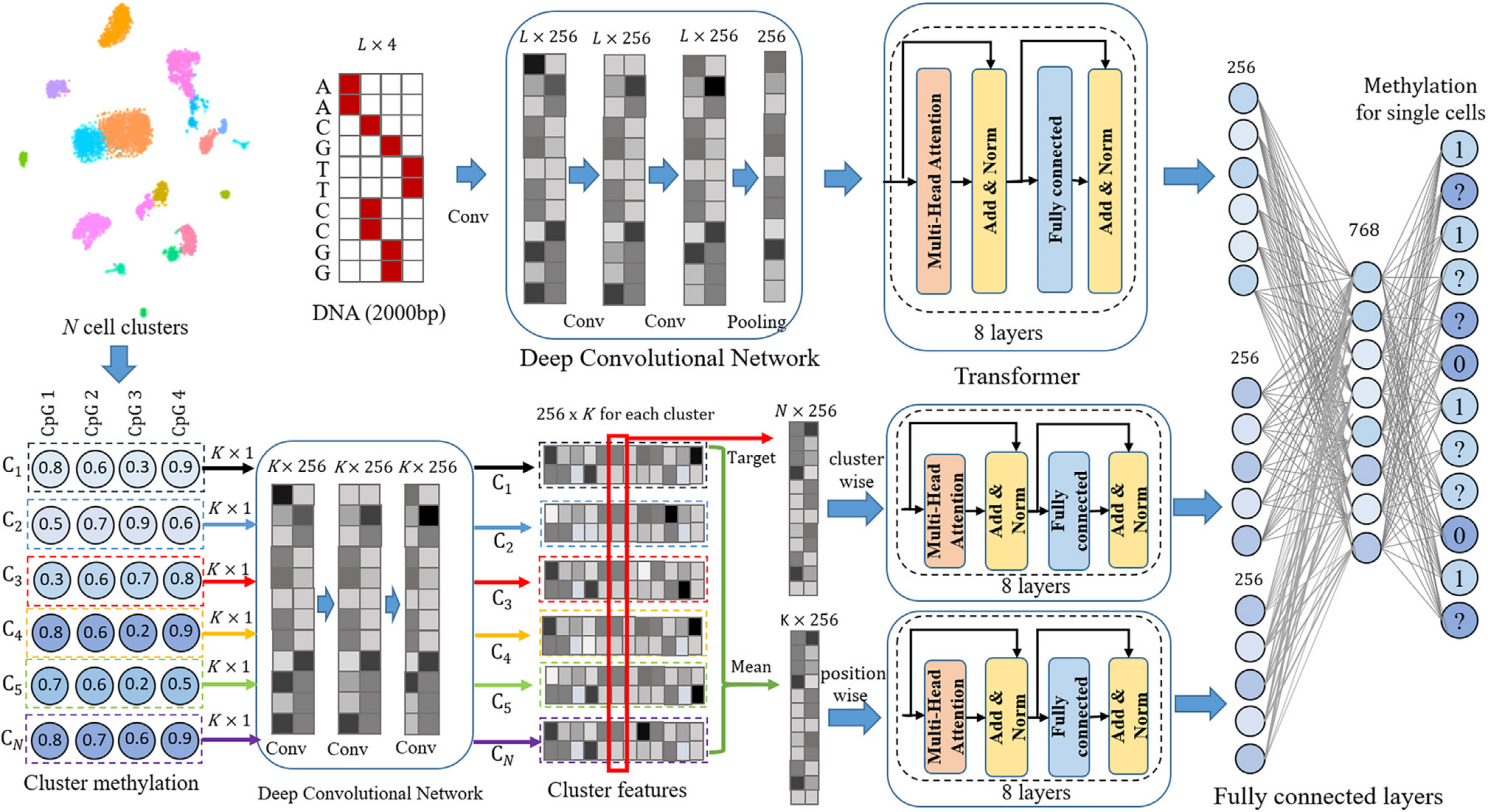
Illustration of scMeFormer architecture scMeFormer consists of two main modules: the DNA module and the CpG module. The DNA module includes a three-layer CNN followed by an eight-layer transformer. It takes DNA sequences of dimension L × 4 (where L is the sequence length, equal to 2,000 bp in the real model) as input. The CNN extracts local features from the DNA sequences, which are then passed to the transformer to learn potential interactions among local DNA features. Similarly, the CpG module comprises a three-layer CNN and two eight-layer transformers. The input to the CNN is the DNAm levels of K CpG sites (K set to 100 in the real model) within each pre-defined cell cluster (C_1_, C_2_, …, C_N_). The CNN identifies local DNAm patterns of CpG sites within each cluster through a one-dimensional convolutional kernel (256 kernels), generating a feature matrix of dimensions 256 × K for each cluster. The mean feature matrix across clusters is then fed into a position-wise transformer to capture relationships among positions. The feature matrix (red box) at the position of the target CpG is passed to the cluster-wise transformer to capture DNAm relationships among clusters. The output feature vectors from the DNA and CpG modules are catenated and passed into a fully connected network for prediction.

The scMeFormer model architecture comprises two main modules: a DNA module and a CpG module (Figure 1). The DNA module is designed to learn DNAm motifs, while the CpG module aims to leverage DNAm information from neighboring CpG sites within and across pre-defined cell clusters. The DNA module contains a convolutional neural network (CNN) block for extracting local DNA features, followed by eight layers of transformer blocks to detect distant features that may cooperate to influence DNAm. The CpG module consists of a CNN block for extracting local patterns from neighboring CpG sites within each cell cluster, followed by two separate transformers to capture DNAm relationships among CpG sites and cell clusters. The features learned from both modules are catenated within a fully connected network to predict DNAm states for a given CpG site in a subset of cells whose DNAm states are measured. The model is trained by minimizing the prediction error, with only measured CpG sites contributing to the loss function in each training step.

**Figure 2:**
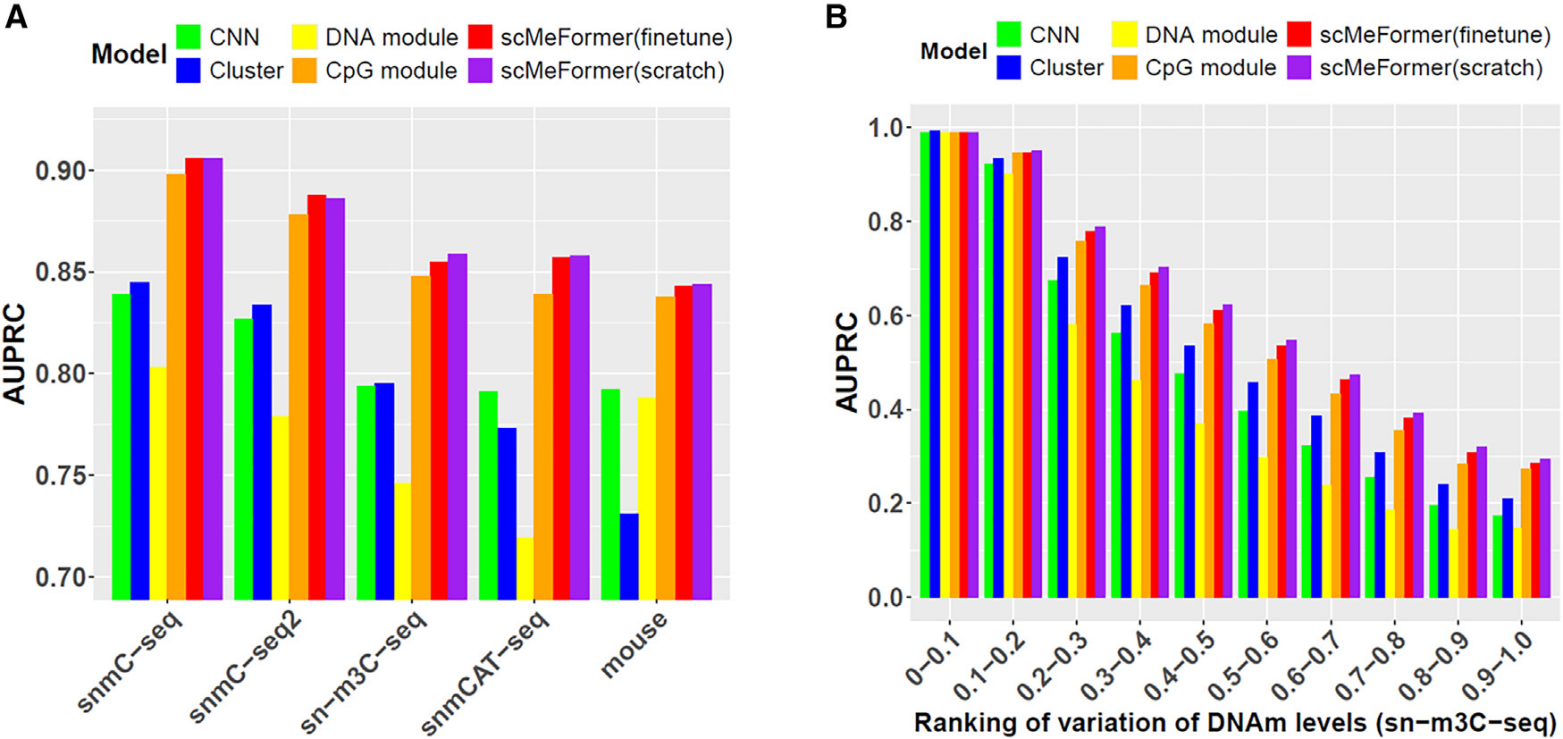
Comparison of model prediction performance between scMeFormer and four alternative models across five single-nucleus DNAm datasets (A) Comparison was based on all independent testing CpG sites on chromosome 22. (B) Comparison was based on subsets of independent testing CpG sites, stratified by their levels of variations across all cells in the sn-m3C-seq dataset. The “0–0.1” group represents the bottom 10% least-variable CpG sites. The “0.9–1.0” group represents the top 10% most-variable CpG sites. See also Figure S1 and Table S2.

We applied scMeFormer to five distinct single-nucleus DNAm datasets: four human brain datasets generated with unique technologies (snmC-seq,^4^ snmC-seq2,^6^ sn-m3C-seq,^5^ and snmCAT-seq^7^) and one mouse embryo dataset generated by scNMT-seq.^16^ These datasets ranged in size from 1,105 (scNMT-seq) to 4,357 (snmCAT-seq) nuclei. Model prediction performance was evaluated on each dataset using independent CpG sites not included in either model training or validation. When trained from scratch on each dataset (scMeFormer(scratch)), scMeFormer achieved remarkable prediction performance, with an average area under the precision-recall curve (AUPRC) of 0.871 across five datasets (Figure 2A; Table S1). To improve computational efficiency, we also implemented a pre-training and fine-tuning strategy, in which scMeFormer was initially pre-trained on one dataset and then fine-tuned on independent datasets. Remarkably, the fine-tuned model (scMeFormer(fine-tune)) performed comparably to the model trained from scratch across datasets, achieving an average AUPRC of 0.869. This strategy enables scMeFormer to be pre-trained once and then adapted to new datasets without retraining from scratch, significantly reducing computational costs. Notably, this strategy proved effective even across species: we pre-trained the model on the human sn-m3C-seq dataset and fine-tuned it on the mouse embryo dataset, achieving nearly the same performance as the model trained directly on the mouse dataset from scratch (fine-tune: AUPRC = 0.843; scratch: AUPRC = 0.844).

We further explored the contribution of each module to the overall performance of scMeFormer. A reduction in prediction performance occurred when scMeFormer employed only the CpG module (average AUPRC = 0.860) or the DNA module (average AUPRC = 0.767), indicating that both modules contribute complementary information for predicting DNAm states. Additionally, we compared scMeFormer with two alternative models: a CNN model without transformer blocks and a cluster-based model that imputes CpG states based on the mean DNAm levels of CpG sites in cells of the same cluster. scMeFormer consistently outperformed the two alternative models across the five datasets (Figure 2A).

We further evaluated the prediction performance of various models on subsets of independent CpG sites, stratified by their levels of variations across cells (Figures 2B and S1; Table S2). All models performed well for CpG sites with the least variability. For example, for CpGs ranked in the bottom 10% in terms of their variations, all models achieved an AUPRC > 0.97 across the five datasets. Notably, we observed a reduction in prediction performance for more variable CpG sites across all models and datasets. Nonetheless, scMeFormer consistently outperformed alternative models. For example, within the subset of the most variable CpG sites (ranked in the top 10%), scMeFormer(fine-tune) achieved an average AUPRC of 0.219 across the five datasets, which was 0.083 and 0.039 higher than the CNN and cluster models, respectively. For CpG sites with intermediate variability (ranked between the top 40% and top 50%), scMeFormer achieved an average AUPRC of 0.459, which was 0.144 higher than the CNN and 0.092 higher than the cluster model across the datasets.

To assess the genomic regions where imputation errors are more likely to occur, we evaluated the model prediction performance on CpG sites stratified by their chromatin states defined in the dorsolateral prefrontal cortex derived from the ROADMAP Epigenomics Project (Figure S2; Table S3). We observed that scMeFormer performed well (AURPC > 0.8) for CpG sites within five active states (TssA, TssAFlnk, TxFlnk, Enh, and EnhG), three poised states (TssBiv, BivFlnk, and EnhBiv), and the repressed Polycomb state (ReprPC). However, its performance was relatively lower for CpG sites in actively transcribed states (Tx and TxWk), regions associated with zinc-finger protein genes (ZNF/Rpts), heterochromatin (Het), weakly repressed Polycomb states (ReprPCWk), and quiescent regions (Quies).

We compared scMeFormer with three previous deep learning models (GraphCpG, CpG Transformer, and DeepCpG) and CaMelia, a gradient-boosting-based model. Due to the limitation of scalability of these models, we trained them on a randomly selected subset of 118 cells from the sn-m3C-seq dataset. Overall, scMeFormer achieved the highest performance (AUPRC = 0.869), followed by CpG Transformer (AUPRC = 0.868)*,* DeepCpG (AUPRC = 0.816), CaMelia (AUPRC = 0.809), and GraphCpG (AUPRC = 0.735). Moreover, scMeFormer consistently outperformed these four models (except for CpG Transformer) when evaluated on subsets of CpG sites stratified by their levels of variation across cells (Figure S3).

### scMeFormer performance under lower CpG coverage through downsampling

**Figure 3:**
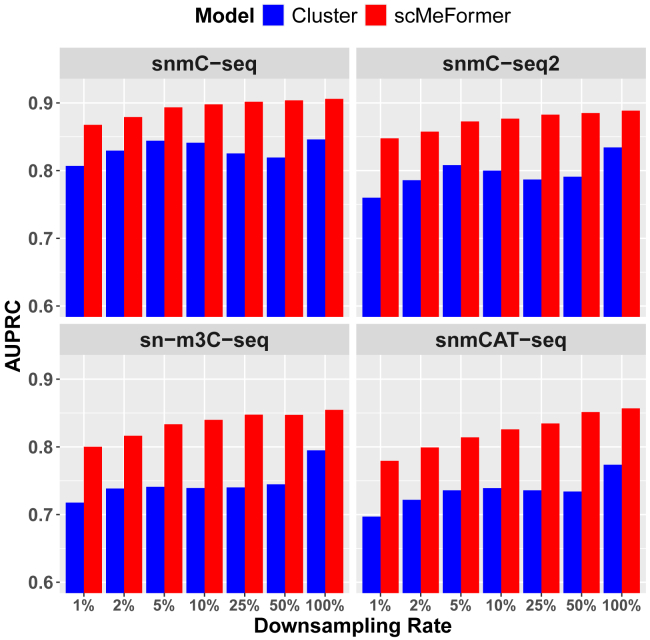
Comparison of model prediction performance between scMeFormer and the cluster model across the four human datasets under reduced CpG coverage through downsampling Comparison was based on independent testing CpG sites on chromosome 22 in each dataset. As a comparison, we included prediction performance from raw data without downsampling as indicated by downsampling rate = 1 on the x axis. See also Table S4.

We assessed the performance of scMeFormer under conditions of lower CpG coverage in single cells through downsampling. Due to the significant reduction in training time, all of the following results from scMeFormer refer to fine-tuned models unless otherwise specified. We systemically reduced the number of CpG sites used for training the model from 50% to 1% to mimic scenarios with lower coverage. In this investigation, we compared scMeFormer with only the cluster model since the cluster model performed generally better than the CNN in datasets without downsampling. We observed a trend of slightly reduced prediction performance at lower CpG coverage for both models, but scMeFormer consistently outperformed the clustering model across all five datasets (Table S4). Figure 3 illustrates the comparative results across the four human datasets. Notably, both models achieved good performance even at 1% of the original CpG coverage (scMeFormer, average AUPRC = 0.821; cluster, average AUPRC = 0.728). Additionally, we evaluated the two models under downsampling situations for subsets of independent CpG sites, stratified by their levels of variations across cells (Figure S4; Table S5). Consistent with our observations in non-downsampled datasets, a reduction in prediction performance occurred for more variable CpG sites for both models. However, scMeFormer demonstrated superior performance compared to the cluster model across all five datasets, particularly for the more variable CpG sites.

### An imputation quality metric enhances imputation performance

**Figure 4:**
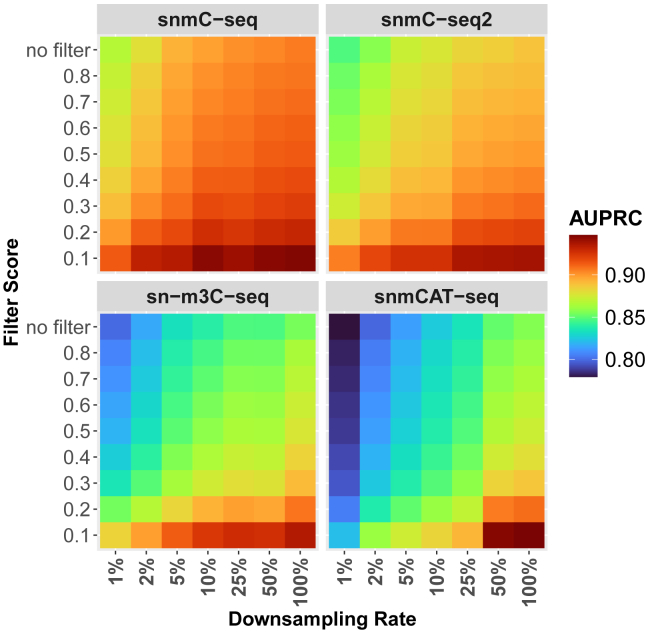
Comparison of scMeFormer model prediction performance at different filtering score thresholds across four human datasets, evaluated under various levels of reduced CpG coverage achieved through downsampling The x axis represents various downsampling rates, and the y axis represents the applied filtering score thresholds, including a no-filtering condition. Comparison was based on independent testing CpG sites on chromosome 22 in each dataset. See also Table S6.

To evaluate the quality of imputed DNAm states for each CpG site in each cell, we defined an imputation quality score metric, which measures the average absolute difference of predicted DNAm states between the target CpG site and its five upstream and five downstream neighboring CpG sites. We reasoned that lower score values indicate more reliable imputed states for the target CpG sites, as methylation states tends to be similar among nearby CpG sites. In contrast, higher score values may reflect poorer imputations that resemble random guesses. We applied various score thresholds as filters, retaining only CpG sites with scores below the threshold. Remarkably, we observed a clear trend of improved prediction performance with more stringent filtering (Figure 4; Table S6), supporting the reliability of this filtering metric. For instance, in the sn-m3C-seq dataset without downsampling, the AUPRC increased from 0.855 without filtering to 0.935 when a filtering score threshold of 0.1 was applied. This trend was consistently observed across datasets downsampled at different rates.

### scMeFormer recovers cell clusters under low coverage through downsampling

**Figure 5:**
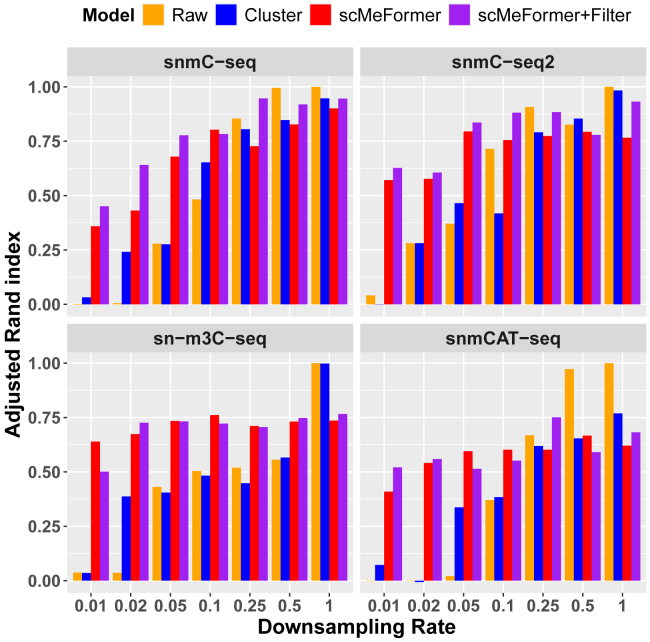
Evaluation of imputed data under lower CpG coverage in its ability to recover cell types identified in the original data across four human datasets The x axis represents various downsampling rates, and the y axis represents the adjusted Rand index. The orange bar (raw) represents the downsampled data without imputation. See also Table S7.

To further assess the fidelity of imputed data under reduced CpG coverage achieved by downsampling, we examined how well imputed CpG sites could recover cell type clusters originally identified from the raw data across the four human datasets. We used the adjusted Rand index (ARI) to measure the similarity between clusters obtained from the original data and those derived from (1) downsampled data without imputation, (2) downsampled data imputed by the cluster model, and (3) downsampled data imputed by scMeFormer without or with a filtering (score = 0.2). Overall, scMeFormer with filtering achieved superior performance (average ARI = 0.72) compared to scMeFormer without filtering (average ARI = 0.67) across all datasets and downsampling rates (Table S7). scMeFormer maintained robust performance (ARI > 0.6) across all four datasets, even at a downsampling rate as low as 0.05, but this was not the case for the cluster model or the method without imputation (Figure 5; Table S7). Specifically, scMeFormer achieved average ARIs of 0.70 (without filtering) and 0.71 (with filtering), whereas the other two methods yielded much lower ARIs (without imputation, ARI = 0.28; cluster model, ARI = 0.37). At even lower downsampling rates (<0.05), the performance of the other two methods substantially declined, whereas scMeFormer exhibited a slower rate of decay, remaining comparable to its performance at a downsampling rate of 0.05.

### scMeFormer boosts the detection of cell-type-specific DMRs

To further evaluate the quality of imputed data, we assessed its ability to recover DMRs between cell types identified in the original data for two datasets (sn-m3C-seq and snmCAT-seq). We defined cell-type-specific DMRs as regions containing at least two differentially methylated CpG sites (DMSs), with each DMS exhibiting lower DNAm levels in the specific cell type, since hypo-DMRs are strong indicators of regulatory elements.^17^^,^^18^^,^^19^ We applied three filtering scores (0.2, 0.3, and 0.4) on the imputed datasets. As expected, we observed a trend of higher recall rates with less stringent filtering in both the raw and the 10% downsampled data (Figure S5; Table S8). For instance, with a filtering score of 0.2, scMeFormer achieved average recall rates of 0.45 in the sn-m3C-seq dataset and 0.47 in the snmCAT-seq dataset for cell-type-specific DMRs across all cell type pairs. When the filtering score increased to 0.4, the recall rates rose to 0.69 in both datasets. Notably, even on 10% downsampled data, scMeFormer maintained slightly lower yet comparable recall rates across both datasets. These results demonstrate the efficacy of scMeFormer in imputing CpG sites that preserve information crucial for identifying cell-type-specific DMRs.

We compared the number of cell-type-specific DMRs detected from imputed data with that from raw data. In the raw, unimputed datasets, we identified an average of only 304 DMRs across all cell type pairs in the sn-m3C-seq dataset and 71 DMRs in the snmCAT-seq dataset. In contrast, imputed datasets with various filtering thresholds (0.2, 0.3, and 0.4) yielded tens to hundreds of thousands of DMRs. As expected, we observed a trend of higher DMR counts with less stringent filtering in both raw and 10% downsampled data (Figures S6 and S7; Table S9). For instance, with a filtering score of 0.2, averages of 45,185 and 43,684 DMRs were identified across all cell type pairs in the sn-m3C-seq and snmCAT-seq datasets, respectively, and these numbers increased to 128,287 and 135,970 with a filtering score of 0.4. Notably, comparable DMR counts were observed when scMeFormer was applied to the 10% downsampled data, demonstrating scMeFormer’s robust ability to detect cell-type-specific DMRs even under reduced coverage.

To estimate the false positive rates of our model in calling cell-type-specific DMRs, we turned to two broad cell types (neuron and glia) in the sn-m3C-seq dataset that allows us to identify CpG sites of sufficient coverage for identifying DMSs in the raw data. Specifically, we first collected CpG sites not within blacklist regions^20^ but with high coverage (≥100 reads) in each broad cell type to derive ground-truth labels for DMRs. In total, we detected 1,163,204 DMRs between two broad cell types, which were considered true positives. We then performed DMR analysis between the two cell types using imputed data, both without filtering and with filtering, at three score thresholds (0.2, 0.3, and 0.4). Remarkably, we observed higher precision rates with more stringent filtering, achieving a precision of 0.8 at a filtering score of 0.2 (Table S10). In contrast, the precision from imputed data without filtering was only 0.55. These results highlight the utility of filtering scores in enhancing the precision of DMR detection.

To assess the biological relevance of cell-type-specific DMRs identified from imputed data, we leveraged histone mark (H3K27ac) data that indicate active enhancers in four broad cell types (neuron, astrocyte, oligo, and microglia).^21^ Given prior evidence that regulatory regions are associated with low DNAm levels, we hypothesized that reliable cell-type-specific DMRs would be enriched for H3K27ac marks in the corresponding broad cell type. Our analysis confirmed this hypothesis, revealing that cell-type-specific DMRs were enriched for H3K27ac-marked regions in the corresponding broad cell type across both datasets (Figures S8 and S9; Table S11). Notably, this enrichment pattern remained consistent even with scMeFormer applied to the 10% downsampled data. This enrichment pattern became stronger when DMRs were identified from imputed data with a more stringent filtering score, reassuring the reliability of the filtering metric.

**Figure 6:**
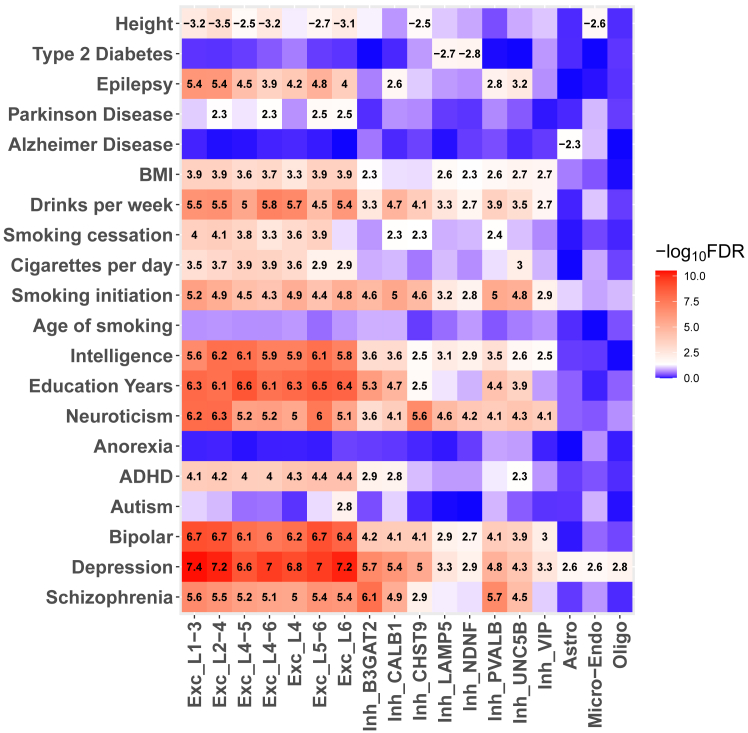
Heritability enrichment analysis for cell-type-specific DMRs identified from imputed sn-m3C-seq dataset The x axis represents cell types for which DMRs were defined relative to all other cell types, while the y axis lists various GWAS traits. The color scale represents the −log_10_(FDR) values derived from the *Z* score of per-SNP heritability calculated by stratified linkage disequilibrium score regression (LDSC). The white color denotes FDR = 0.05. Numbers within the squares show *Z* scores of per-SNP heritability that are significant after multiple testing correction (FDR < 0.05). Significant negative *Z* scores indicate a depletion of heritability. See also Figure S10 and Table S12.

To further validate the biological relevance of cell-type-specific DMRs identified from imputed data, we examined their enrichment for the heritability of 18 brain-related traits and two non-brain traits, human height and type 2 diabetes. The rationale for this analysis was that cell-type-specific DMRs enriched for regulatory regions in particular cell types should also enrich the heritability for traits related to those cell types. This was indeed observed for DMRs detected in both sn-m3C-seq and snmCAT-seq datasets (Figure S10; Table S12). Figure 6 shows the heritability enrichment analysis results from the imputed sn-m3C-seq dataset with a filtering score of 0.3, showing substantial enrichment for most brain-related traits and disorders, but not for height or diabetes, in DMRs specific to neuronal cell types, especially excitatory subtypes. In contrast, weak to no enrichment was observed for DMRs specific to non-neuronal cell types across all brain disorders except for depression, where we noted enrichment for DMRs specific to astrocytes, oligodendrocytes, and microglia or endothelial cell types, supporting the emerging role of glial cells in depression.^22^^,^^23^^,^^24^ This pattern remained consistent in the snmCAT-seq dataset and was robust across different filtering scores (0.2 and 0.4) for imputed data (Figure S10).

### scMeFormer enhances the detection of SCZ-associated DMRs

We applied scMeFormer to impute CpG states in 2,534 single-nucleus DNAm profiles generated from the prefrontal cortex of four patients with SCZ and four neurotypical controls. We first evaluated whether imputed data could recover clusters derived from CpHs, which is better suited for clustering neuronal cell types than CpGs.^4^ Specifically, using DNAm levels of CpHs in nonoverlapping 100 kb bins, we first clustered these nuclei into five major cell types: two excitatory neuron subtypes from the superficial (SupExc) and deep cortical (DeepExc) layers, two inhibitory neuron subtypes from the cortical ganglionic eminence (InhCGE) and the medial ganglionic eminence (InhMGE), and a glial cell type. We then clustered the same set of nuclei using imputed DNAm levels of CpGs in nonoverlapping 100 kb bins. Clusters from imputed data closely mirror the clusters obtained using CpHs (Figure S11), indicating that the imputation process preserved data properties related to cell type identity.

**Figure 7:**
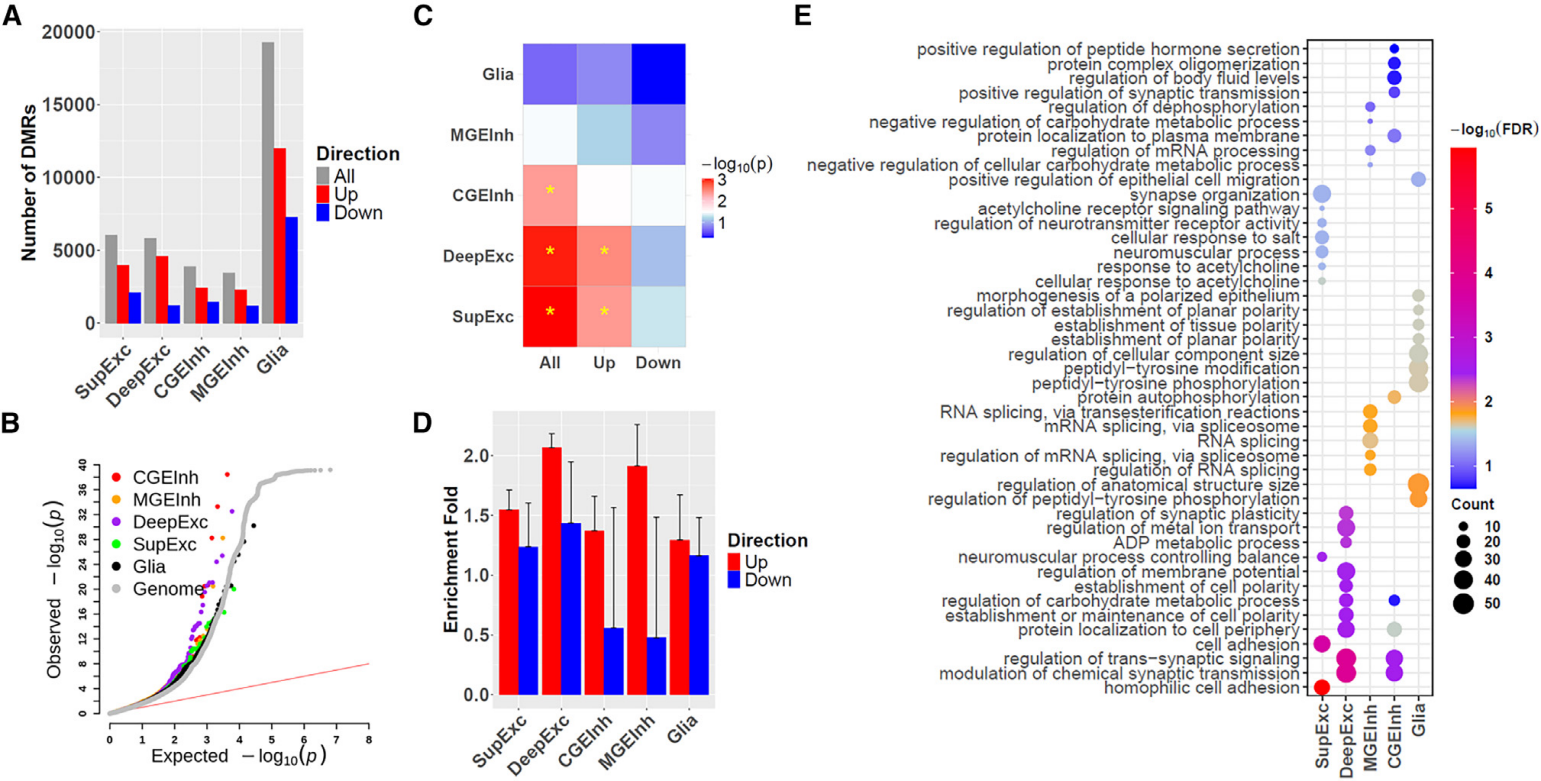
scMeFormer enhances the detection of SCZ-associated DMRs (A) The number of SCZ-associated DMRs detected in each cell type, stratified by direction of effect. (B) Quantile-quantile (Q-Q) plot of SCZ GWAS *p* values for SNPs located within or near DMRs detected in each cell type. (C) Gene-set-based association with SCZ for genes linked to DMRs in each cell type, where “up” and “down” on the x axis represent genes linked to up-regulated and down-regulated DMRs, respectively, and “all” represents genes linked to DMRs regardless of the direction of the effect. (D) Enrichment fold of genes linked to DMRs for differentially expressed genes identified in a single-cell RNA-seq study of SCZ. Error bars indicate the standard error of enrichment fold. (E) Top 10 enriched Gene Ontology terms for genes linked to DMRs through chromatin loops. See also Figures S11 and S12 and Tables S13–S15.

Next, we aimed to identify SCZ-associated DMRs within each cell type. Remarkably, while no DMRs were detected before imputation, imputation revealed thousands of DMRs across the five cell types (Figure 7A; Table S13). Notably, the number of up-regulated DMRs (indicating increased DNAm levels in SCZ) was substantially higher than the number of down-regulated DMRs. This observation aligns with the recent single-cell study of the SCZ transcriptome,^25^ which found that most dysregulated genes in SCZ were down-regulated, given the negative correlation between DNAm and gene expression. Further, we observed that SNPs within or near DMRs (within 5 kb) showed stronger enrichment for SCZ genome-wide association study (GWAS) signals,^26^ particularly in neuronal cell types, compared to SNPs across the genome (Figure 7B). This trend was primarily driven by up-regulated DMRs and less so by down-regulated DMRs (Figure S12), suggesting a pivotal role of increased DNAm in SCZ pathogenesis.

To assess the regulatory impact of these DMRs, we linked them to potential target genes using cell-type-specific chromatin loops identified in a previous study.^27^ We then conducted gene set analysis with MAGMA^28^ to determine whether these target genes were enriched for SCZ GWAS signals. Our analysis revealed significant enrichment of SCZ GWAS signals among genes linked to DMRs in two excitatory neuron subtypes (SupExc and DeepExc) and one inhibitory neuron subtype (InhCGE) (false discovery rate [FDR] < 0.05) (Figure 7C; Table S14). Stratified analysis by DMR direction indicated that this enrichment was primarily driven by genes linked to up-regulated DMRs, further supporting the role of increased DNAm in SCZ pathogenesis.

We further explored whether genes linked to DMRs were enriched for differentially expressed genes (DEGs) from a recent single-cell RNA sequencing (RNA-seq) study of SCZ,^25^ matched by the five broad cell types defined in our study. We found that genes linked to up-regulated DMRs were significantly enriched for down-regulated DEGs in the two excitatory neuron subtypes; however, no such enrichment was observed for other cell types, nor was there enrichment for up-regulated DEGs among genes linked to down-regulated DMRs (Figure 7D; Table S15). Gene Ontology enrichment analysis further revealed that genes linked to DMRs were enriched for synaptic signaling, particularly in the two excitatory neuron subtypes and one inhibitory neuron subtype (InhCGE) (Figure 7E; Table S16). Analysis by DMR direction suggested that this enrichment was primarily driven by genes linked to up-regulated DMRs (Table S16). Together, these findings from GWASs, DMRs, and DEGs highlight the critical role of excitatory neurons in SCZ, potentially mediated by up-regulated DMRs.

Finally, we compiled all candidate genes linked to up-regulated DMRs in each cell type that also showed evidence of differential expression (*p*_adj_ < 0.05), along with their GWAS signals (MAGMA gene-based *p* value), generating a list of genes for further investigation (Table S17). This list includes well-established genes implicated in SCZ that showed converging evidence—up-regulated DMRs, down-regulated DEGs, and significant gene-based *p* values from MAGMA (<2.5 × 10^−6^), such as *GRIN2A* (glutamate ionotropic receptor NMDA type subunit 2A), *RBFOX1* (RNA binding fox-1 homolog 1), and *NCAM1* (neural cell adhesion molecule 1).

## Discussion

Single-cell DNAm profiling technologies provide unprecedented opportunities to explore the epigenetic landscape at single-cell resolution. However, many of these technologies suffer from a high missing rate of CpG sites, limiting their full potential to uncover the epigenetic mechanism underlying various biological processes and diseases. Previous deep learning models have attempted to impute CpG methylation status in single cells.^13^^,^^14^^,^^15^ However, these models face challenges in scaling to thousands of cells, a scenario that is becoming increasingly popular. In this study, we introduced scMeFormer, a transformer-based deep learning model designed to efficiently impute DNAm states across thousands of cells. Unlike earlier models, scMeFormer scales effectively to large datasets, achieving training times of approximately 72 h per single-nucleus DNAm dataset if trained from scratch using four NVIDIA A100 GPUs. Notably, the architecture of scMeFormer allows it to be initially trained on a single dataset and then fine-tuned for new datasets or even across species. This transfer learning strategy maintains robust performance with minimal loss in predictive accuracy compared to training from scratch while significantly reducing computational time; fine-tuning each dataset took only about 6 h in our study. In contrast, training previous deep learning models (DeepCpG, Transformer CpG, and GraphCpG) in our benchmarking was highly computationally intensive, requiring approximately 72 h to converge, despite being trained on just 118 cells selected from the sn-m3C-seq dataset. We further estimated the time required for each model to be trained for 10 epochs as the number of cells increased. While the training times for DeepCpG, Transformer CpG, and GraphCpG scaled linearly or near exponentially with the number of cells, scMeFormer exhibited a near-constant training time, remaining largely unaffected by cell numbers (Table S18). These findings underscore scMeFormer’s superior accuracy and efficiency for large-scale single-cell DNAm analysis.

Remarkably, scMeFormer exhibits the ability to impute DNAm states with high fidelity, even when coverage was reduced to only 10% of CpG sites through downsampling, as evidenced by its successful recovery of cell type clusters and identification of cell-type-specific DMRs. Furthermore, we designed a unique filtering score that enables quality control of the imputed data: more stringent filtering scores yield higher-quality imputed data, though they reduce the number of CpG sites imputed in each cell. As a case study, we applied scMeFormer to a single-nucleus DNAm dataset generated from the prefrontal cortex of patients with SCZ and neurotypical controls. This led to the identification of thousands of SCZ-associated DMRs that would have remained undetectable without imputation. Most of these DMRs were up-regulated in patients and showed enrichment for SCZ GWAS signals and down-regulated DEGs in excitatory neurons, underscoring the critical role of excitatory neurons in SCZ.

While scMeFormer is specifically designed for single-cell DNAm data imputation, the foundational principles and modular architecture of our model have potential applications across other single-cell omics data. scMeFormer comprises two main modules that capture both local patterns and long-range dependencies—features that are common across various omics modalities. For instance, in single-cell Hi-C data, the modules could be reconfigured to learn chromatin contact patterns from DNA sequences and borrow information of neighboring contacts within and across cell types. Similarly, for single-cell ATAC-seq data, the architecture could be tailored to model accessible regions and their potential interactions. The transformer component of scMeFormer is particularly valuable due to its ability to model long-range dependencies, making it well suited for capturing complex interactions such as enhancer-promoter loops. Future work exploring these adaptations could expand the utility of scMeFormer, making it a valuable tool in the broader field of single-cell omics research.

### Limitations of the study

We acknowledge four limitations of our current model and potential areas for future work. First, scMeFormer relies on input DNA sequences from the reference genome, which does not precisely align with the DNA sequences of the study samples. The model’s performance could be further improved by utilizing DNA sequences and DNAm data from the same individuals. Second, the imputation quality metric relies on the assumption that target and neighboring CpG sites show consistent methylation levels. However, this may not always hold, particularly in regions with heterogeneous methylation patterns. Developing more robust evaluation metrics will be an important direction of future work. Third, while scMeFormer demonstrated strong overall performance, it faced challenges in imputing CpG sites with high variability across cells. Additionally, we observed noticeably stronger performance in lowly methylated regions (e.g., TssA and bivalent regions) compared to highly methylated regions (e.g., Tx and Quies). This discrepancy may indicate potential systemic biases or intrinsic complexities related to transcription-linked methylation biology, highlighting the need for further investigation. Fourth, scMeFormer currently focuses on CpG sites imputation. Expanding its capabilities to CpH sites, known for their crucial roles in neurons, would enhance its utility. Despite these limitations, our study demonstrates the power of deep learning for single-cell DNAm state imputation, and we anticipate that scMeFormer will serve as a valuable tool for advancing single-cell DNAm studies.

## Resource availability

### Lead contact

Requests for further information and resources should be directed to and will be fulfilled by the lead contact, Shizhong Han (Shizhong.Han@libd.org).

### Materials availability

This study did not generate new unique reagents.

### Data and code availability

• This paper analyzes existing, publicly available data. The accession numbers for the datasets are listed in the key resources table.
• Processed single-nucleus DNAm data generated in this study have been deposited into GEO (GEO: GSE285847).
• All original code has been deposited at GitHub (https://github.com/LieberInstitute/scMeformer) and is publicly available. A permanent DOIs from Zenodo (https://doi.org/10.5281/zenodo.14727350) is listed in the key resources table.
• Any additional information required to reanalyze the data reported in this paper is available from the lead contact upon request.

## Acknowledgments

We are grateful for the vision and generosity of the Lieber and Maltz families, who made this work possible, and the families who donated to this research and for computing support from the Joint High Performance Computing Exchange (JHPCE) facility in the Department of Biostatistics at the Johns Hopkins Bloomberg School of Public Health. We would like to thank the two anonymous reviewers for their thoughtful criticisms, comments, and suggestions on early versions of the manuscript. This work is supported by 10.13039/100000002National Institutes of Health grants R01MH121394 (S.H.) and R01MH112751 (S.H.).

## Author contributions

Conceptualization, J.Z., D.R.W., and S.H.; methodology, J.Z., C.L., and S.H.; investigation, J.Z., C.L., H.L., M.G.H., R.E.S., and S.H.; visualization, J.Z., M.G.H., and S.H.; funding acquisition, S.H.; project administration, S.H.; supervision, S.H.; writing – original draft, J.Z. and S.H.; writing – review & editing, J.Z., C.L., H.L., M.G.H., R.E.S., J.E.K., T.M.H., J.R.E., D.R.W., and S.H.

## Declaration of interests

D.R.W. serves on the scientific advisory boards of Sage Therapeutics and Pasithea Therapeutics. J.R.E. serves on the scientific advisory board of Zymo Research.

## STAR★Methods

### Key resources table

**Table:** 

REAGENT or RESOURCE	SOURCE	IDENTIFIER
**Biological samples**		

Postmortem prefrontal cortex samples	LIBD brain repository	N/A

**Deposited data**		

Processed single-nucleus DNAm data	This study	GEO: GSE285847
Human single-nucleus DNAm data (snmC-seq)	Luo et al.^4^	GEO: GSE97179
Human single-nucleus DNAm data (snmC-seq2)	Luo et al.^6^	GEO: GSE112471
Human single-nucleus DNAm data (sn-m3C-seq)	Lee et al.^5^	GEO: GSE130711
Human single-nucleus DNAm data (snmCAT-seq)	Luo et al.^7^	GEO: GSE140493
Mouse single-nucleus DNAm data (scNMT-seq)	Argelaguet et al.^16^	GEO: GSE121708
schizophrenia GWAS summary statistics	Trubetskoy et al.^26^	https://pgc.unc.edu/for-researchers/download-results/
schizophrenia cell type-specific DEGs	Ruzicka et al.^25^	Supplementary tables: https://www.science.org/doi/10.1126/science.adg5136

**Software and algorithms**		

scMeFormer	This study	GitHub: https://github.com/LieberInstitute/scMeformer Zenodo https://doi.org/10.5281/zenodo.14727350
GraphCpG	Deng et al.^15^	https://github.com/yuzhong-deng/graphcpg
CpG Transformer	De Waele et al.^14^	https://github.com/gdewael/cpg-transformer
DeepCpG	Angermueller et al.^13^	https://github.com/cangermueller/deepcpg
CaMelia	Tang et al.^10^	https://github.com/JxTang-bioinformatics/CaMelia
methylpy	Schultz et al.^29^	https://github.com/yupenghe/methylpy
S-LDSC	Finucane et al.^30^	https://github.com/bulik/ldsc
YAP	Luo et al.^7^	https://cemba-data.readthedocs.io/en/latest/
scanorama	Hie et al.^31^	https://github.com/brianhie/scanorama
MAGMA	de Leeuw et al.^28^	https://cncr.nl/research/magma/
clusterProfiler	Yu et al.^32^	https://github.com/YuLab-SMU/clusterProfiler

### Experimental model and subject details

We generated single-nucleus methylomes from the prefrontal cortex of four SCZ cases and four neurotypical controls using the snmCAT-seq technique, but without capturing chromatin accessibility information. The brain tissues were from the brain repository at the Lieber Institute for Brain Development. Details on tissue acquisition, processing, curation, and dissection, were described in prior reports.^33^ All eight brain samples were male Caucasian individuals with a mean age of 42 years old in both cases and controls.

### Method details

#### Single-nucleus DNAm datasets used for model training

Datasets include four single-nucleus DNAm datasets generated by four different technologies we developed: snmC-seq,^4^ snmC-seq2,^6^ sn-m3C-seq,^5^ and snmCAT-seq,^7^ all applied to human postmortem brain tissues. To show generalizability of our model, we also included a single-nucleus dataset from mouse embryos generated by scNMT-seq.^16^ Detailed information regarding each technology and the bioinformatics procedures for data processing have been described in original studies. Briefly, snmC-seq was a multiplexed single-nucleus DNAm profiling technique we initially developed, which was used to analyze the methylomes of 2,784 neurons isolated from the human frontal cortex. snmC-seq2 was the improved version of snmC-seq with increased read mapping and enhanced throughput, and was used to profile the methylomes of 3,072 nuclei obtained from postmortem prefrontal cortex. sn-m3C-seq was a single-cell multi-omics technique that jointly profiles chromatin conformation and DNAm from the same cells and was applied to profile 4,237 nuclei from human BA10 cortical tissue. snmCAT-seq was developed to jointly profile methylome, chromatin accessibility, and transcriptome from the same cells, and was applied to profile 4,357 nuclei isolated from postmortem human BA10 cortical tissue. scNMT-seq was a single-cell multi-omics technique designed to profile nucleosome positioning, DNAm, and transcriptomes simultaneously. This technique was applied to profile 1,105 single cells isolated from mouse embryos across four developmental stages, capturing the transition from pluripotency to primary germ-layer specification.

#### scMeformer architecture

scMeformer contains three main modules: a DNA module, a CpG module, and a fully connected network. The input to the model includes two modalities: a one-hot encoded DNA sequence of 2 kb and the DNAm levels of 100 neighboring CpGs around the target CpG in each cell cluster. The output of the model is the predicted methylation states of the target CpG in each cell across all cells. Table S19 provides details for key hyperparameters used in the model. Below are descriptions of each module.

#### DNA module

The DNA module employs the INTERACT architecture developed in our previous study,^34^ which consists of two sub-modules: a convolutional neural network (CNN) and the encoder of transformer. The CNN comprises three convolutional layers, a batch normalization layer, a max-pooling layer, and a dropout layer. Each convolutional layer uses 256 kernels of size 10 to learn motifs from DNA sequences, and each kernel is activated by a rectified linear unit (ReLU) function. The max-pooling layer (step size = 20bp) is used after the three convolutional layers to capture motifs learned by the convolutional layers. The batch normalization layer is used after the max-pooling layer to improve training speed and stability. The dropout layer is employed to prevent overfitting. The dropout rate in this layer is set to 0.5.

The encoder of transformer consists of a stack of eight identical layers, which takes the CNN output as input to learn distant features that may act jointly. Each layer in the transformer encoder employs two sub-layers. The first sub-layer is a multi-head self-attention layer that learns the attention between any two features at different positions. The second sub-layer is a simple, position-wise fully connected feedforward network. After both sub-layers, a normalization layer is employed to speed up training and improve training stability. Additionally, a dropout layer with a rate of 0.1 is employed to prevent overfitting. Each sub-layer in the encoder has a residual connection to help mitigate the vanishing gradient problem. Residual connections are often used in deep neural networks to prevent the network from forgetting important features of the input during training.

#### CpG module

The CpG module employs a similar architecture as the DNA module. Given a target CpG site the model aims to predict, the CpG module takes as input the DNAm levels of 100 CpG sites around the target CpG site (excluding the target CpG site itself) across pre-defined cell clusters. To create this input, all single cells are initially clustered into clusters based on the DNAm levels of CpG sites within non-overlapping 100kb bins. For a given CpG site, its DNAm level in each cluster is determined by dividing the number of methylated reads by the total number of reads in that cluster. If the cells are clustered into *n* clusters, the input to the CpG module is a 100 × n matrix. The CNN identifies local patterns of CpG methylation within each cluster through a 1-dimensional convolutional kernel (256 kernels), generating a feature matrix of dimensions 256 × K for each cluster. The mean feature matrix across clusters is then fed into a position-wise transformer to capture relationships among positions. The feature matrix at the position of target CpG is passed to the cluster-wise transformer to capture relationships among cell clusters.

#### Fully connected network

The fully connected network comprises a hidden layer with 768 units, a dropout layer, and an output layer. The dropout layer is designed to prevent overfitting and uses a drop rate of 0.1. The output layer applies the sigmoid function to scale the predicted values between 0 and 1. The number of units in the output layer equals the number of cells. The input to the fully connected network is the concatenation of the output from the DNA module and the CpG module. Each unit in the output layer represents the DNAm state of the target CpG site in the corresponding cell and is indicated by a value of either 0 or 1.

#### Model training

We divided CpG sites into three subsets by chromosomes for model training, validation, and evaluation. The training set consisted of CpG sites on chromosomes 1 to 20, while CpG sites on chromosome 21 were used as the validation set for model tuning, and CpG sites on chromosome 22 were used as the independent testing set to evaluate the model prediction performance. Approximately 5% of CpG sites were covered by at least one read in a single cell across the five datasets and were employed for model training, while the remaining CpG sites were not covered by any reads and were not used in model training. We defined DNAm state as 1 for a CpG site if all mapped reads support methylation, and 0 if all mapped reads support unmethylation. We did not include CpG sites for model training if their mapped reads support both methylation and unmethylation. We employed two training strategies for scMeFormer. The first strategy involved training a separate model from scratch for each dataset (scMeFormer(scratch)). The second strategy involved pre-training a model on one dataset, followed by fine-tuning it on a different dataset (scMeFormer(finetune)). Specifically, for scMeFormer(finetune), we pre-trained the model on the snmCAT-seq dataset and fine-tuned it on the snmC-seq and sn-m3C-seq datasets; we also pre-trained the model on the sn-m3C-seq dataset and fine-tuned it on snmC-seq2, snmCAT-seq, and mouse embryo datasets.

#### Comparison with alternative models

We compared scMeformer to four alternative models: the CNN model, the cluster model, the scMeformer model but with only the DNA module, and the scMeformer model but with only the CpG module. The CNN model also consists of three modules: a DNA module, a CpG module, and a fully connected network. However, in the CNN model, both the DNA and CpG modules employ a convolutional neural network (the same as described in the DNA module of scMeformer) rather than the transformer encoders. The cluster model first clusters cells into clusters based on DNAm levels of CpGs within non-overlapping 100kb bins. For each cell in a cluster, the methylation state for a CpG site not covered by any reads is imputed by known methylation states of this CpG site in cells of the same cluster.

We further compared scMeFormer with three prior deep learning models GraphCpG,^15^ CpG Transformer^14^*,* DeepCpG,^13^ and CaMelia,^10^ a gradient boosting-based model. Due to the scalability limitation of these models, we trained them on a randomly selected subset of 118 cells from the sn-m3C-seq dataset. All models were trained using the same setup for the training, validation, and test sets of CpG sites as used for scMeFormer. To estimate the training time required for larger datasets, we ran each model for a limited number of batches at varying number of cells from the sn-m3C-seq dataset, recorded the time, and extrapolated it to estimate the time needed for 10 epochs.

### Quantification and statistical analysis

#### Cell type-specific DMRs

We leveraged pre-assigned cell type labels for each nucleus in each dataset from the original studies. We then employed the DMRfind function from methylpy (v1.4.2)^29^ to identify cell type-specific DMRs across all cell type pairs. Briefly, DMRfind utilizes a permutation-based root-mean-square test of goodness-of-fit to identify differentially methylated sites (DMS) across samples. Consecutive DMSs within 250 bp are then merged into DMRs. In our imputed dataset, we considered a CpG site with a predicted methylation status as covered by one read supporting methylation, and a predicted unmethylated CpG site as covered by one read supporting unmethylation.

#### Stratified LD score regression

We performed stratified LD score regression (S-LDSC)^30^ to evaluate the enrichment of heritability of 18 brain-related traits and disorders. We also included two non-brain traits, human height and type 2 diabetes, as a negative control to examine whether our findings are specific to brain disorders. We downloaded GWAS summary statistics of each trait from the sources listed in Table S20. Following recommendations from the LDSC resource website (https://alkesgroup.broadinstitute.org/LDSCORE), S-LDSC was run for each list of variants with the baseline LD model v2.2 that included 97 annotations to control for the LD between variants with other functional annotations in the genome. We used HapMap Project Phase 3 SNPs as regression SNPs, and 1000 Genomes SNPs of European ancestry samples as reference SNPs, downloaded from the LDSC resource website. To evaluate the unique contribution of annotations to trait heritability, we utilized a metric from S-LDSC: the *Z* score of per-SNP heritability. This metric allows us to discern the unique contributions of candidate annotations while accounting for contributions from other functional annotations in the baseline model. The *p*-values were derived from the *Z* score under the assumption of a normal distribution, using a two-tailed test. FDR was computed from the *p*-values using the Benjamini & Hochberg procedure.

#### Single-nucleus DNAm data from schizophrenia cases and controls

We processed sequencing reads by implementing a versatile mapping pipeline (http://cemba-data.readthedocs.io/) for all the methylome-based technologies developed by our group, as detailed in our previous study.^7^ After allc files were generated, the methylcytosine (*mc*) and total cytosine basecalls (*cov*) were summed up for each 100kb bin across the hg19 genome for each sequence context (CG, CH). After filtering cells by various mapping metrics, 2,534 nuclei were retained for further analysis. Using DNAm levels of CpHs in nonoverlapping 100kb bins, we clustered these nuclei into five major cell types: two excitatory neuron subtypes from the superficial and deep cortical layers (SupExc and DeepExc), two inhibitory neuron subtypes from the cortical ganglionic eminence (InhCGE) and the medial ganglionic eminence (InhMGE), and a glial cell type. We also clustered these nuclei using DNAm levels of CpGs in nonoverlapping 100kb bins after imputation employing functions from the scanorama package.^31^ We imputed CpGs DNAm states in each cell through fine-tuning the model trained on sn-m3C-seq dataset for each sample we collected for DNAm. We applied a filtering score of 0.3 to the imputed data to enhance its quality, balancing precision and recall based on our evaluation for precision using two broad cell types. We employed the DMRfind function from methylpy to identify SCZ-associated DMRs for each cell type. DMRs were called for regions with at least two DMSs (FDR <0.01) within 250bp, and each DMS had the same direction of effect in at least two samples from either the case or control group.

#### Assignment of DMRs to target genes

SCZ-associated DMRs detected in each cell type were assigned to target genes by leveraging reported cell type-specific chromatin loops detected in the prefrontal cortex in a previous study.^27^ Given the limited number of loops detected in each individual cell type, we combined loops from cell types belonging to the broad neuronal or glial cell types. We then linked DMRs in neuronal cell types (DeepExc, SupExc, CGEInh, MGEInh) to their potential target genes based on chromatin loops collected for the broad neuronal cell type. Similarly, DMRs in glial cell types were linked to potential target genes using loops collected for the broad glial category. We used clusterProfiler^32^ for gene ontology enrichment analysis for genes regulated by DMRs detected in each cell type.

#### Gene-set analysis of SCZ GWAS signals

We used MAGMA^28^ gene-set analysis to test for enrichment of SCZ GWAS signals for genes linked to SCZ-associated DMR detected in our dataset. SNPs were assigned to genes if they were located within gene boundaries, based on NCBI 37.3 gene annotation, or within 20 kb upstream or downstream of the gene, to capture regulatory variants. We first performed gene-based association test using SCZ PGC3 GWAS summary statistics of European ancestry samples,^26^ employing the “gene-wise” model of MAGMA. Linkage disequilibrium (LD) reference was based on the genotype data of unrelated EUR samples from the 1000 Genomes Project. Gene-set analysis were performed using MAGMA with the “--set-annot” option.

#### Enrichment analysis for SCZ-associated differentially expressed genes

To evaluate whether genes linked to SCZ-associated DMRs were enriched for SCZ-associated DEGs, we utilized DEG analysis results from a recent scRNA-Seq study of SCZ.^25^ To generate a DEG list for each broad cell type defined in our DNAm dataset, we aggregated DEGs (*p*_adj_ < 0.05) identified in the original cell types from the scRNA-Seq study that corresponded to each broad cell type. Fisher’s exact test was used to test the enrichment of DEGs among genes linked to DMRs in each broad cell type compared to the background gene set, which were defined as all loop-connected genes in the broad neuronal or glial cell types.

## Supplemental information

Document S1. Figures S1–S12 and Tables S1, S4, S10, S13–S15, S18, and S19

Table S2. Prediction performance of scMeFormer and alternative models across five single-nucleus DNAm datasets, related to Figures 2 and S1 Performance was evaluated on subsets of independent testing CpG sites, stratified by their levels of variation across all cells in each dataset. The “0–0.1” group represents the bottom 10% least variable CpG sites, while the “0.9–1.0” group represents the top 10% most variable CpG sites.

Table S3. Prediction performance of scMeFormer and alternative models across four single-nucleus DNAm datasets from the human brain, related to Figures 2 and S2 Performance was evaluated on subsets of independent testing CpG sites, stratified by their chromatin states defined in bulk dorsolateral prefrontal cortex derived from the ROADMAP Epigenomics Project.

Table S5. Model prediction performance of scMeformer and the cluster model across five datasets under lower CpG coverage through downsampling, related to Figures 3 and S4 Performance was evaluated based on subsets of independent testing CpG sites stratified by their levels of variations across all cells in each dataset. The “0–0.1” group represents the bottom 10% least variable CpG sites, while the “0.9–1.0” group represents the top 10% most variable CpG sites.

Table S6. Comparison of scMeFormer model prediction performance at different filtering score thresholds across five datasets, evaluated under various levels of reduced CpG coverage achieved through downsampling, related to Figure 4

Table S7. Evaluation of imputed data under reduced CpG coverage for its ability to recover cell types identified in the original data across four human datasets, related to Figure 5 "scMeFormer+Filter" represents imputed data filtered using a quality score threshold of 0.2.

Table S8. Recall rates for cell-type-specific DMRs detected in sn-m3C-seq and snmCAT-seq datasets, imputed from raw and 10% downsampled data with three filtering score thresholds (indicated in brackets), related to Figures 3, 4, and S5

Table S9. Log-transformed count of cell-type-specific DMRs detected in sn-m3C-seq and snmCAT-seq datasets from raw and imputed data under varying downsampling and filtering conditions, related to Figures 3, 4, S6, and S7 This table shows the natural log-transformed counts of cell type-specific DMRs detected in the sn-m3C-seq and snmCAT-seq datasets. DMRs were identified from raw data, as well as from imputed data based on either raw (100% coverage) or 10% downsampled data. For imputed data, three filtering score thresholds were applied (indicated in brackets) to assess the effect of filtering on DMR detection across different downsampling levels.

Table S11. Enrichment of the H3K27ac mark in the corresponding broad cell types among cell-type-specific DMRs identified in the sn-m3C-seq and snmCAT-seq datasets, using imputed data under different downsampling and filtering conditions, related to Figures 3, 4, S8, and S9

Table S12. Heritability enrichment analysis for cell-type-specific DMRs detected in sn-m3C-seq and snmCAT-seq datasets from imputed data under varying filtering conditions, related to Figures 6 and S10

Table S16. Gene Ontology enrichment analysis for genes linked to SCZ-associated DMRs through chromatin loops, related to Figure 7 Only enriched terms with a q-value < 0.1 are showed.

Table S17. Compiled list of candidate genes linked to up-regulated DMRs in each cell type that also show evidence of differential expression (*p*_adj_ < 0.05), along with their GWAS signals (MAGMA gene-based *p* values), related to Figure 7 To derive DEGs for each broad cell type defined in our DNAm dataset, we aggregated DEGs (padj < 0.05) identified in the original cell types from the scRNA-Seq study corresponding to each broad cell type. If a DEG appeared in multiple cell types within a broad cell type, we selected the DEG effect with the largest magnitude among those cell types.

Table S20. GWAS summary statistics for heritability enrichment analysis, related to Figure 6 and STAR Methods

Document S2. Article plus supplemental information
