# Supplementary material for: Deep learning imputes DNA methylation states in single cells and enhances the detection of epigenetic alterations in schizophrenia
Source: Cell Genom. 2025 Feb 21;5(3):100774. doi: 10.1016/j.xgen.2025.100774 (PMC11960545; doi:10.1016/j.xgen.2025.100774)
Supplement: Document S1. Figures S1–S12 and Tables S1, S4, S10, S13–S15, S18, and S19 [file mmc1.pdf]

Cell Genomics, Volume *5*

Supplemental information

Deep learning imputes DNA methylation states in single cells and enhances the detection of epigenetic alterations in schizophrenia

Jiyun Zhou, Chongyuan Luo, Hanqing Liu, Matthew G. Heffel, Richard E. Straub, Joel E. Kleinman, Thomas M. Hyde, Joseph R. Ecker, Daniel R. Weinberger, and Shizhong Han

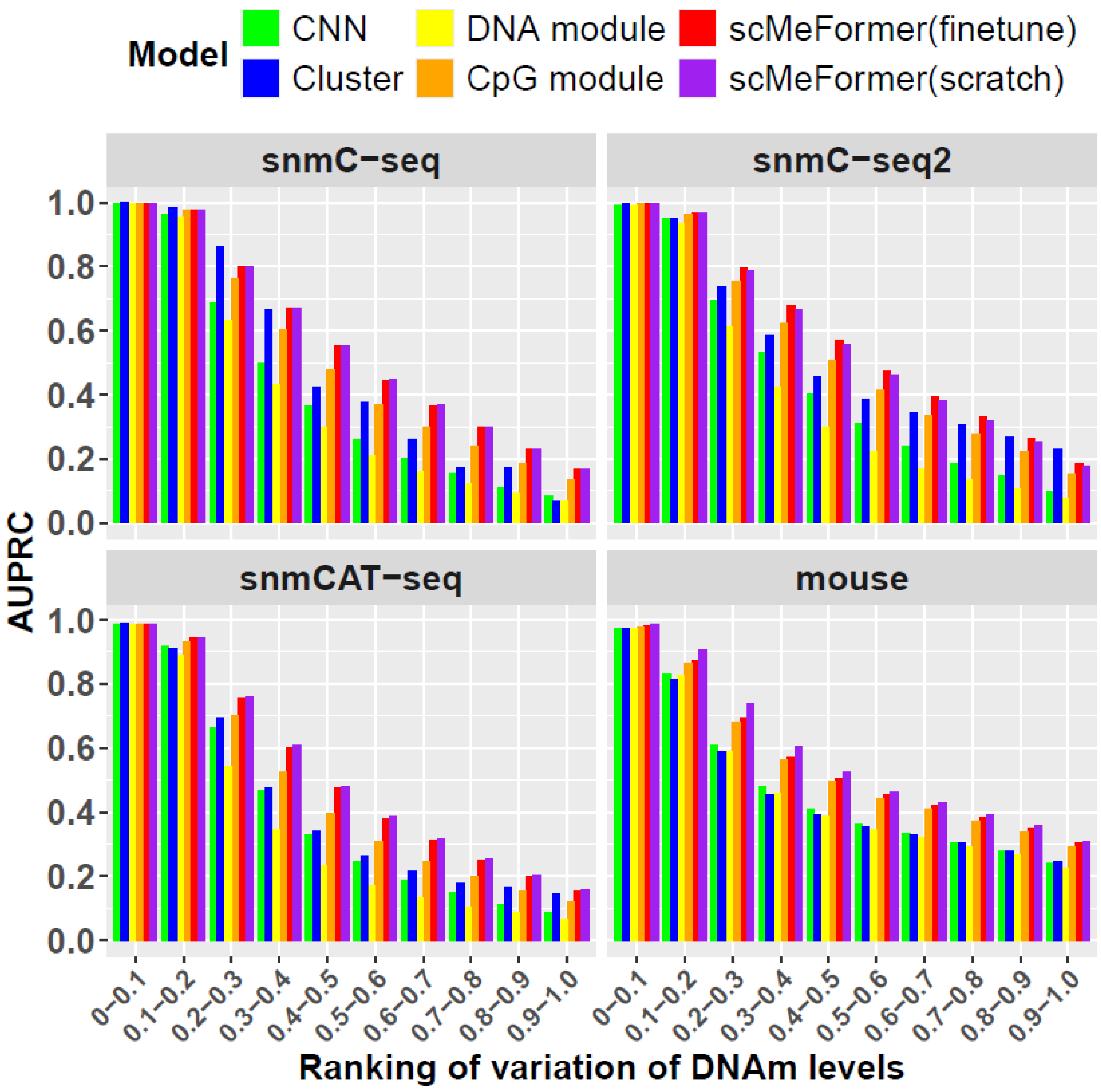

Figure S1. Comparison of model prediction performance between scMeFormer and four alternative models across four single-nucleus DNAm datasets, Related to Figure 2. Comparison was based on subsets of independent testing CpG sites, stratified by their levels of variations across all cells in each dataset. The “0-0.1” group represents the bottom 10% least variable CpG sites. The “0.9-1.0” group represents the top 10% most variable CpG sites.

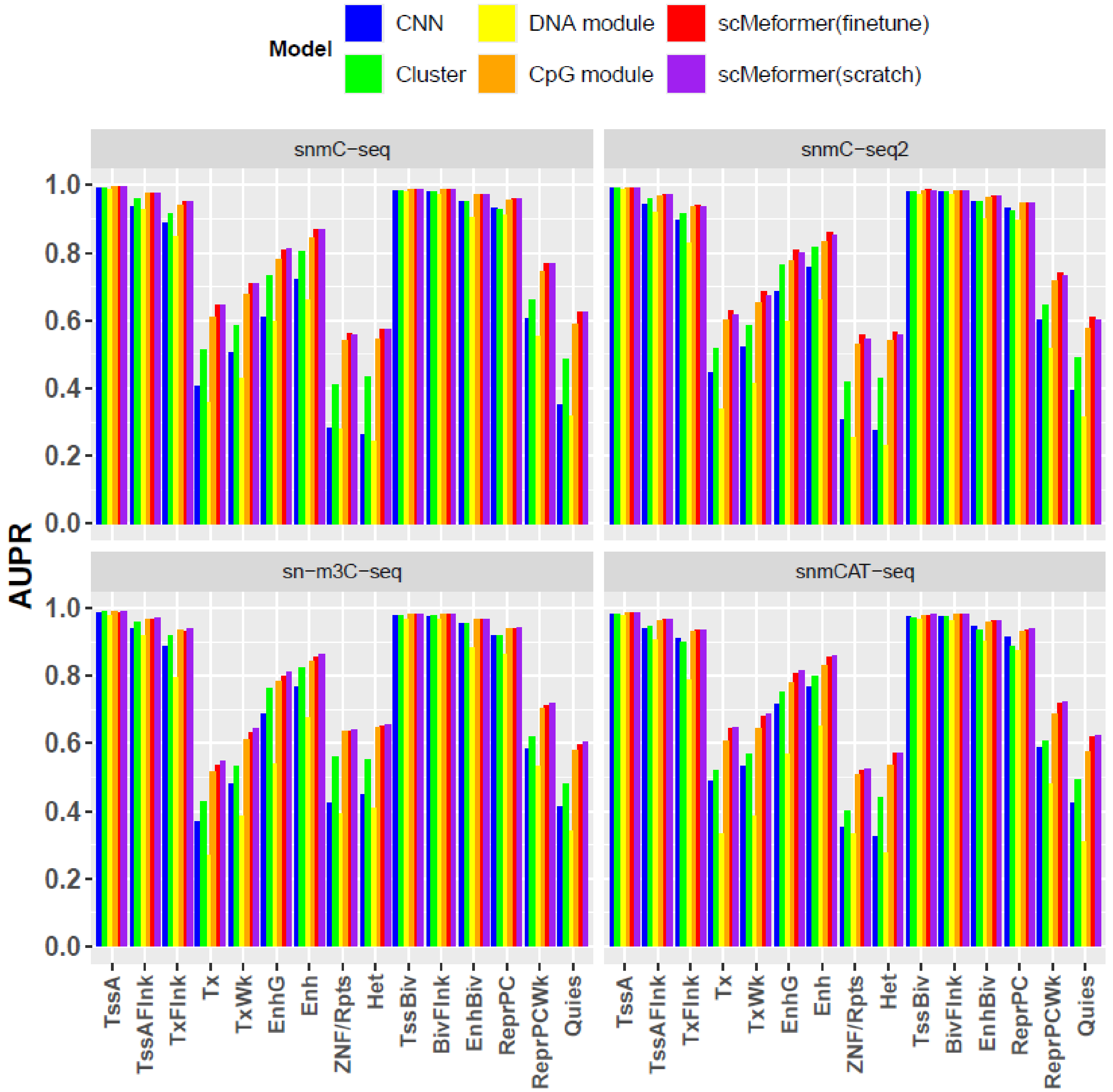

Figure S2. Prediction performance of scMeFormer and alternative models across four single-nucleus DNAm datasets from the human brain, Related to Figure 2 and Table S3. Performance was evaluated on subsets of independent testing CpG sites, stratified by their chromatin states defined in bulk dorsolateral prefrontal cortex derived from the ROADMAP Epigenomics Project.

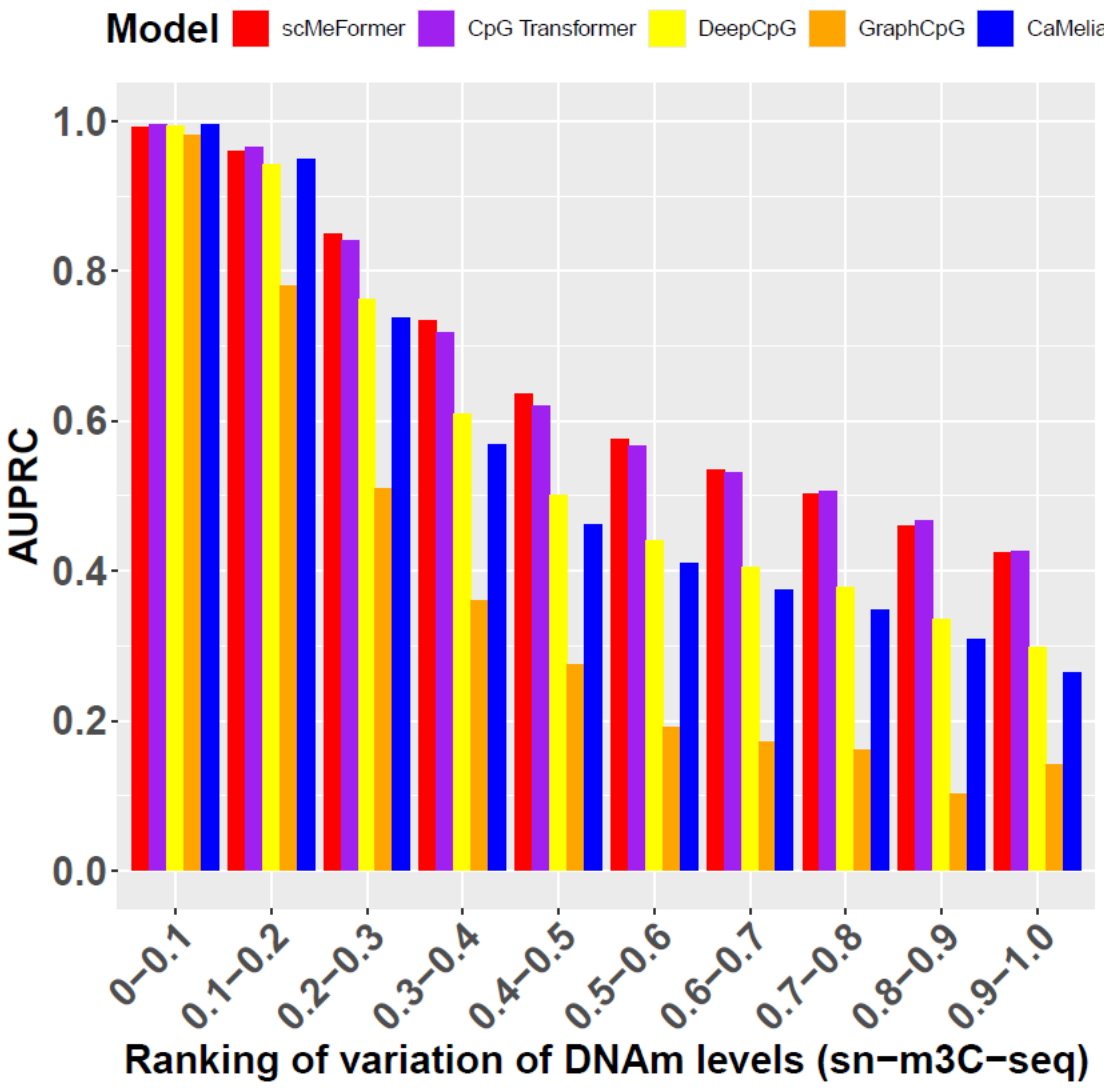

Figure S3. Comparison of model prediction performance between scMeFormer and three previous models (DeepCpG, GraphCpG, and CaMelia), Related to Figure 2. Comparison was based on subsets of independent testing CpG sites, stratified by their levels of variations across all cells in each dataset. The "0-0.1" group represents the bottom 10% least variable CpG sites. The "0.9-1.0" group represents the top 10% most variable CpG sites.

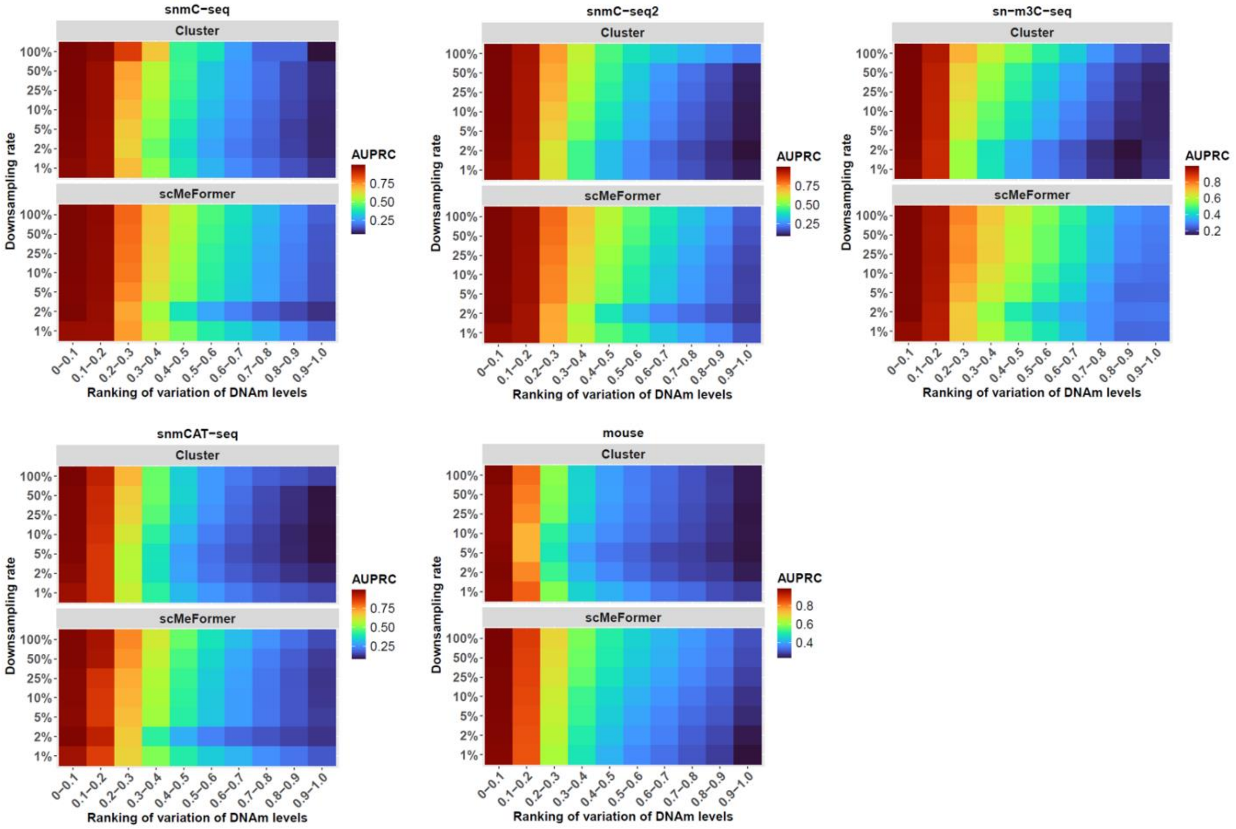

Figure S4. Comparison of prediction performance between scMeformer and the cluster model across five datasets under lower CpG coverage through downsampling, Related to Figure 3 and Table S5. Comparison was based on subsets of independent testing CpG sites, stratified by their levels of variation across cells. The “0-0.1” group represents the bottom 10% least variable CpG sites. The “0.9-1.0” group represents the top 10% most variable CpG sites.

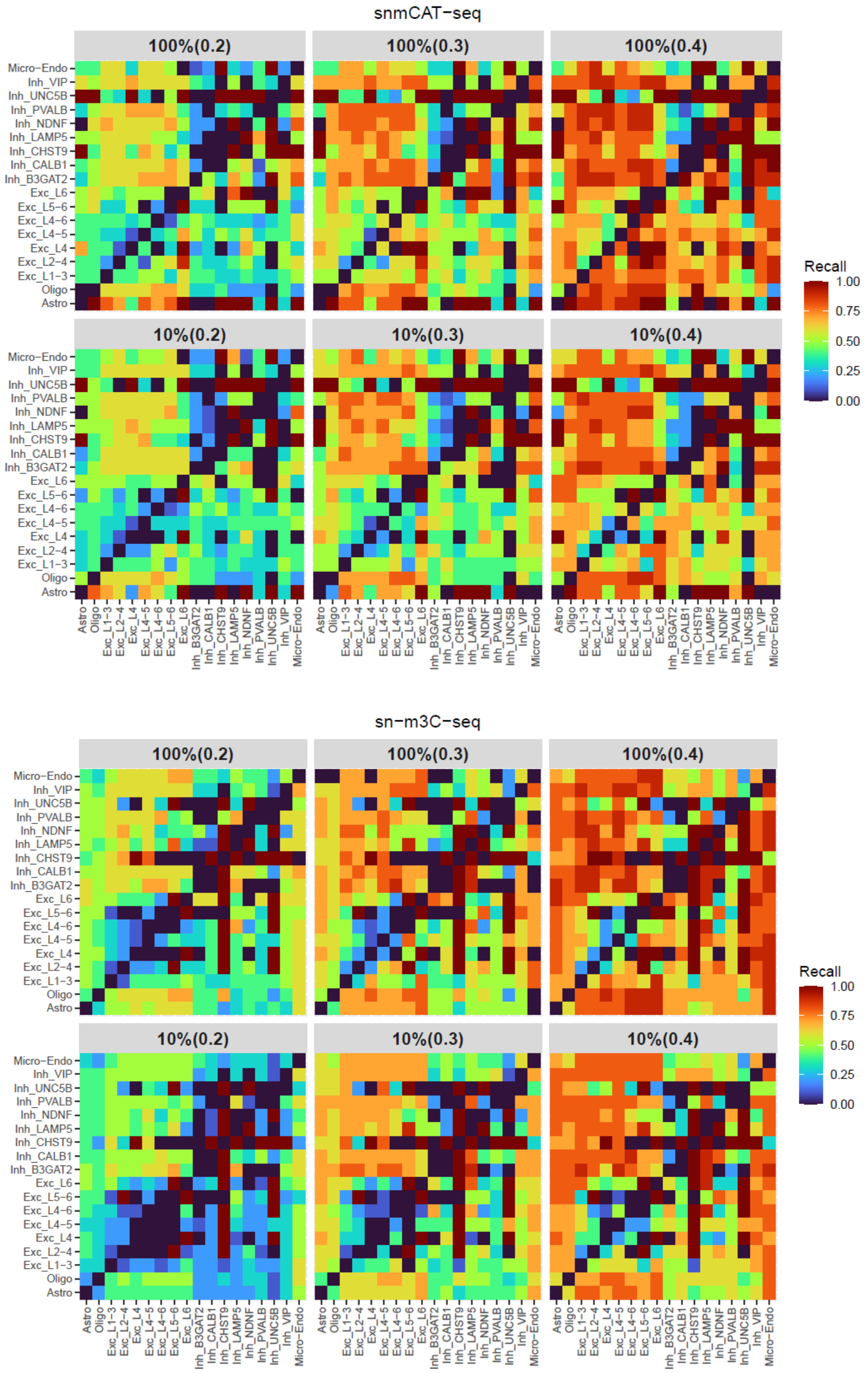

Figure S5. Recall rates for cell type-specific DMRs identified between each pair of cell types in the snmCAT-seq and sn-m3c-seq datasets, Related to Figures 3-4, Table S8. Cell type-specific DMRs were defined by the presence of at least two differentially methylated CpG sites (DMSs). The upper panel in each plot displays recall rates with imputation performed on the original, non-downsampled data, while the lower panel shows recall rates on data downsampled to 10% coverage. Filtering scores applied in each panel are indicated in brackets. The x-axis represents the cell types for which specific DMRs were defined, and the y-axis shows the reference cell types used to identify DMRs for each cell type on the x-axis.

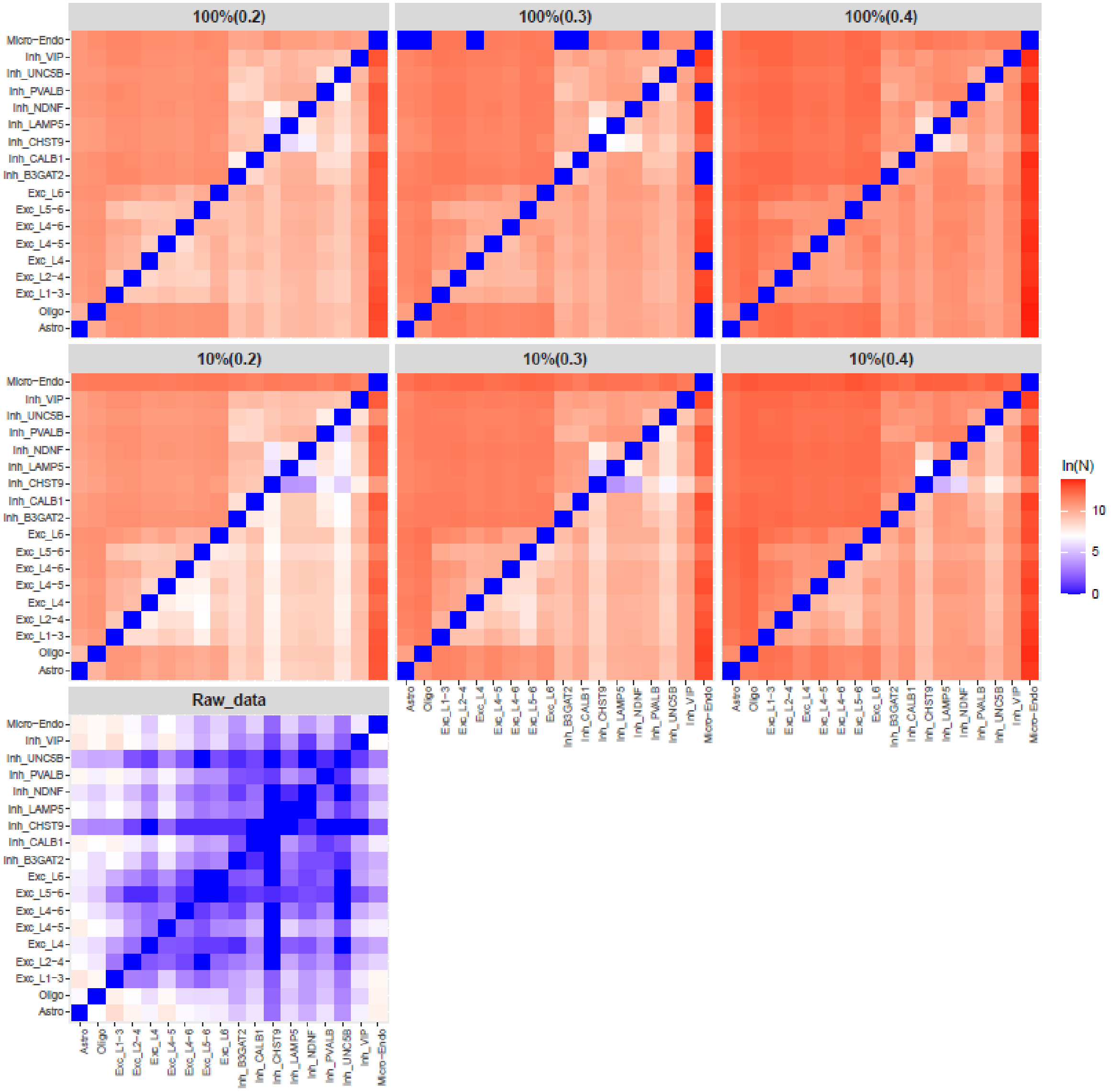

Figure S6. Number of cell type-specific DMRs (natural log scale) detected between each pair of cell types in the sn-m3C-seq dataset, Related to Figures 3-4 and Table S9. The upper panel displays DMR counts identified from imputed data based on the original, non-downsampled dataset; the middle panel shows DMR counts from imputed data based on data downsampled to 10% coverage; and the lower left panel shows DMR counts from raw data without imputation. Filtering scores for imputed data are indicated in brackets. The x-axis represents the cell types for which specific DMRs were defined, and the y-axis shows the reference cell types used to identify DMRs for each cell type on the x-axis.

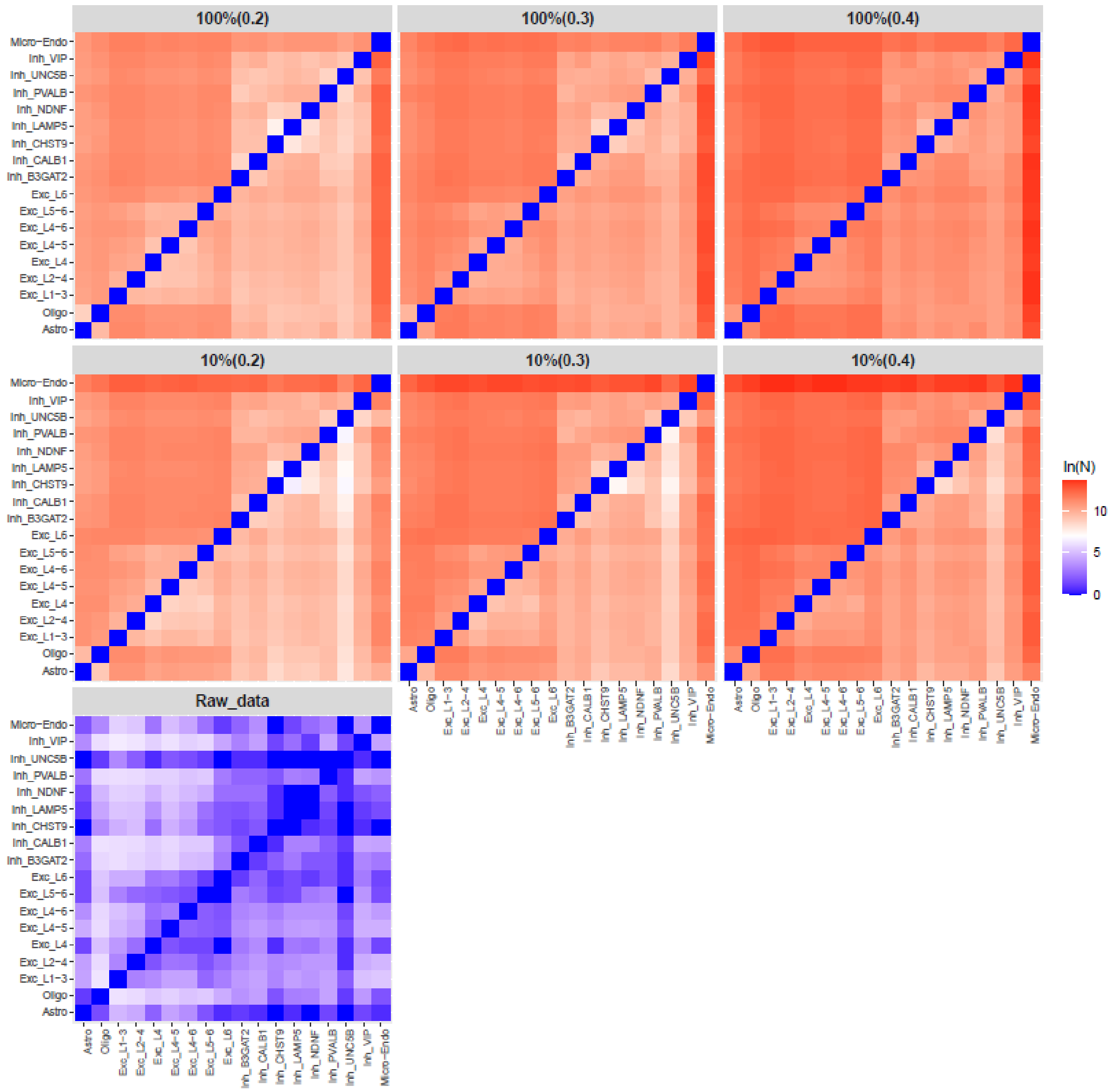

Figure S7. Number of cell type-specific DMRs (natural log scale) detected between each pair of cell types in the snmCAT-seq dataset, Related to Figures 3-4 and Table S9. The upper panel displays DMR counts identified from imputed data based on the original, non-downsampled dataset; the middle panel shows DMR counts from imputed data based on data downsampled to 10% coverage; and the lower left panel shows DMR counts from raw data without imputation. Filtering scores for imputed data are indicated in brackets. The x-axis represents the cell types for which specific DMRs were defined, and the y-axis shows the reference cell types used to identify DMRs for each cell type on the x-axis.

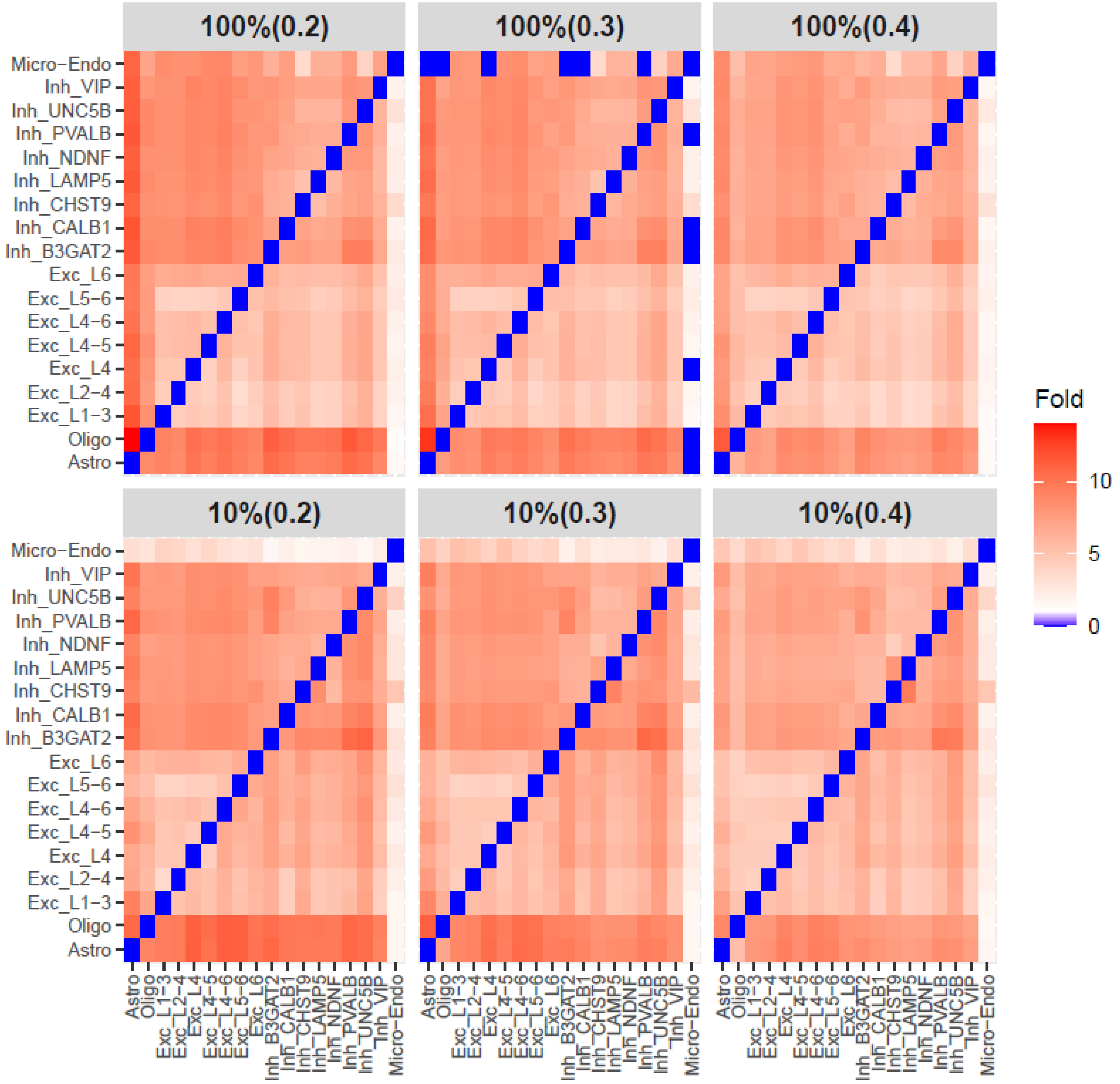

Figure S8. Enrichment fold for H3K27ac marks in the corresponding broad cell type for cell type-specific DMRs detected between each pair of cell types in the sn-m3C-seq dataset, Related to Figures 3-4 and Table S11. The upper panel displays enrichment fold for DMRs identified from imputed data based on the original, non-downsampled dataset, while the lower panel shows enrichment fold for DMRs identified from imputed data based on data downsampled to 10% coverage. Filtering scores for imputed data are indicated in brackets. The x-axis represents cell types for which specific DMRs were defined, and the y-axis shows the reference cell types used to identify DMRs for each cell type on the x-axis.

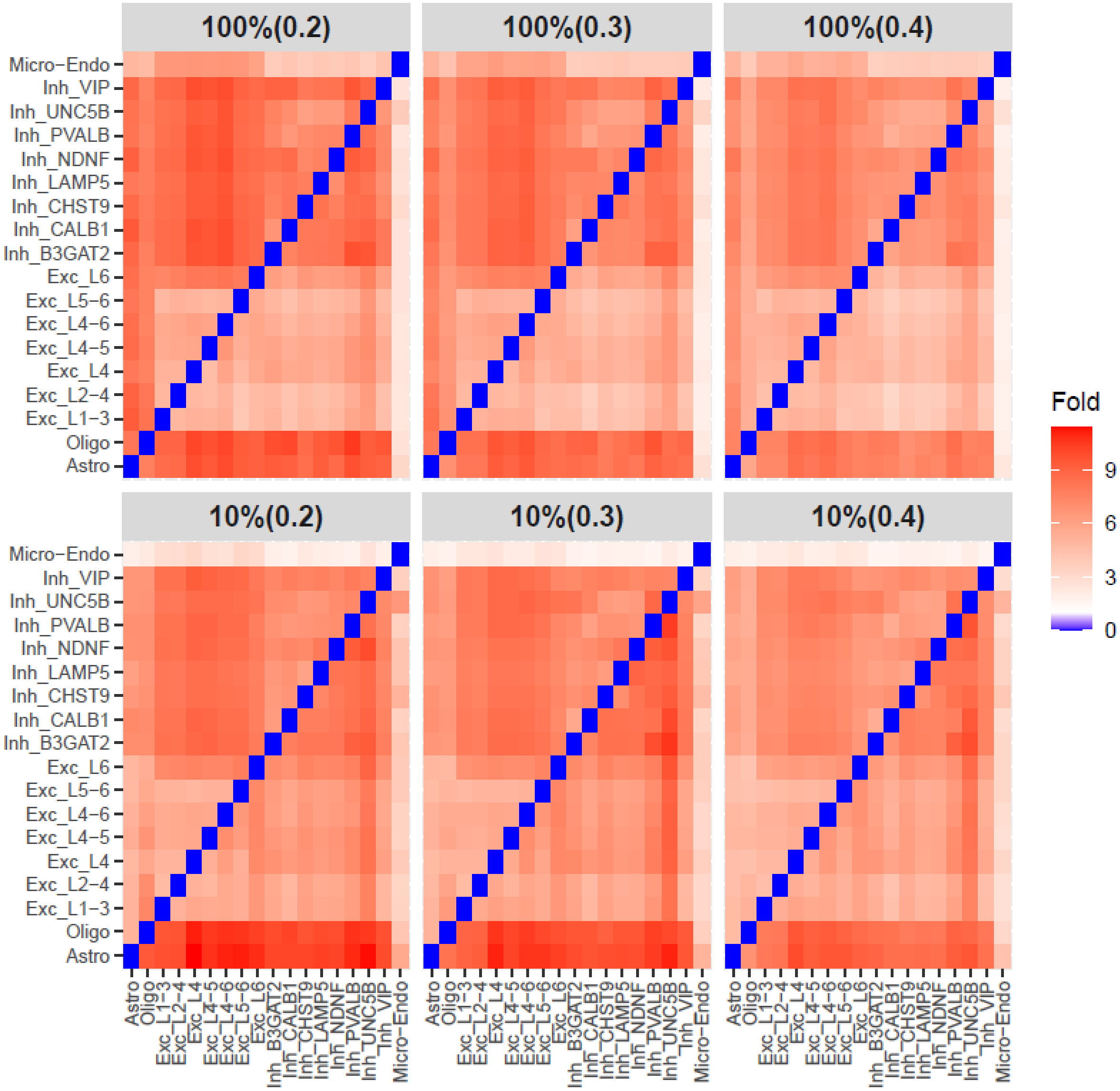

Figure S9. Enrichment fold for H3K27ac marks in the corresponding broad cell type for cell type-specific DMRs detected between each pair of cell types in the snmCAT-seq dataset, Related to Figures 3-4 and Table S11. The upper panel displays enrichment fold for DMRs identified from imputed data based on the original, non-downsampled dataset, while the lower panel shows enrichment fold for DMRs identified from imputed data based on data downsampled to 10% coverage. Filtering scores for imputed data are indicated in brackets. The x-axis represents cell types for which specific DMRs were defined, and the y-axis shows the reference cell types used to identify DMRs for each cell type on the x-axis.

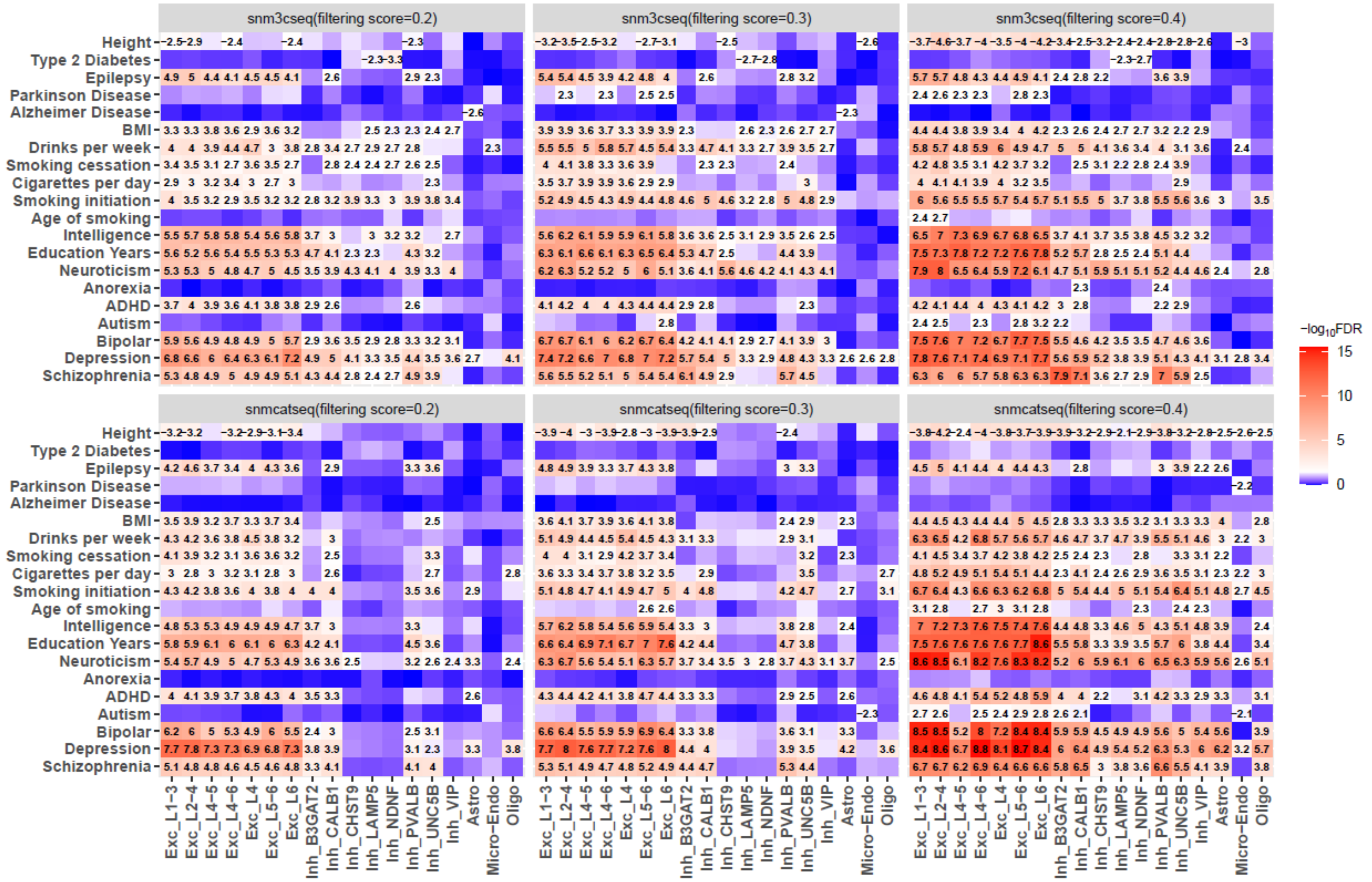

Figure S10. Heritability enrichment analysis for cell type-specific DMRs identified from imputed data of the sn-m3C-seq and snmCAT-seq datasets using three different filtering scores (0.2, 0.3, and 0.4), Related to Figure 6 and Table S12. The x-axis represents cell types for which DMRs were defined relative to all other cell types, while the y-axis lists various GWAS traits. The color scale represents the -$\log_{10}$(FDR) values derived from the z-score of per-SNP heritability calculated by stratified LDSC regression. White color denotes FDR = 0.05. Numbers within the squares show z-scores of per-SNP heritability that are significant after multiple testing correction (FDR < 0.05). Significant negative z-scores indicate a depletion of heritability.

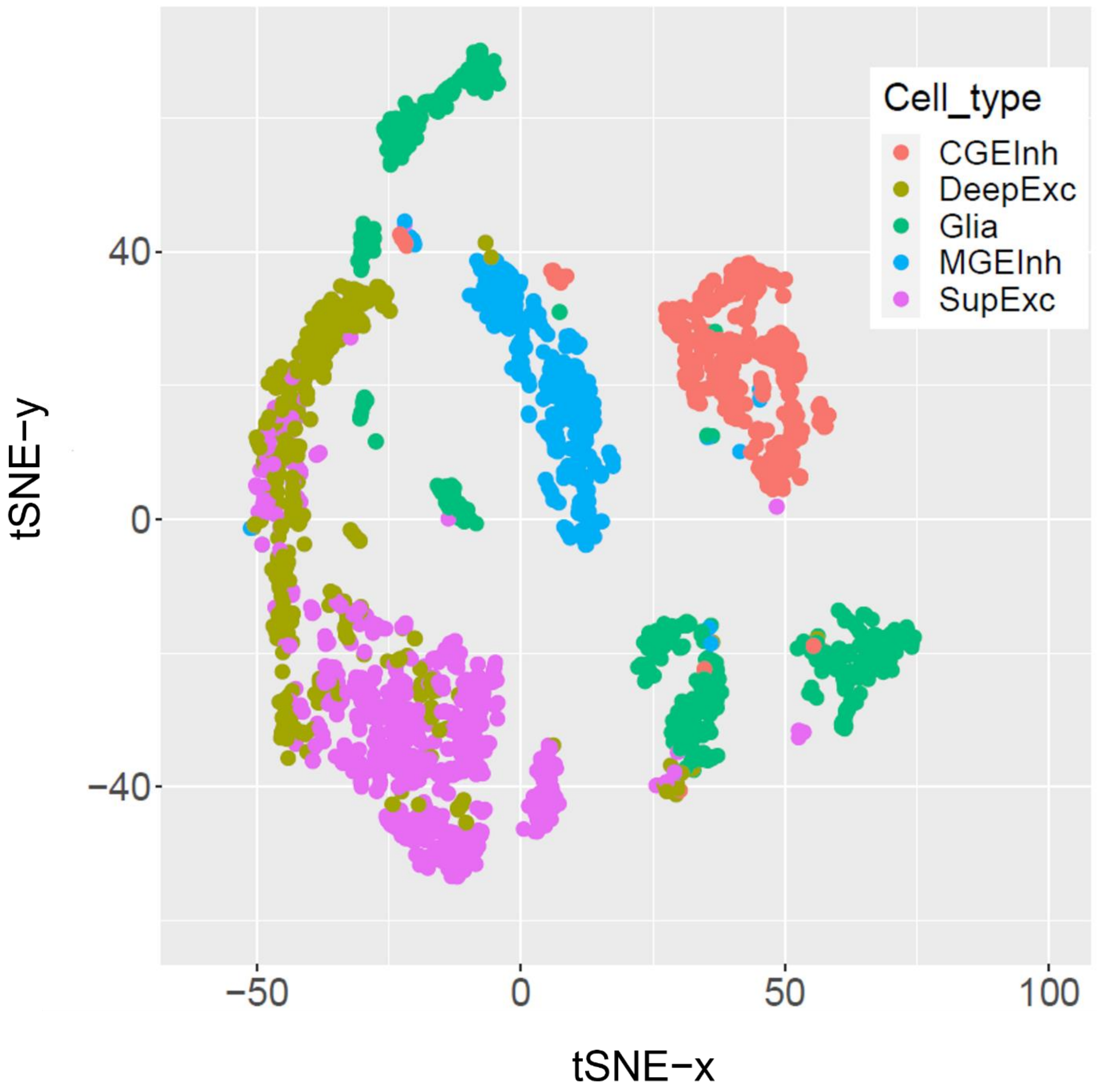

Figure S11. T-SNE plot using imputed CpG sites resemble clusters obtained using CpHs in the raw data, Related to Figure 7 and STAR Methods.

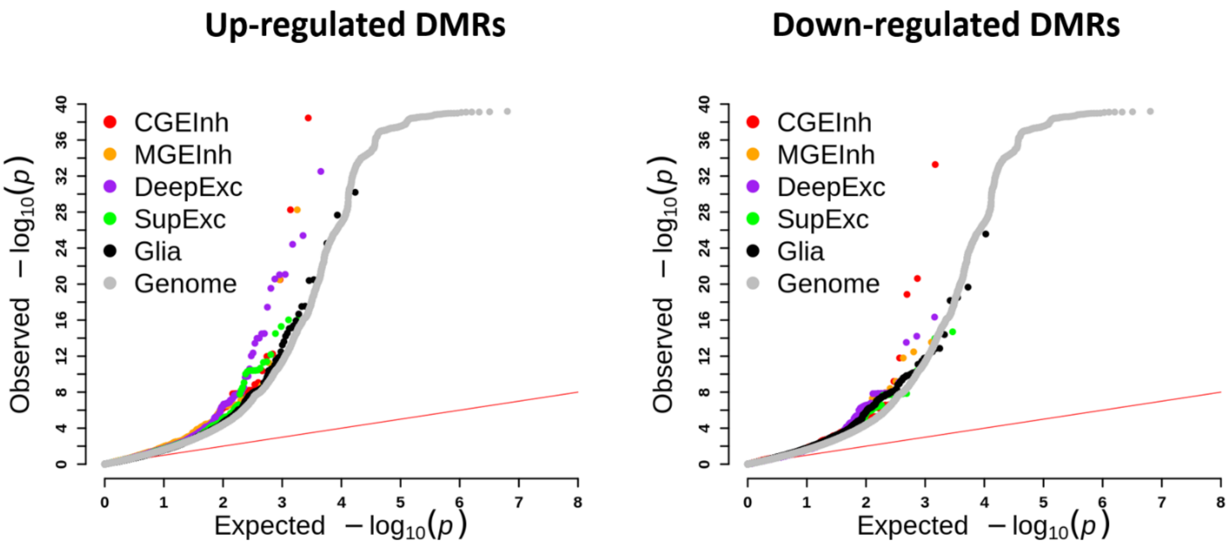

Figure S12. Quantile-Quantile (Q-Q) plot of SCZ GWAS p-values for SNPs located within or near up-regulated DMRs (left panel) and down-regulated DMRs (right panel) identified in each cell type, Related to Figure 7.

Table S1. Prediction performance of scMeFormer and alternative models across five single-nucleus DNAm datasets, Related to Figure 2. Performance was evaluated using all independent testing CpG sites on chromosome 22.

Method	Dataset	AUPRC
scMeFormer(finetune)	snmC-seq	0.906
scMeFormer(scratch)	snmC-seq	0.906
DNA module	snmC-seq	0.803
CpG module	snmC-seq	0.898
Cluster	snmC-seq	0.845
CNN	snmC-seq	0.839
scMeFormer(finetune)	snmC-seq2	0.888
scMeFormer(scratch)	snmC-seq2	0.886
DNA module	snmC-seq2	0.779
CpG module	snmC-seq2	0.878
Cluster	snmC-seq2	0.834
CNN	snmC-seq2	0.827
scMeFormer(finetune)	sn-m3C-seq	0.855
scMeFormer(scratch)	sn-m3C-seq	0.859
DNA module	sn-m3C-seq	0.746
CpG module	sn-m3C-seq	0.848
Cluster	sn-m3C-seq	0.795
CNN	sn-m3C-seq	0.794
scMeFormer(finetune)	snmCAT-seq	0.857
scMeFormer(scratch)	snmCAT-seq	0.858
DNA module	snmCAT-seq	0.719
CpG module	snmCAT-seq	0.839
Cluster	snmCAT-seq	0.773
CNN	snmCAT-seq	0.791
scMeFormer(finetune)	mouse	0.843
scMeFormer(scratch)	mouse	0.844
DNA module	mouse	0.788
CpG module	mouse	0.838
Cluster	mouse	0.731
CNN	mouse	0.792

Table S4. Prediction performance of scMeFormer and the cluster model across five datasets under reduced CpG coverage through downsampling, Related to Figure 3. Performance was assessed using all independent testing CpG sites on chromosome 22.

Proportion	Dataset	Method	AUPRC
100%	snmC-seq	scMeformer	0.90601
100%	snmC-seq	Cluster	0.845974
50%	snmC-seq	scMeformer	0.903674
50%	snmC-seq	Cluster	0.819333
25%	snmC-seq	scMeformer	0.901682
25%	snmC-seq	Cluster	0.825288
10%	snmC-seq	scMeformer	0.897843
10%	snmC-seq	Cluster	0.841087
5%	snmC-seq	scMeformer	0.893423
5%	snmC-seq	Cluster	0.844065
2%	snmC-seq	scMeformer	0.879157
2%	snmC-seq	Cluster	0.829555
1%	snmC-seq	scMeformer	0.867643
1%	snmC-seq	Cluster	0.806888
100%	snmC-seq2	scMeformer	0.888574
100%	snmC-seq2	Cluster	0.834183
50%	snmC-seq2	scMeformer	0.885001
50%	snmC-seq2	Cluster	0.790978
25%	snmC-seq2	scMeformer	0.882625
25%	snmC-seq2	Cluster	0.786746
10%	snmC-seq2	scMeformer	0.876656
10%	snmC-seq2	Cluster	0.799944
5%	snmC-seq2	scMeformer	0.87258
5%	snmC-seq2	Cluster	0.808064
2%	snmC-seq2	scMeformer	0.857521
2%	snmC-seq2	Cluster	0.785802
1%	snmC-seq2	scMeformer	0.847684
1%	snmC-seq2	Cluster	0.759909
100%	sn-m3C-seq	scMeformer	0.854636
100%	sn-m3C-seq	Cluster	0.794826
50%	sn-m3C-seq	scMeformer	0.847252
50%	sn-m3C-seq	Cluster	0.744647
25%	sn-m3C-seq	scMeformer	0.847505
25%	sn-m3C-seq	Cluster	0.740003
10%	sn-m3C-seq	scMeformer	0.839885
10%	sn-m3C-seq	Cluster	0.739125
5%	sn-m3C-seq	scMeformer	0.833364
5%	sn-m3C-seq	Cluster	0.740883

2%	sn-m3C-seq	scMeformer	0.816339
2%	sn-m3C-seq	Cluster	0.738501
1%	sn-m3C-seq	scMeformer	0.800184
1%	sn-m3C-seq	Cluster	0.717626
100%	snmCAT-seq	scMeformer	0.856788
100%	snmCAT-seq	Cluster	0.77363
50%	snmCAT-seq	scMeformer	0.851361
50%	snmCAT-seq	Cluster	0.733988
25%	snmCAT-seq	scMeformer	0.834607
25%	snmCAT-seq	Cluster	0.735762
10%	snmCAT-seq	scMeformer	0.825876
10%	snmCAT-seq	Cluster	0.739009
5%	snmCAT-seq	scMeformer	0.814037
5%	snmCAT-seq	Cluster	0.735743
2%	snmCAT-seq	scMeformer	0.799167
2%	snmCAT-seq	Cluster	0.721702
1%	snmCAT-seq	scMeformer	0.779305
1%	snmCAT-seq	Cluster	0.69699
100%	Mouse_embryo	scMeformer	0.843165
100%	Mouse_embryo	Cluster	0.731858
50%	Mouse_embryo	scMeformer	0.832881
50%	Mouse_embryo	Cluster	0.732428
25%	Mouse_embryo	scMeformer	0.830852
25%	Mouse_embryo	Cluster	0.728033
10%	Mouse_embryo	scMeformer	0.824373
10%	Mouse_embryo	Cluster	0.713479
5%	Mouse_embryo	scMeformer	0.820564
5%	Mouse_embryo	Cluster	0.700707
2%	Mouse_embryo	scMeformer	0.817195
2%	Mouse_embryo	Cluster	0.667145
1%	Mouse_embryo	scMeformer	0.809612
1%	Mouse_embryo	Cluster	0.658802

Table S10. Precision estimates for DMRs identified from imputed data using various filtering criteria, Related to Figure 4.

Filter	total DMR number	overlapped with ground truth	precision
0.2	407,120	326,700	0.802
0.3	443,676	335,611	0.756
0.4	806,234	562,838	0.698
no filter	1,729,786	956,042	0.553
**Total number of ground truth DMRs**	**1,163,204**		

Table S13. Numbers of SCZ-associated DMRs detected in each cell type, stratified by direction of effect, Related to Figure 7

CellType	Direction	Count
MGEInh	All	3468
MGEInh	Up	2288
MGEInh	Down	1180
CGEInh	All	3868
CGEInh	Up	2415
CGEInh	Down	1453
DeepExc	All	5808
DeepExc	Up	4586
DeepExc	Down	1222
Glia	All	19265
Glia	Up	11993
Glia	Down	7272
SupExc	All	6055
SupExc	Up	3963
SupExc	Down	2092

Table S14. MAGMA gene set-based association with SCZ for genes linked to DMRs in each cell type, Related to Figure 7. Default parameters were used using a one-sided positive test, conditional on gene size, gene density, sample size, inverse MAC, log(gene size), log(gene density), log(sample size), and log(inverse MAC)

Gene sets	CellType	DMR Direction	NGENES	BETA	BETA_STD	SE	P	FDR
CGEInh	CGEInh	All	563	0.13803	0.024603	0.052707	0.004416	0.013248
CGEInh_up	CGEInh	Up	328	0.12664	0.017351	0.06822	0.031709	0.06645
CGEInh_down	CGEInh	Down	235	0.14616	0.016996	0.080666	0.035011	0.06645
MGEInh	MGEInh	All	514	0.097976	0.01671	0.054238	0.03544	0.06645
MGEInh_up	MGEInh	Up	277	0.11232	0.014163	0.074343	0.065428	0.098142
MGEInh_down	MGEInh	Down	237	0.079106	0.009238	0.078072	0.15548	0.1794
DeepExc	DeepExc	All	774	0.13711	0.028471	0.044408	0.001011	0.007583
DeepExc_up	DeepExc	Up	598	0.13714	0.025165	0.050595	0.003363	0.013248
DeepExc_down	DeepExc	Down	176	0.12089	0.012187	0.087101	0.082594	0.112628
SupExc	SupExc	All	783	0.13992	0.029214	0.044946	0.000928	0.007583
SupExc_up	SupExc	Up	477	0.15213	0.025023	0.057805	0.004252	0.013248
SupExc_down	SupExc	Down	306	0.11346	0.015025	0.069177	0.050494	0.084157
Glia	Glia	All	1181	0.028283	0.007164	0.036326	0.21812	0.2337
Glia_up	Glia	Up	601	0.051093	0.009398	0.049264	0.14985	0.1794
Glia_down	Glia	Down	580	0.00103	0.000186	0.051902	0.49209	0.49209

Table S15. Enrichment of genes linked to DMRs for differentially expressed genes identified in a single-cell RNA-seq study of SCZ, Related to Figure 7.

Cell type	DMR direction	enrichment fold	standard error	p
CGEInh	Up	1.369	0.288	1.73E-01
CGEInh	Down	0.557	1.008	8.343-1
MGEInh	Up	1.912	0.346	5.67E-02
MGEInh	Down	0.476	1.007	8.77E-01
DeepExc	Up	2.065	0.117	7.26E-09
DeepExc	Down	1.434	0.511	3.13E-01
SupExc	Up	1.546	0.164	7.05E-03
SupExc	Down	1.236	0.364	3.31E-01
Glia	Up	1.294	0.375	2.99E-01
Glia	Down	1.162	0.318	3.64E-01

Table S18. Estimated training time (hours) required for each model over 10 epochs across varying numbers of cells, Related to STAR Methods

Number of cells	CpG-Transformer	DeepCpG	GraphCpG	scMeFormer
4000	5560	2380	30820	72
2000	2780	530	17220	70
1000	440	290	5830	70
500	250	140	2610	69
200	80	80	970	70
100	50	60	510	68

Table S19. hyperparameters used in scMeFormer, Related to STAR Methods

**Hyperparameters**	**DNA module**	**CpG module**
Number of layers in CNN module	3	3
Number of kernels in each CNN layer	256	256
Kernel size for each CNN layer	10	11
Number of max-pooling layers	1	NA
Kernel size for max-pooling layer	20	NA
Step size for max-pooling layer	20	NA
Dropout rate for dropout layer	0.2	0.2
Number of attention layers in Transformer module	8	8
Number of attention headers	8	8
Input size	256	256
Hidden size	256	256
Intermediate size	1024	1024
Output size	256	256
Dropout rate	0.1	0.1
Number hidden layer in fully connected network	1	
Input size for fully connected network	768	
Hidden size for fully connected network	768	
Number of tasks (output size for fully connected	The number of cells	
Initial learning rate	0.000176	
Number of learning rate warmup steps	10000	
