# Supplementary material for: Deep learning imputes DNA methylation states in single cells and enhances the detection of epigenetic alterations in schizophrenia
Source: Cell Genom. 2025 Feb 21;5(3):100774. doi: 10.1016/j.xgen.2025.100774 (PMC11960545; doi:10.1016/j.xgen.2025.100774)
Supplement: Document S2. Article plus supplemental information [file mmc14.pdf]

Cell Genomics

Article

Deep learning imputes DNA methylation states in single cells and enhances the detection of epigenetic alterations in schizophrenia

Graphical abstract

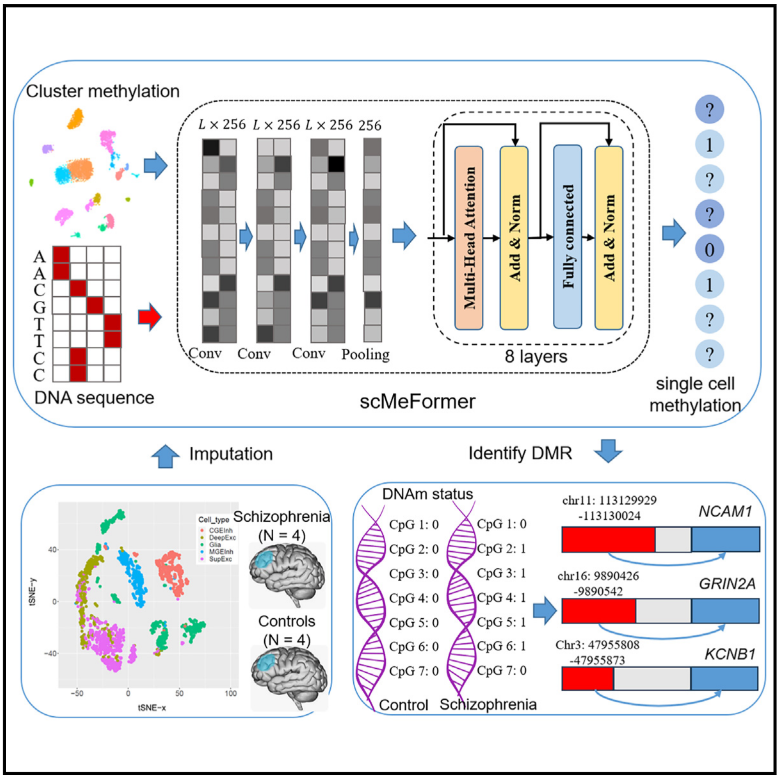

Authors

Jiyun Zhou, Chongyuan Luo, Hanqing Liu, ..., Joseph R. Ecker, Daniel R. Weinberger, Shizhong Han

Correspondence

shizhong.han@libd.org

In brief

Zhou et al. developed scMeFormer, a deep learning model for imputing single-cell DNA methylation datasets. It outperforms existing methods in accuracy and efficiency. Applying scMeFormer to single-nucleus DNAm data from the prefrontal cortex of patients with schizophrenia and controls uncovered thousands of schizophrenia-associated differentially methylated regions that were undetectable without imputation.

Highlights

- scMeFormer is a deep learning model for single-cell DNA methylation data imputation
- Outperforms state-of-the-art methods in accuracy and efficiency
- Includes a filtering metric for quality control of imputed data
- Identifies thousands of schizophrenia-associated DMRs undetectable without imputation

Zhou et al., 2025, Cell Genomics 5, 100774
March 12, 2025 © 2025 The Author(s). Published by Elsevier Inc.
https://doi.org/10.1016/j.xgen.2025.100774

CellPress

Cell Genomics

CellPress
OPEN ACCESS

Article

Deep learning imputes DNA methylation states in single cells and enhances the detection of epigenetic alterations in schizophrenia

Jiyun Zhou,[1] Chongyuan Luo,[2] Hanqing Liu,[3] Matthew G. Heffel,[2,4] Richard E. Straub,[1] Joel E. Kleinman,[1,6] Thomas M. Hyde,[1,6,9] Joseph R. Ecker,[3,5] Daniel R. Weinberger,[1,6,7,8,9] and Shizhong Han[1,6,7,10,*]

[1]Lieber Institute for Brain Development, Johns Hopkins Medical Campus, Baltimore, MD 21287, USA
[2]Department of Human Genetics, University of California, Los Angeles, Los Angeles, CA 90095, USA
[3]Genomic Analysis Laboratory, The Salk Institute for Biological Studies, La Jolla, CA 92037, USA
[4]Bioinformatics Interdepartmental Program, University of California, Los Angeles, Los Angeles, CA 90095, USA
[5]Howard Hughes Medical Institute, The Salk Institute for Biological Studies, La Jolla, CA 92037, USA
[6]Department of Psychiatry and Behavioral Sciences, Johns Hopkins University School of Medicine, Baltimore, MD 21287, USA
[7]Department of Genetic Medicine, Johns Hopkins University School of Medicine, Baltimore, MD 21205, USA
[8]Department of Neuroscience, Johns Hopkins University School of Medicine, Baltimore, MD 21205, USA
[9]Department of Neurology, Johns Hopkins University School of Medicine, Baltimore, MD 21205, USA
[10]Lead contact
*Correspondence: shizhong.han@libd.org
https://doi.org/10.1016/j.xgen.2025.100774

SUMMARY

DNA methylation (DNAm) is a key epigenetic mark with essential roles in gene regulation, mammalian development, and human diseases. Single-cell technologies enable profiling DNAm at cytosines in individual cells, but they often suffer from low coverage for CpG sites. We introduce scMeFormer, a transformer-based deep learning model for imputing DNAm states at each CpG site in single cells. Comprehensive evaluations across five single-nucleus DNAm datasets from human and mouse demonstrate scMeFormer's superior performance over alternative models, achieving high-fidelity imputation even with coverage reduced to 10% of original CpG sites. Applying scMeFormer to a single-nucleus DNAm dataset from the prefrontal cortex of patients with schizophrenia and controls identified thousands of schizophrenia-associated differentially methylated regions that would have remained undetectable without imputation and added granularity to our understanding of epigenetic alterations in schizophrenia. We anticipate that scMeFormer will be a valuable tool for advancing single-cell DNAm studies.

INTRODUCTION

DNA methylation (DNAm) is a fundamental epigenetic mechanism that involves the addition of a methyl group to cytosines and plays a crucial role in gene regulation, mammalian development, and various human diseases.[1] Single-cell technologies enable the profiling of DNAm states at cytosines within the DNA sequence of individual cells, which complements single-cell transcriptome studies in understanding cellular heterogeneity, developmental processes, and disease states.[2,3] However, due to the limited DNA material available from individual cells and inherent technical limitations, current technologies often suffer from sparse coverage for CpG sites, typically measuring less than 10% of CpG sites in a single cell.[4–7] This limits their full potential to uncover the epigenetic landscape at single-cell resolution.

In order to address the challenge of sparse coverage in the single-cell DNAm dataset, computational methods have been developed for imputing DNAm states for CpG sites in individual cells. One such method is Melissa, a Bayesian model that draws inferences solely from DNAm profiles,[8] but it was designed for genomic regions of interest rather than genome-wide imputation. LightCpG is a traditional machine learning model that uses a light gradient boosting machine (LightGBM) to build prediction models with handcrafted features derived from CpG sites and DNA sequences to impute missing methylation states.[9] Similarly, CaMelia employs a CatBoost gradient boosting algorithm to predict methylation states based on the local similarity of methylation patterns across cells.[10] EpiScanpy,[11] an extension of the Scanpy toolkit[12] tailored for single-cell epigenomic data analysis, imputes DNAm levels in genomic intervals by replacing missing values with the average methylation across all cells for those intervals.

In recent years, deep-learning-based models have emerged for imputing single-cell DNAm data, automatically extracting features from DNAm profiles and DNA sequences. The first such model, DeepCpG,[13] is based on a traditional recurrent neural network. While DeepCpG learns features from both DNAm profiles and DNA sequences, it is limited by its ability to capture long-range dependencies and is computationally intensive. Another deep

This is an open access article under the CC BY license (http://creativecommons.org/licenses/by/4.0/).

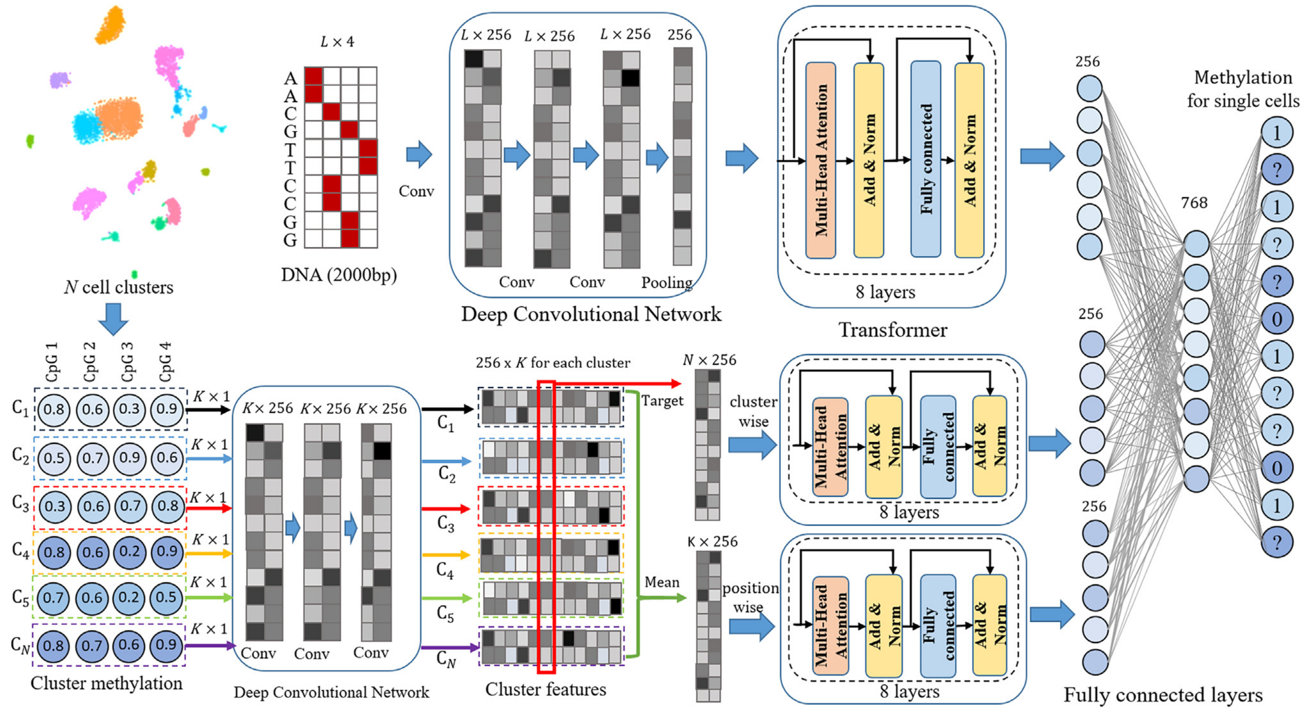

Figure 1. Illustration of scMeFormer architecture

scMeFormer consists of two main modules: the DNA module and the CpG module. The DNA module includes a three-layer CNN followed by an eight-layer transformer. It takes DNA sequences of dimension L × 4 (where L is the sequence length, equal to 2,000 bp in the real model) as input. The CNN extracts local features from the DNA sequences, which are then passed to the transformer to learn potential interactions among local DNA features. Similarly, the CpG module comprises a three-layer CNN and two eight-layer transformers. The input to the CNN is the DNAm levels of K CpG sites (K set to 100 in the real model) within each pre-defined cell cluster (C_1, C_2, …, C_N). The CNN identifies local DNAm patterns of CpG sites within each cluster through a one-dimensional convolutional kernel (256 kernels), generating a feature matrix of dimensions 256 × K for each cluster. The mean feature matrix across clusters is then fed into a position-wise transformer to capture relationships among positions. The feature matrix (red box) at the position of the target CpG is passed to the cluster-wise transformer to capture DNAm relationships among clusters. The output feature vectors from the DNA and CpG modules are catenated and passed into a fully connected network for prediction.

learning model, CpG Transformer,[14] builds upon the transformer model initially designed for language processing, which has the key advantage of capturing long-range dependency through an attention mechanism. CpG Transformer demonstrated superior performance compared to DeepCpG, but both models exhibit limitations in scalability that can only process up to several hundred cells, making them impractical for datasets involving thousands of cells—a scenario increasingly prevalent in the field.[4–7] The most recent model, GraphCpG,[15] employed a graph-based deep learning design and achieved state-of-the-art performance. However, GraphCpG only utilized the DNAm profile and did not consider DNA sequence features. Moreover, due to the single prediction task design of GraphCpG, this model can predict only one CpG in one cell at a time, making it challenging to scale to thousands of cells.

Here, we introduce scMeFormer, a transformer-based deep learning model, to efficiently impute DNAm states for each CpG site across thousands of single cells within a multi-task prediction framework, leveraging information from both local DNA sequences and DNAm profiles across cells. We demonstrate the superior performance of scMeFormer compared to alternative models across five single-nucleus DNAm datasets from the human brain and mouse embryo,[4–7,16] achieving high-fidelity imputation even with coverage reduced to 10% of original CpG sites. We further applied scMeFormer to a single-nucleus DNAm dataset we generated from the prefrontal cortex of four patients with schizophrenia (SCZ) and neurotypical controls. This enabled the identification of thousands of differentially methylated regions (DMRs) associated with SCZ that would have remained undetectable without imputation and added granularity to our understanding of epigenetic alterations in SCZ within specific cell types.

RESULTS

scMeFormer predicts DNAm states of CpG sites in single cells

The scMeFormer model architecture comprises two main modules: a DNA module and a CpG module (Figure 1). The DNA module is designed to learn DNAm motifs, while the CpG module aims to leverage DNAm information from neighboring CpG sites within and across pre-defined cell clusters. The DNA module contains a convolutional neural network (CNN) block for extracting local DNA features, followed by eight layers of transformer

CellPress
OPEN ACCESS

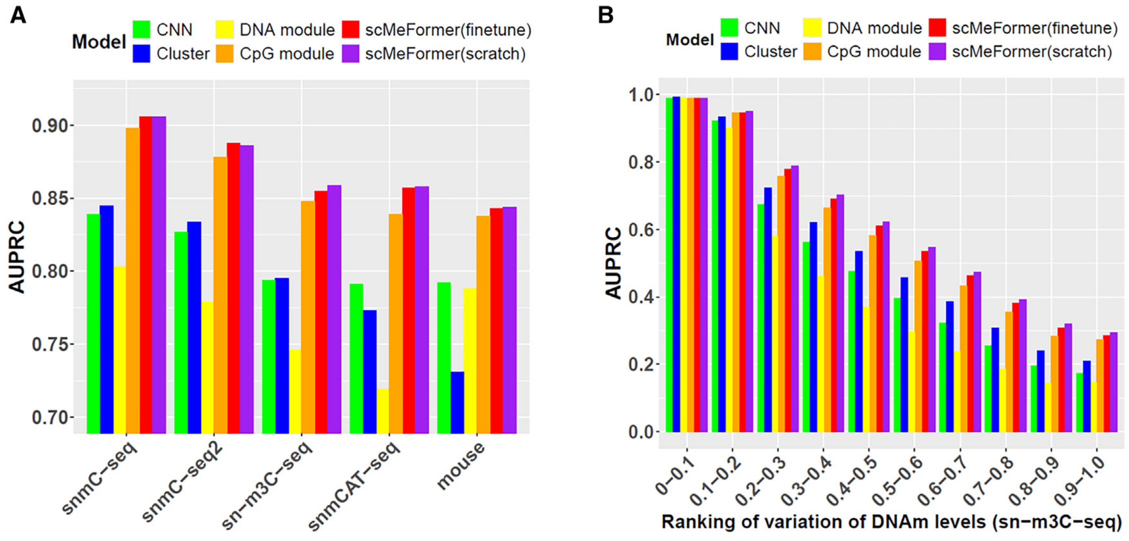

Figure 2. Comparison of model prediction performance between scMeFormer and four alternative models across five single-nucleus DNAm datasets

(A) Comparison was based on all independent testing CpG sites on chromosome 22.

(B) Comparison was based on subsets of independent testing CpG sites, stratified by their levels of variations across all cells in the sn-m3C-seq dataset. The "0–0.1" group represents the bottom 10% least-variable CpG sites. The "0.9–1.0" group represents the top 10% most-variable CpG sites.

See also Figure S1 and Table S2.

blocks to detect distant features that may cooperate to influence DNAm. The CpG module consists of a CNN block for extracting local patterns from neighboring CpG sites within each cell cluster, followed by two separate transformers to capture DNAm relationships among CpG sites and cell clusters. The features learned from both modules are catenated within a fully connected network to predict DNAm states for a given CpG site in a subset of cells whose DNAm states are measured. The model is trained by minimizing the prediction error, with only measured CpG sites contributing to the loss function in each training step.

We applied scMeFormer to five distinct single-nucleus DNAm datasets: four human brain datasets generated with unique technologies (snmC-seq,[4] snmC-seq2,[6] sn-m3C-seq,[5] and snmCAT-seq[7]) and one mouse embryo dataset generated by scNMT-seq.[16] These datasets ranged in size from 1,105 (scNMT-seq) to 4,357 (snmCAT-seq) nuclei. Model prediction performance was evaluated on each dataset using independent CpG sites not included in either model training or validation. When trained from scratch on each dataset (scMeFormer(scratch)), scMeFormer achieved remarkable prediction performance, with an average area under the precision-recall curve (AUPRC) of 0.871 across five datasets (Figure 2A; Table S1). To improve computational efficiency, we also implemented a pre-training and fine-tuning strategy, in which scMeFormer was initially pre-trained on one dataset and then fine-tuned on independent datasets. Remarkably, the fine-tuned model (scMeFormer(fine-tune)) performed comparably to the model trained from scratch across datasets, achieving an average AUPRC of 0.869. This strategy enables scMeFormer to be pre-trained once and then adapted to new datasets without retraining from scratch, significantly reducing computational costs. Notably, this strategy proved effective even across species: we pre-trained the model on the human sn-m3C-seq dataset and fine-tuned it on the mouse embryo dataset, achieving nearly the same performance as the model trained directly on the mouse dataset from scratch (fine-tune: AUPRC = 0.843; scratch: AUPRC = 0.844).

We further explored the contribution of each module to the overall performance of scMeFormer. A reduction in prediction performance occurred when scMeFormer employed only the CpG module (average AUPRC = 0.860) or the DNA module (average AUPRC = 0.767), indicating that both modules contribute complementary information for predicting DNAm states. Additionally, we compared scMeFormer with two alternative models: a CNN model without transformer blocks and a cluster-based model that imputes CpG states based on the mean DNAm levels of CpG sites in cells of the same cluster. scMeFormer consistently outperformed the two alternative models across the five datasets (Figure 2A).

We further evaluated the prediction performance of various models on subsets of independent CpG sites, stratified by their levels of variations across cells (Figures 2B and S1; Table S2). All models performed well for CpG sites with the least variability. For example, for CpGs ranked in the bottom 10% in terms of their variations, all models achieved an AUPRC > 0.97 across the five datasets. Notably, we observed a reduction in prediction performance for more variable CpG sites across all models and datasets. Nonetheless, scMeFormer consistently outperformed alternative models. For example, within the subset of the most variable CpG sites (ranked in the top 10%), scMeFormer(fine-tune) achieved an average AUPRC of 0.219 across the five datasets, which was 0.083 and 0.039 higher than the CNN and cluster models, respectively. For CpG sites with intermediate variability (ranked between the top 40% and top 50%),

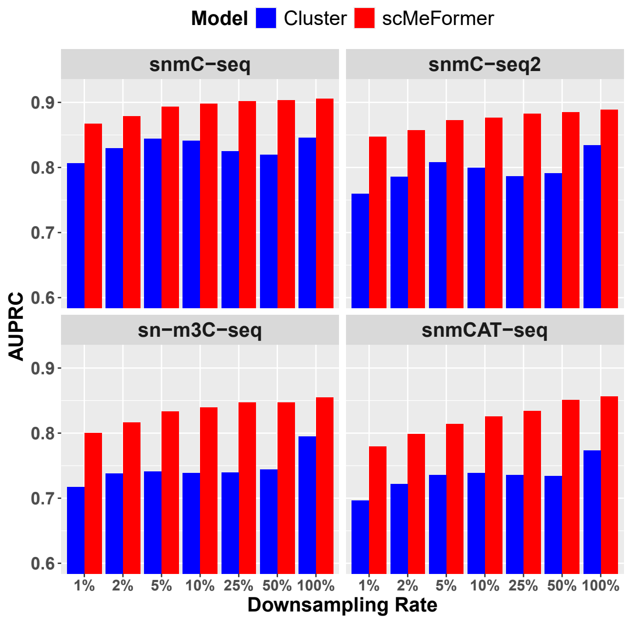

Figure 3. Comparison of model prediction performance between scMeFormer and the cluster model across the four human datasets under reduced CpG coverage through downsampling

Comparison was based on independent testing CpG sites on chromosome 22 in each dataset. As a comparison, we included prediction performance from raw data without downsampling as indicated by downsampling rate = 1 on the x axis. See also Table S4.

scMeFormer achieved an average AUPRC of 0.459, which was 0.144 higher than the CNN and 0.092 higher than the cluster model across the datasets.

To assess the genomic regions where imputation errors are more likely to occur, we evaluated the model prediction performance on CpG sites stratified by their chromatin states defined in the dorsolateral prefrontal cortex derived from the ROADMAP Epigenomics Project (Figure S2; Table S3). We observed that scMeFormer performed well (AURPC > 0.8) for CpG sites within five active states (TssA, TssAFlnk, TxFlnk, Enh, and EnhG), three poised states (TssBiv, BivFlnk, and EnhBiv), and the repressed Polycomb state (ReprPC). However, its performance was relatively lower for CpG sites in actively transcribed states (Tx and TxWk), regions associated with zinc-finger protein genes (ZNF/Rpts), heterochromatin (Het), weakly repressed Polycomb states (ReprPCWk), and quiescent regions (Quies).

We compared scMeFormer with three previous deep learning models (GraphCpG, CpG Transformer, and DeepCpG) and CaMelia, a gradient-boosting-based model. Due to the limitation of scalability of these models, we trained them on a randomly selected subset of 118 cells from the sn-m3C-seq dataset. Overall, scMeFormer achieved the highest performance (AUPRC = 0.869), followed by CpG Transformer (AUPRC = 0.868), DeepCpG (AUPRC = 0.816), CaMelia (AUPRC = 0.809), and GraphCpG (AUPRC = 0.735). Moreover, scMeFormer consistently outperformed these four models (except for CpG Transformer) when evaluated on subsets of CpG sites stratified by their levels of variation across cells (Figure S3).

scMeFormer performance under lower CpG coverage through downsampling

We assessed the performance of scMeFormer under conditions of lower CpG coverage in single cells through downsampling. Due to the significant reduction in training time, all of the following results from scMeFormer refer to fine-tuned models unless otherwise specified. We systemically reduced the number of CpG sites used for training the model from 50% to 1% to mimic scenarios with lower coverage. In this investigation, we compared scMeFormer with only the cluster model since the cluster model performed generally better than the CNN in datasets without downsampling. We observed a trend of slightly reduced prediction performance at lower CpG coverage for both models, but scMeFormer consistently outperformed the clustering model across all five datasets (Table S4). Figure 3 illustrates the comparative results across the four human datasets. Notably, both models achieved good performance even at 1% of the original CpG coverage (scMeFormer, average AUPRC = 0.821; cluster, average AUPRC = 0.728). Additionally, we evaluated the two models under downsampling situations for subsets of independent CpG sites, stratified by their levels of variations across cells (Figure S4; Table S5). Consistent with our observations in non-downsampled datasets, a reduction in prediction performance occurred for more variable CpG sites for both models. However, scMeFormer demonstrated superior performance compared to the cluster model across all five datasets, particularly for the more variable CpG sites.

An imputation quality metric enhances imputation performance

To evaluate the quality of imputed DNAm states for each CpG site in each cell, we defined an imputation quality score metric, which measures the average absolute difference of predicted DNAm states between the target CpG site and its five upstream and five downstream neighboring CpG sites. We reasoned that lower score values indicate more reliable imputed states for the target CpG sites, as methylation states tends to be similar among nearby CpG sites. In contrast, higher score values may reflect poorer imputations that resemble random guesses. We applied various score thresholds as filters, retaining only CpG sites with scores below the threshold. Remarkably, we observed a clear trend of improved prediction performance with more stringent filtering (Figure 4; Table S6), supporting the reliability of this filtering metric. For instance, in the sn-m3C-seq dataset without downsampling, the AUPRC increased from 0.855 without filtering to 0.935 when a filtering score threshold of 0.1 was applied. This trend was consistently observed across datasets downsampled at different rates.

scMeFormer recovers cell clusters under low coverage through downsampling

To further assess the fidelity of imputed data under reduced CpG coverage achieved by downsampling, we examined how well imputed CpG sites could recover cell type clusters originally identified from the raw data across the four human datasets. We used the adjusted Rand index (ARI) to measure the similarity between clusters obtained from the original data and those derived from (1) downsampled data without imputation, (2)

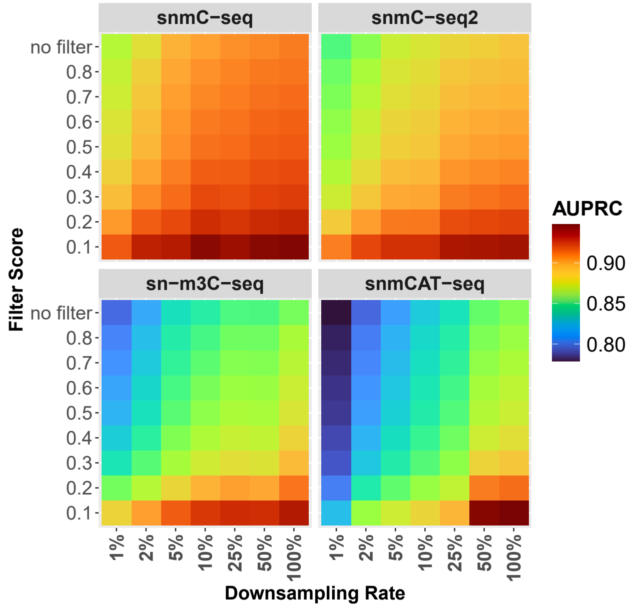

Figure 4. Comparison of scMeFormer model prediction performance at different filtering score thresholds across four human datasets, evaluated under various levels of reduced CpG coverage achieved through downsampling

The x axis represents various downsampling rates, and the y axis represents the applied filtering score thresholds, including a no-filtering condition. Comparison was based on independent testing CpG sites on chromosome 22 in each dataset. See also Table S6.

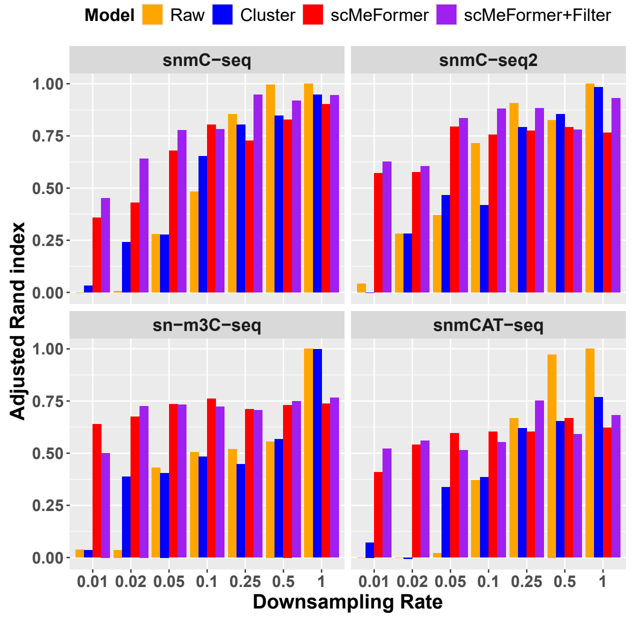

Figure 5. Evaluation of imputed data under lower CpG coverage in its ability to recover cell types identified in the original data across four human datasets

The x axis represents various downsampling rates, and the y axis represents the adjusted Rand index. The orange bar (raw) represents the downsampled data without imputation. See also Table S7.

downsampled data imputed by the cluster model, and (3) downsampled data imputed by scMeFormer without or with a filtering (score = 0.2). Overall, scMeFormer with filtering achieved superior performance (average ARI = 0.72) compared to scMeFormer without filtering (average ARI = 0.67) across all datasets and downsampling rates (Table S7). scMeFormer maintained robust performance (ARI > 0.6) across all four datasets, even at a downsampling rate as low as 0.05, but this was not the case for the cluster model or the method without imputation (Figure 5; Table S7). Specifically, scMeFormer achieved average ARIs of 0.70 (without filtering) and 0.71 (with filtering), whereas the other two methods yielded much lower ARIs (without imputation, ARI = 0.28; cluster model, ARI = 0.37). At even lower downsampling rates (<0.05), the performance of the other two methods substantially declined, whereas scMeFormer exhibited a slower rate of decay, remaining comparable to its performance at a downsampling rate of 0.05.

scMeFormer boosts the detection of cell-type-specific DMRs

To further evaluate the quality of imputed data, we assessed its ability to recover DMRs between cell types identified in the original data for two datasets (sn-m3C-seq and snmCAT-seq). We defined cell-type-specific DMRs as regions containing at least two differentially methylated CpG sites (DMSs), with each DMS exhibiting lower DNAm levels in the specific cell type, since hypo-DMRs are strong indicators of regulatory elements.[17–19] We applied three filtering scores (0.2, 0.3, and 0.4) on the imputed datasets. As expected, we observed a trend of higher recall rates with less stringent filtering in both the raw and the 10% downsampled data (Figure S5; Table S8). For instance, with a filtering score of 0.2, scMeFormer achieved average recall rates of 0.45 in the sn-m3C-seq dataset and 0.47 in the snmCAT-seq dataset for cell-type-specific DMRs across all cell type pairs. When the filtering score increased to 0.4, the recall rates rose to 0.69 in both datasets. Notably, even on 10% downsampled data, scMeFormer maintained slightly lower yet comparable recall rates across both datasets. These results demonstrate the efficacy of scMeFormer in imputing CpG sites that preserve information crucial for identifying cell-type-specific DMRs.

We compared the number of cell-type-specific DMRs detected from imputed data with that from raw data. In the raw, unimputed datasets, we identified an average of only 304 DMRs across all cell type pairs in the sn-m3C-seq dataset and 71 DMRs in the snmCAT-seq dataset. In contrast, imputed datasets with various filtering thresholds (0.2, 0.3, and 0.4) yielded tens to hundreds of thousands of DMRs. As expected, we observed a trend of higher DMR counts with less stringent filtering in both raw and 10% downsampled data (Figures S6 and S7; Table S9). For instance, with a filtering score of 0.2, averages of 45,185 and 43,684 DMRs were identified across all cell type pairs in the sn-m3C-seq and snmCAT-seq datasets, respectively, and these numbers increased to 128,287 and 135,970 with a filtering score of 0.4. Notably, comparable DMR counts were observed when scMeFormer was applied to the 10% downsampled data, demonstrating scMeFormer's robust ability to detect cell-type-specific DMRs even under reduced coverage.

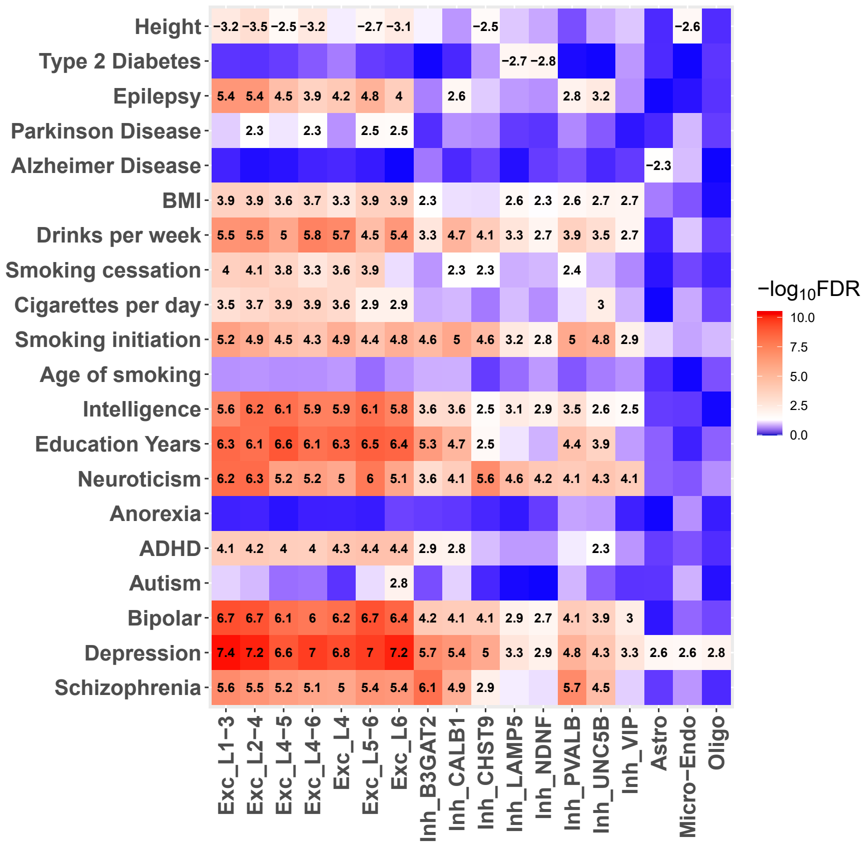

Figure 6. Heritability enrichment analysis for cell-type-specific DMRs identified from imputed sn-m3C-seq dataset

The x axis represents cell types for which DMRs were defined relative to all other cell types, while the y axis lists various GWAS traits. The color scale represents the $-\log_{10}$(FDR) values derived from the Z score of per-SNP heritability calculated by stratified linkage disequilibrium score regression (LDSC). The white color denotes FDR = 0.05. Numbers within the squares show Z scores of per-SNP heritability that are significant after multiple testing correction (FDR < 0.05). Significant negative Z scores indicate a depletion of heritability. See also Figure S10 and Table S12.

To estimate the false positive rates of our model in calling cell-type-specific DMRs, we turned to two broad cell types (neuron and glia) in the sn-m3C-seq dataset that allows us to identify CpG sites of sufficient coverage for identifying DMSs in the raw data. Specifically, we first collected CpG sites not within blacklist regions[20] but with high coverage ($\geq$100 reads) in each broad cell type to derive ground-truth labels for DMRs. In total, we detected 1,163,204 DMRs between two broad cell types, which were considered true positives. We then performed DMR analysis between the two cell types using imputed data, both without filtering and with filtering, at three score thresholds (0.2, 0.3, and 0.4). Remarkably, we observed higher precision rates with more stringent filtering, achieving a precision of 0.8 at a filtering score of 0.2 (Table S10). In contrast, the precision from imputed data without filtering was only 0.55. These results highlight the utility of filtering scores in enhancing the precision of DMR detection.

To assess the biological relevance of cell-type-specific DMRs identified from imputed data, we leveraged histone mark (H3K27ac) data that indicate active enhancers in four broad cell types (neuron, astrocyte, oligo, and microglia).[21] Given prior evidence that regulatory regions are associated with low DNAm levels, we hypothesized that reliable cell-type-specific DMRs would be enriched for H3K27ac marks in the corresponding broad cell type. Our analysis confirmed this hypothesis, revealing that cell-type-specific DMRs were enriched for H3K27ac-marked regions in the corresponding broad cell type across both datasets (Figures S8 and S9; Table S11). Notably, this enrichment pattern remained consistent even with scMeFormer applied to the 10% downsampled data. This enrichment pattern became stronger when DMRs were identified from imputed data with a more stringent filtering score, reassuring the reliability of the filtering metric.

To further validate the biological relevance of cell-type-specific DMRs identified from imputed data, we examined their enrichment for the heritability of 18 brain-related traits and two non-brain traits, human height and type 2 diabetes. The rationale for this analysis was that cell-type-specific DMRs enriched for regulatory regions in particular cell types should also enrich the heritability for traits related to those cell types. This was indeed observed for DMRs detected in both sn-m3C-seq and snmCAT-seq datasets (Figure S10; Table S12). Figure 6 shows the heritability enrichment analysis results from the imputed sn-m3C-seq dataset with a filtering score of 0.3, showing substantial enrichment for most brain-related traits and disorders, but not for height or diabetes, in DMRs specific to neuronal cell types, especially excitatory subtypes. In contrast, weak to no enrichment was observed for DMRs specific to non-neuronal cell types across all brain disorders except for depression, where we noted enrichment for DMRs specific to astrocytes, oligodendrocytes, and microglia or endothelial cell types, supporting the emerging role of glial cells in depression.[22–24] This pattern remained consistent in the snmCAT-seq dataset and was robust across different filtering scores (0.2 and 0.4) for imputed data (Figure S10).

scMeFormer enhances the detection of SCZ-associated DMRs

We applied scMeFormer to impute CpG states in 2,534 single-nucleus DNAm profiles generated from the prefrontal cortex of four patients with SCZ and four neurotypical controls. We first evaluated whether imputed data could recover clusters derived from CpHs, which is better suited for clustering neuronal cell types than CpGs.[4] Specifically, using DNAm levels of CpHs in

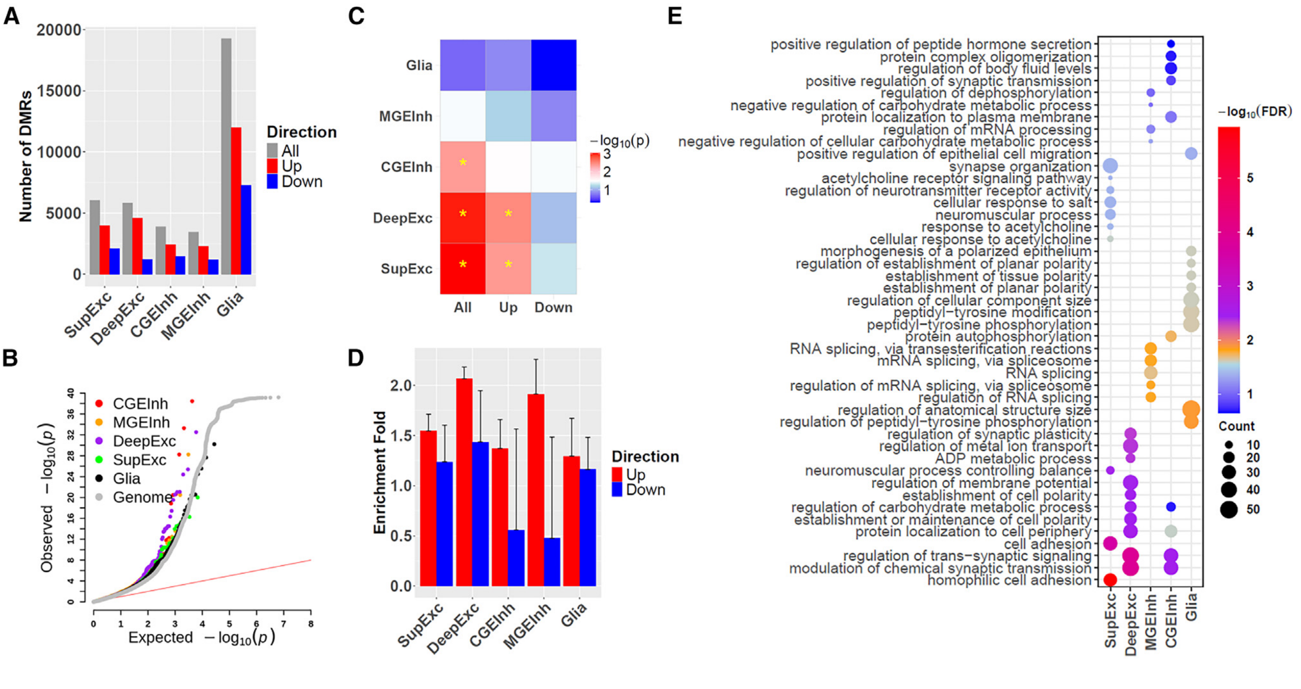

Figure 7. scMeFormer enhances the detection of SCZ-associated DMRs

(A) The number of SCZ-associated DMRs detected in each cell type, stratified by direction of effect.

(B) Quantile-quantile (Q-Q) plot of SCZ GWAS p values for SNPs located within or near DMRs detected in each cell type.

(C) Gene-set-based association with SCZ for genes linked to DMRs in each cell type, where "up" and "down" on the x axis represent genes linked to up-regulated and down-regulated DMRs, respectively, and "all" represents genes linked to DMRs regardless of the direction of the effect.

(D) Enrichment fold of genes linked to DMRs for differentially expressed genes identified in a single-cell RNA-seq study of SCZ. Error bars indicate the standard error of enrichment fold.

(E) Top 10 enriched Gene Ontology terms for genes linked to DMRs through chromatin loops.

See also Figures S11 and S12 and Tables S13–S15.

nonoverlapping 100 kb bins, we first clustered these nuclei into five major cell types: two excitatory neuron subtypes from the superficial (SupExc) and deep cortical (DeepExc) layers, two inhibitory neuron subtypes from the cortical ganglionic eminence (InhCGE) and the medial ganglionic eminence (InhMGE), and a glial cell type. We then clustered the same set of nuclei using imputed DNAm levels of CpGs in nonoverlapping 100 kb bins. Clusters from imputed data closely mirror the clusters obtained using CpHs (Figure S11), indicating that the imputation process preserved data properties related to cell type identity.

Next, we aimed to identify SCZ-associated DMRs within each cell type. Remarkably, while no DMRs were detected before imputation, imputation revealed thousands of DMRs across the five cell types (Figure 7A; Table S13). Notably, the number of up-regulated DMRs (indicating increased DNAm levels in SCZ) was substantially higher than the number of down-regulated DMRs. This observation aligns with the recent single-cell study of the SCZ transcriptome,[25] which found that most dysregulated genes in SCZ were down-regulated, given the negative correlation between DNAm and gene expression. Further, we observed that SNPs within or near DMRs (within 5 kb) showed stronger enrichment for SCZ genome-wide association study (GWAS) signals,[26] particularly in neuronal cell types, compared to SNPs across the genome (Figure 7B). This trend was primarily driven by up-regulated DMRs and less so by down-regulated DMRs (Figure S12), suggesting a pivotal role of increased DNAm in SCZ pathogenesis.

To assess the regulatory impact of these DMRs, we linked them to potential target genes using cell-type-specific chromatin loops identified in a previous study.[27] We then conducted gene set analysis with MAGMA[28] to determine whether these target genes were enriched for SCZ GWAS signals. Our analysis revealed significant enrichment of SCZ GWAS signals among genes linked to DMRs in two excitatory neuron subtypes (SupExc and DeepExc) and one inhibitory neuron subtype (InhCGE) (false discovery rate [FDR] < 0.05) (Figure 7C; Table S14). Stratified analysis by DMR direction indicated that this enrichment was primarily driven by genes linked to up-regulated DMRs, further supporting the role of increased DNAm in SCZ pathogenesis.

We further explored whether genes linked to DMRs were enriched for differentially expressed genes (DEGs) from a recent single-cell RNA sequencing (RNA-seq) study of SCZ,[25] matched by the five broad cell types defined in our study. We found that genes linked to up-regulated DMRs were significantly enriched for down-regulated DEGs in the two excitatory neuron subtypes; however, no such enrichment was observed for other cell types, nor was there enrichment for up-regulated DEGs among genes linked to down-regulated DMRs (Figure 7D; Table S15). Gene Ontology enrichment analysis further revealed that genes linked

to DMRs were enriched for synaptic signaling, particularly in the two excitatory neuron subtypes and one inhibitory neuron subtype (InhCGE) (Figure 7E; Table S16). Analysis by DMR direction suggested that this enrichment was primarily driven by genes linked to up-regulated DMRs (Table S16). Together, these findings from GWASs, DMRs, and DEGs highlight the critical role of excitatory neurons in SCZ, potentially mediated by up-regulated DMRs.

Finally, we compiled all candidate genes linked to up-regulated DMRs in each cell type that also showed evidence of differential expression ($p_{adj} < 0.05$), along with their GWAS signals (MAGMA gene-based p value), generating a list of genes for further investigation (Table S17). This list includes well-established genes implicated in SCZ that showed converging evidence—up-regulated DMRs, down-regulated DEGs, and significant gene-based p values from MAGMA ($<2.5 \times 10^{-6}$), such as *GRIN2A* (glutamate ionotropic receptor NMDA type subunit 2A), *RBFOX1* (RNA binding fox-1 homolog 1), and *NCAM1* (neural cell adhesion molecule 1).

DISCUSSION

Single-cell DNAm profiling technologies provide unprecedented opportunities to explore the epigenetic landscape at single-cell resolution. However, many of these technologies suffer from a high missing rate of CpG sites, limiting their full potential to uncover the epigenetic mechanism underlying various biological processes and diseases. Previous deep learning models have attempted to impute CpG methylation status in single cells.[13–15] However, these models face challenges in scaling to thousands of cells, a scenario that is becoming increasingly popular. In this study, we introduced scMeFormer, a transformer-based deep learning model designed to efficiently impute DNAm states across thousands of cells. Unlike earlier models, scMeFormer scales effectively to large datasets, achieving training times of approximately 72 h per single-nucleus DNAm dataset if trained from scratch using four NVIDIA A100 GPUs. Notably, the architecture of scMeFormer allows it to be initially trained on a single dataset and then fine-tuned for new datasets or even across species. This transfer learning strategy maintains robust performance with minimal loss in predictive accuracy compared to training from scratch while significantly reducing computational time; fine-tuning each dataset took only about 6 h in our study. In contrast, training previous deep learning models (DeepCpG, Transformer CpG, and GraphCpG) in our benchmarking was highly computationally intensive, requiring approximately 72 h to converge, despite being trained on just 118 cells selected from the sn-m3C-seq dataset. We further estimated the time required for each model to be trained for 10 epochs as the number of cells increased. While the training times for DeepCpG, Transformer CpG, and GraphCpG scaled linearly or near exponentially with the number of cells, scMeFormer exhibited a near-constant training time, remaining largely unaffected by cell numbers (Table S18). These findings underscore scMeFormer's superior accuracy and efficiency for large-scale single-cell DNAm analysis.

Remarkably, scMeFormer exhibits the ability to impute DNAm states with high fidelity, even when coverage was reduced to only 10% of CpG sites through downsampling, as evidenced by its successful recovery of cell type clusters and identification of cell-type-specific DMRs. Furthermore, we designed a unique filtering score that enables quality control of the imputed data: more stringent filtering scores yield higher-quality imputed data, though they reduce the number of CpG sites imputed in each cell. As a case study, we applied scMeFormer to a single-nucleus DNAm dataset generated from the prefrontal cortex of patients with SCZ and neurotypical controls. This led to the identification of thousands of SCZ-associated DMRs that would have remained undetectable without imputation. Most of these DMRs were up-regulated in patients and showed enrichment for SCZ GWAS signals and down-regulated DEGs in excitatory neurons, underscoring the critical role of excitatory neurons in SCZ.

While scMeFormer is specifically designed for single-cell DNAm data imputation, the foundational principles and modular architecture of our model have potential applications across other single-cell omics data. scMeFormer comprises two main modules that capture both local patterns and long-range dependencies—features that are common across various omics modalities. For instance, in single-cell Hi-C data, the modules could be reconfigured to learn chromatin contact patterns from DNA sequences and borrow information of neighboring contacts within and across cell types. Similarly, for single-cell ATAC-seq data, the architecture could be tailored to model accessible regions and their potential interactions. The transformer component of scMeFormer is particularly valuable due to its ability to model long-range dependencies, making it well suited for capturing complex interactions such as enhancer-promoter loops. Future work exploring these adaptations could expand the utility of scMeFormer, making it a valuable tool in the broader field of single-cell omics research.

Limitations of the study

We acknowledge four limitations of our current model and potential areas for future work. First, scMeFormer relies on input DNA sequences from the reference genome, which does not precisely align with the DNA sequences of the study samples. The model's performance could be further improved by utilizing DNA sequences and DNAm data from the same individuals. Second, the imputation quality metric relies on the assumption that target and neighboring CpG sites show consistent methylation levels. However, this may not always hold, particularly in regions with heterogeneous methylation patterns. Developing more robust evaluation metrics will be an important direction of future work. Third, while scMeFormer demonstrated strong overall performance, it faced challenges in imputing CpG sites with high variability across cells. Additionally, we observed noticeably stronger performance in lowly methylated regions (e.g., TssA and bivalent regions) compared to highly methylated regions (e.g., Tx and Quies). This discrepancy may indicate potential systemic biases or intrinsic complexities related to transcription-linked methylation biology, highlighting the need for further investigation. Fourth, scMeFormer currently focuses on CpG sites imputation. Expanding its capabilities to CpH sites, known for their crucial roles in neurons, would enhance its utility. Despite these limitations, our study demonstrates the power of

deep learning for single-cell DNAm state imputation, and we anticipate that scMeFormer will serve as a valuable tool for advancing single-cell DNAm studies.

RESOURCE AVAILABILITY

Lead contact

Requests for further information and resources should be directed to and will be fulfilled by the lead contact, Shizhong Han (Shizhong.Han@libd.org).

Materials availability

This study did not generate new unique reagents.

Data and code availability

- This paper analyzes existing, publicly available data. The accession numbers for the datasets are listed in the key resources table.
- Processed single-nucleus DNAm data generated in this study have been deposited into GEO (GEO: GSE285847).
- All original code has been deposited at GitHub (https://github.com/LieberInstitute/scMeformer) and is publicly available. A permanent DOIs from Zenodo (https://doi.org/10.5281/zenodo.14727350) is listed in the key resources table.
- Any additional information required to reanalyze the data reported in this paper is available from the lead contact upon request.

ACKNOWLEDGMENTS

We are grateful for the vision and generosity of the Lieber and Maltz families, who made this work possible, and the families who donated to this research and for computing support from the Joint High Performance Computing Exchange (JHPCE) facility in the Department of Biostatistics at the Johns Hopkins Bloomberg School of Public Health. We would like to thank the two anonymous reviewers for their thoughtful criticisms, comments, and suggestions on early versions of the manuscript. This work is supported by National Institutes of Health grants R01MH121394 (S.H.) and R01MH112751 (S.H.).

AUTHOR CONTRIBUTIONS

Conceptualization, J.Z., D.R.W., and S.H.; methodology, J.Z., C.L., and S.H.; investigation, J.Z., C.L., H.L., M.G.H., R.E.S., and S.H.; visualization, J.Z., M.G.H., and S.H.; funding acquisition, S.H.; project administration, S.H.; supervision, S.H.; writing – original draft, J.Z. and S.H.; writing – review & editing, J.Z., C.L., H.L., M.G.H., R.E.S., J.E.K., T.M.H., J.R.E., D.R.W., and S.H.

DECLARATION OF INTERESTS

D.R.W. serves on the scientific advisory boards of Sage Therapeutics and Pasithea Therapeutics. J.R.E. serves on the scientific advisory board of Zymo Research.

STAR★METHODS

Detailed methods are provided in the online version of this paper and include the following:

- KEY RESOURCES TABLE
- EXPERIMENTAL MODEL AND SUBJECT DETAILS
- METHOD DETAILS
    - Single-nucleus DNAm datasets used for model training
    - scMeformer architecture
    - DNA module
    - CpG module
    - Fully connected network
    - Model training
    - Comparison with alternative models
- QUANTIFICATION AND STATISTICAL ANALYSIS
    - Cell type-specific DMRs
    - Stratified LD score regression
    - Single-nucleus DNAm data from schizophrenia cases and controls
    - Assignment of DMRs to target genes
    - Gene-set analysis of SCZ GWAS signals
    - Enrichment analysis for SCZ-associated differentially expressed genes

SUPPLEMENTAL INFORMATION

Supplemental information can be found online at https://doi.org/10.1016/j.xgen.2025.100774.

Received: July 12, 2024
Revised: November 13, 2024
Accepted: January 24, 2025
Published: February 21, 2025

REFERENCES

1. Greenberg, M.V.C., and Bourc'his, D. (2019). The diverse roles of DNA methylation in mammalian development and disease. Nat. Rev. Mol. Cell Biol. *20*, 590–607. https://doi.org/10.1038/s41580-019-0159-6.
2. Ahn, J., Heo, S., Lee, J., and Bang, D. (2021). Introduction to Single-Cell DNA Methylation Profiling Methods. Biomolecules *11*, 1013. https://doi.org/10.3390/biom11071013.
3. Luo, C., Hajkova, P., and Ecker, J.R. (2018). Dynamic DNA methylation: In the right place at the right time. Science *361*, 1336–1340. https://doi.org/10.1126/science.aat6806.
4. Luo, C., Keown, C.L., Kurihara, L., Zhou, J., He, Y., Li, J., Castanon, R., Lucero, J., Nery, J.R., Sandoval, J.P., et al. (2017). Single-cell methylomes identify neuronal subtypes and regulatory elements in mammalian cortex. Science *357*, 600–604. https://doi.org/10.1126/science.aan3351.
5. Lee, D.S., Luo, C., Zhou, J., Chandran, S., Rivkin, A., Bartlett, A., Nery, J.R., Fitzpatrick, C., O'Connor, C., Dixon, J.R., and Ecker, J.R. (2019). Simultaneous profiling of 3D genome structure and DNA methylation in single human cells. Nat. Methods *16*, 999–1006. https://doi.org/10.1038/s41592-019-0547-z.
6. Luo, C., Rivkin, A., Zhou, J., Sandoval, J.P., Kurihara, L., Lucero, J., Castanon, R., Nery, J.R., Pinto-Duarte, A., Bui, B., et al. (2018). Robust single-cell DNA methylome profiling with snmC-seq2. Nat. Commun. *9*, 3824. https://doi.org/10.1038/s41467-018-06355-2.
7. Luo, C., Liu, H., Xie, F., Armand, E.J., Siletti, K., Bakken, T.E., Fang, R., Doyle, W.I., Stuart, T., Hodge, R.D., et al. (2022). Single nucleus multi-omics identifies human cortical cell regulatory genome diversity. Cell Genom. *2*, 100107. https://doi.org/10.1016/j.xgen.2022.100107.
8. Kapourani, C.A., and Sanguinetti, G. (2019). Melissa: Bayesian clustering and imputation of single-cell methylomes. Genome Biol. *20*, 61. https://doi.org/10.1186/s13059-019-1665-8.
9. Jiang, L., Wang, C., Tang, J., and Guo, F. (2019). LightCpG: a multi-view CpG sites detection on single-cell whole genome sequence data. BMC Genom. *20*, 306. https://doi.org/10.1186/s12864-019-5654-9.
10. Tang, J., Zou, J., Fan, M., Tian, Q., Zhang, J., and Fan, S. (2021). CaMelia: imputation in single-cell methylomes based on local similarities between cells. Bioinformatics *37*, 1814–1820. https://doi.org/10.1093/bioinformatics/btab029.
11. Danese, A., Richter, M.L., Chaichoompu, K., Fischer, D.S., Theis, F.J., and Colomé-Tatché, M. (2021). EpiScanpy: integrated single-cell epigenomic analysis. Nat. Commun. *12*, 5228. https://doi.org/10.1038/s41467-021-25131-3.
12. Wolf, F.A., Angerer, P., and Theis, F.J. (2018). SCANPY: large-scale single-cell gene expression data analysis. Genome Biol. *19*, 15. https://doi.org/10.1186/s13059-017-1382-0.

13. Angermueller, C., Lee, H.J., Reik, W., and Stegle, O. (2017). DeepCpG: accurate prediction of single-cell DNA methylation states using deep learning. Genome Biol. *18*, 67. https://doi.org/10.1186/s13059-017-1189-z.

14. De Waele, G., Clauwaert, J., Menschaert, G., and Waegeman, W. (2022). CpG Transformer for imputation of single-cell methylomes. Bioinformatics *38*, 597–603. https://doi.org/10.1093/bioinformatics/btab746.

15. Deng, Y., Tang, J., Zhang, J., Zou, J., Zhu, Q., and Fan, S. (2023). GraphCpG: imputation of single-cell methylomes based on locus-aware neighboring subgraphs. Bioinformatics *39*, btad533. https://doi.org/10.1093/bioinformatics/btad533.

16. Argelaguet, R., Clark, S.J., Mohammed, H., Stapel, L.C., Krueger, C., Kapourani, C.A., Imaz-Rosshandler, I., Lohoff, T., Xiang, Y., Hanna, C.W., et al. (2019). Multi-omics profiling of mouse gastrulation at single-cell resolution. Nature *576*, 487–491. https://doi.org/10.1038/s41586-019-1825-8.

17. Stadler, M.B., Murr, R., Burger, L., Ivanek, R., Lienert, F., Schöler, A., van Nimwegen, E., Wirbelauer, C., Oakeley, E.J., Gaidatzis, D., et al. (2011). DNA-binding factors shape the mouse methylome at distal regulatory regions. Nature *480*, 490–495. https://doi.org/10.1038/nature10716.

18. Hon, G.C., Rajagopal, N., Shen, Y., McCleary, D.F., Yue, F., Dang, M.D., and Ren, B. (2013). Epigenetic memory at embryonic enhancers identified in DNA methylation maps from adult mouse tissues. Nat. Genet. *45*, 1198–1206. https://doi.org/10.1038/ng.2746.

19. Mo, A., Mukamel, E.A., Davis, F.P., Luo, C., Henry, G.L., Picard, S., Urich, M.A., Nery, J.R., Sejnowski, T.J., Lister, R., et al. (2015). Epigenomic Signatures of Neuronal Diversity in the Mammalian Brain. Neuron *86*, 1369–1384. https://doi.org/10.1016/j.neuron.2015.05.018.

20. Amemiya, H.M., Kundaje, A., and Boyle, A.P. (2019). The ENCODE Blacklist: Identification of Problematic Regions of the Genome. Sci. Rep. *9*, 9354. https://doi.org/10.1038/s41598-019-45839-z.

21. Nott, A., Holtman, I.R., Coufal, N.G., Schlachetzki, J.C.M., Yu, M., Hu, R., Han, C.Z., Pena, M., Xiao, J., Wu, Y., et al. (2019). Brain cell type-specific enhancer-promoter interactome maps and disease-risk association. Science *366*, 1134–1139. https://doi.org/10.1126/science.aay0793.

22. O'Leary, L.A., and Mechawar, N. (2021). Implication of cerebral astrocytes in major depression: A review of fine neuroanatomical evidence in humans. Glia *69*, 2077–2099. https://doi.org/10.1002/glia.23994.

23. Zhou, B., Zhu, Z., Ransom, B.R., and Tong, X. (2021). Oligodendrocyte lineage cells and depression. Mol. Psychiatr. *26*, 103–117. https://doi.org/10.1038/s41380-020-00930-0.

24. Wang, H., He, Y., Sun, Z., Ren, S., Liu, M., Wang, G., and Yang, J. (2022). Microglia in depression: an overview of microglia in the pathogenesis and treatment of depression. J. Neuroinflammation *19*, 132. https://doi.org/10.1186/s12974-022-02492-0.

25. Ruzicka, W.B., Mohammadi, S., Fullard, J.F., Davila-Velderrain, J., Subburaju, S., Tso, D.R., Hourihan, M., Jiang, S., Lee, H.C., Bendl, J., et al. (2024). Single-cell multi-cohort dissection of the schizophrenia transcriptome. Science *384*, eadg5136. https://doi.org/10.1126/science.adg5136.

26. Trubetskoy, V., Pardiñas, A.F., Qi, T., Panagiotaropoulou, G., Awasthi, S., Bigdeli, T.B., Bryois, J., Chen, C.Y., Dennison, C.A., Hall, L.S., et al. (2022). Mapping genomic loci implicates genes and synaptic biology in schizophrenia. Nature *604*, 502–508. https://doi.org/10.1038/s41586-022-04434-5.

27. Yu, M., Abnousi, A., Zhang, Y., Li, G., Lee, L., Chen, Z., Fang, R., Lagler, T.M., Yang, Y., Wen, J., et al. (2021). SnapHiC: a computational pipeline to identify chromatin loops from single-cell Hi-C data. Nat. Methods *18*, 1056–1059. https://doi.org/10.1038/s41592-021-01231-2.

28. de Leeuw, C.A., Mooij, J.M., Heskes, T., and Posthuma, D. (2015). MAGMA: generalized gene-set analysis of GWAS data. PLoS Comput. Biol. *11*, e1004219. https://doi.org/10.1371/journal.pcbi.1004219.

29. Schultz, M.D., He, Y., Whitaker, J.W., Hariharan, M., Mukamel, E.A., Leung, D., Rajagopal, N., Nery, J.R., Urich, M.A., Chen, H., et al. (2015). Human body epigenome maps reveal noncanonical DNA methylation variation. Nature *523*, 212–216. https://doi.org/10.1038/nature14465.

30. Finucane, H.K., Bulik-Sullivan, B., Gusev, A., Trynka, G., Reshef, Y., Loh, P.R., Anttila, V., Xu, H., Zang, C., Farh, K., et al. (2015). Partitioning heritability by functional annotation using genome-wide association summary statistics. Nat. Genet. *47*, 1228–1235. https://doi.org/10.1038/ng.3404.

31. Hie, B.L., Kim, S., Rando, T.A., Bryson, B., and Berger, B. (2024). Scanorama: integrating large and diverse single-cell transcriptomic datasets. Nat. Protoc. *19*, 2283–2297. https://doi.org/10.1038/s41596-024-00991-3.

32. Yu, G., Wang, L.G., Han, Y., and He, Q.Y. (2012). clusterProfiler: an R package for comparing biological themes among gene clusters. OMICS *16*, 284–287. https://doi.org/10.1089/omi.2011.0118.

33. Jaffe, A.E., Gao, Y., Deep-Soboslay, A., Tao, R., Hyde, T.M., Weinberger, D.R., and Kleinman, J.E. (2016). Mapping DNA methylation across development, genotype and schizophrenia in the human frontal cortex. Nat. Neurosci. *19*, 40–47. https://doi.org/10.1038/nn.4181.

34. Zhou, J., Chen, Q., Braun, P.R., Perzel Mandell, K.A., Jaffe, A.E., Tan, H.Y., Hyde, T.M., Kleinman, J.E., Potash, J.B., Shinozaki, G., et al. (2022). Deep learning predicts DNA methylation regulatory variants in the human brain and elucidates the genetics of psychiatric disorders. Proc. Natl. Acad. Sci. USA *119*, e2206069119. https://doi.org/10.1073/pnas.2206069119.

STAR★METHODS

KEY RESOURCES TABLE

REAGENT or RESOURCE	SOURCE	IDENTIFIER
Biological samples		
Postmortem prefrontal cortex samples	LIBD brain repository	N/A
Deposited data		
Processed single-nucleus DNAm data	This study	GEO: GSE285847
Human single-nucleus DNAm data (snmC-seq)	Luo et al.[4]	GEO: GSE97179
Human single-nucleus DNAm data (snmC-seq2)	Luo et al.[6]	GEO: GSE112471
Human single-nucleus DNAm data (sn-m3C-seq)	Lee et al.[5]	GEO: GSE130711
Human single-nucleus DNAm data (snmCAT-seq)	Luo et al.[7]	GEO: GSE140493
Mouse single-nucleus DNAm data (scNMT-seq)	Argelaguet et al.[16]	GEO: GSE121708
schizophrenia GWAS summary statistics	Trubetskoy et al.[26]	https://pgc.unc.edu/for-researchers/download-results/
schizophrenia cell type-specific DEGs	Ruzicka et al.[25]	Supplementary tables: https://www.science.org/doi/10.1126/science.adg5136
Software and algorithms		
scMeFormer	This study	GitHub: https://github.com/LieberInstitute/scMeformer Zenodo https://doi.org/10.5281/zenodo.14727350
GraphCpG	Deng et al.[15]	https://github.com/yuzhong-deng/graphcpg
CpG Transformer	De Waele et al.[14]	https://github.com/gdewael/cpg-transformer
DeepCpG	Angermueller et al.[13]	https://github.com/cangermueller/deepcpg
CaMelia	Tang et al.[10]	https://github.com/JxTang-bioinformatics/CaMelia
methylpy	Schultz et al.[29]	https://github.com/yupenghe/methylpy
S-LDSC	Finucane et al.[30]	https://github.com/bulik/ldsc
YAP	Luo et al.[7]	https://cemba-data.readthedocs.io/en/latest/
scanorama	Hie et al.[31]	https://github.com/brianhie/scanorama
MAGMA	de Leeuw et al.[28]	https://cncr.nl/research/magma/
clusterProfiler	Yu et al.[32]	https://github.com/YuLab-SMU/clusterProfiler

EXPERIMENTAL MODEL AND SUBJECT DETAILS

We generated single-nucleus methylomes from the prefrontal cortex of four SCZ cases and four neurotypical controls using the snmCAT-seq technique, but without capturing chromatin accessibility information. The brain tissues were from the brain repository at the Lieber Institute for Brain Development. Details on tissue acquisition, processing, curation, and dissection, were described in prior reports.[33] All eight brain samples were male Caucasian individuals with a mean age of 42 years old in both cases and controls.

METHOD DETAILS

Single-nucleus DNAm datasets used for model training

Datasets include four single-nucleus DNAm datasets generated by four different technologies we developed: snmC-seq,[4] snmC-seq2,[6] sn-m3C-seq,[5] and snmCAT-seq,[7] all applied to human postmortem brain tissues. To show generalizability of our model, we also included a single-nucleus dataset from mouse embryos generated by scNMT-seq.[16] Detailed information regarding each technology and the bioinformatics procedures for data processing have been described in original studies. Briefly, snmC-seq was a multiplexed single-nucleus DNAm profiling technique we initially developed, which was used to analyze the methylomes of 2,784 neurons isolated from the human frontal cortex. snmC-seq2 was the improved version of snmC-seq with increased read mapping and enhanced throughput, and was used to profile the methylomes of 3,072 nuclei obtained from postmortem prefrontal cortex. sn-m3C-seq was a single-cell multi-omics technique that jointly profiles chromatin conformation and DNAm from the same cells and was applied to profile 4,237 nuclei from human BA10 cortical tissue. snmCAT-seq was developed to jointly profile methylome, chromatin accessibility, and transcriptome from the same cells, and was applied to profile 4,357 nuclei isolated from postmortem human BA10 cortical tissue. scNMT-seq was a single-cell multi-omics technique designed to profile nucleosome positioning, DNAm, and transcriptomes simultaneously. This technique was applied to profile 1,105 single cells isolated from mouse embryos across four developmental stages, capturing the transition from pluripotency to primary germ-layer specification.

scMeformer architecture

scMeformer contains three main modules: a DNA module, a CpG module, and a fully connected network. The input to the model includes two modalities: a one-hot encoded DNA sequence of 2 kb and the DNAm levels of 100 neighboring CpGs around the target CpG in each cell cluster. The output of the model is the predicted methylation states of the target CpG in each cell across all cells. Table S19 provides details for key hyperparameters used in the model. Below are descriptions of each module.

DNA module

The DNA module employs the INTERACT architecture developed in our previous study,[34] which consists of two sub-modules: a convolutional neural network (CNN) and the encoder of transformer. The CNN comprises three convolutional layers, a batch normalization layer, a max-pooling layer, and a dropout layer. Each convolutional layer uses 256 kernels of size 10 to learn motifs from DNA sequences, and each kernel is activated by a rectified linear unit (ReLU) function. The max-pooling layer (step size = 20bp) is used after the three convolutional layers to capture motifs learned by the convolutional layers. The batch normalization layer is used after the max-pooling layer to improve training speed and stability. The dropout layer is employed to prevent overfitting. The dropout rate in this layer is set to 0.5.

The encoder of transformer consists of a stack of eight identical layers, which takes the CNN output as input to learn distant features that may act jointly. Each layer in the transformer encoder employs two sub-layers. The first sub-layer is a multi-head self-attention layer that learns the attention between any two features at different positions. The second sub-layer is a simple, position-wise fully connected feedforward network. After both sub-layers, a normalization layer is employed to speed up training and improve training stability. Additionally, a dropout layer with a rate of 0.1 is employed to prevent overfitting. Each sub-layer in the encoder has a residual connection to help mitigate the vanishing gradient problem. Residual connections are often used in deep neural networks to prevent the network from forgetting important features of the input during training.

CpG module

The CpG module employs a similar architecture as the DNA module. Given a target CpG site the model aims to predict, the CpG module takes as input the DNAm levels of 100 CpG sites around the target CpG site (excluding the target CpG site itself) across pre-defined cell clusters. To create this input, all single cells are initially clustered into clusters based on the DNAm levels of CpG sites within non-overlapping 100kb bins. For a given CpG site, its DNAm level in each cluster is determined by dividing the number of methylated reads by the total number of reads in that cluster. If the cells are clustered into n clusters, the input to the CpG module is a $100 \times n$ matrix. The CNN identifies local patterns of CpG methylation within each cluster through a 1-dimensional convolutional kernel (256 kernels), generating a feature matrix of dimensions $256 \times K$ for each cluster. The mean feature matrix across clusters is then fed into a position-wise transformer to capture relationships among positions. The feature matrix at the position of target CpG is passed to the cluster-wise transformer to capture relationships among cell clusters.

Fully connected network

The fully connected network comprises a hidden layer with 768 units, a dropout layer, and an output layer. The dropout layer is designed to prevent overfitting and uses a drop rate of 0.1. The output layer applies the sigmoid function to scale the predicted values between 0 and 1. The number of units in the output layer equals the number of cells. The input to the fully connected network is the concatenation of the output from the DNA module and the CpG module. Each unit in the output layer represents the DNAm state of the target CpG site in the corresponding cell and is indicated by a value of either 0 or 1.

Model training

We divided CpG sites into three subsets by chromosomes for model training, validation, and evaluation. The training set consisted of CpG sites on chromosomes 1 to 20, while CpG sites on chromosome 21 were used as the validation set for model tuning, and CpG sites on chromosome 22 were used as the independent testing set to evaluate the model prediction performance. Approximately 5% of CpG sites were covered by at least one read in a single cell across the five datasets and were employed for model training, while the remaining CpG sites were not covered by any reads and were not used in model training. We defined DNAm state as 1 for a CpG site if all mapped reads support methylation, and 0 if all mapped reads support unmethylation. We did not include CpG sites for model training if their mapped reads support both methylation and unmethylation. We employed two training strategies for scMeFormer. The first strategy involved training a separate model from scratch for each dataset (scMeFormer(scratch)). The second strategy involved pre-training a model on one dataset, followed by fine-tuning it on a different dataset (scMeFormer(finetune)). Specifically, for scMeFormer(finetune), we pre-trained the model on the snmCAT-seq dataset and fine-tuned it on the snmC-seq and sn-m3C-seq datasets; we also pre-trained the model on the sn-m3C-seq dataset and fine-tuned it on snmC-seq2, snmCAT-seq, and mouse embryo datasets.

Comparison with alternative models

We compared scMeformer to four alternative models: the CNN model, the cluster model, the scMeformer model but with only the DNA module, and the scMeformer model but with only the CpG module. The CNN model also consists of three modules: a DNA module, a CpG module, and a fully connected network. However, in the CNN model, both the DNA and CpG modules employ a convolutional neural network (the same as described in the DNA module of scMeformer) rather than the transformer encoders. The cluster model first clusters cells into clusters based on DNAm levels of CpGs within non-overlapping 100kb bins. For each cell in a cluster, the methylation state for a CpG site not covered by any reads is imputed by known methylation states of this CpG site in cells of the same cluster.

We further compared scMeFormer with three prior deep learning models GraphCpG,[15] CpG Transformer[14], DeepCpG,[13] and CaMelia,[10] a gradient boosting-based model. Due to the scalability limitation of these models, we trained them on a randomly selected subset of 118 cells from the sn-m3C-seq dataset. All models were trained using the same setup for the training, validation, and test sets of CpG sites as used for scMeFormer. To estimate the training time required for larger datasets, we ran each model for a limited number of batches at varying number of cells from the sn-m3C-seq dataset, recorded the time, and extrapolated it to estimate the time needed for 10 epochs.

QUANTIFICATION AND STATISTICAL ANALYSIS

Cell type-specific DMRs

We leveraged pre-assigned cell type labels for each nucleus in each dataset from the original studies. We then employed the DMRfind function from methylpy (v1.4.2)[29] to identify cell type-specific DMRs across all cell type pairs. Briefly, DMRfind utilizes a permutation-based root-mean-square test of goodness-of-fit to identify differentially methylated sites (DMS) across samples. Consecutive DMSs within 250 bp are then merged into DMRs. In our imputed dataset, we considered a CpG site with a predicted methylation status as covered by one read supporting methylation, and a predicted unmethylated CpG site as covered by one read supporting unmethylation.

Stratified LD score regression

We performed stratified LD score regression (S-LDSC)[30] to evaluate the enrichment of heritability of 18 brain-related traits and disorders. We also included two non-brain traits, human height and type 2 diabetes, as a negative control to examine whether our findings are specific to brain disorders. We downloaded GWAS summary statistics of each trait from the sources listed in Table S20. Following recommendations from the LDSC resource website (https://alkesgroup.broadinstitute.org/LDSCORE), S-LDSC was run for each list of variants with the baseline LD model v2.2 that included 97 annotations to control for the LD between variants with other functional annotations in the genome. We used HapMap Project Phase 3 SNPs as regression SNPs, and 1000 Genomes SNPs of European ancestry samples as reference SNPs, downloaded from the LDSC resource website. To evaluate the unique contribution of annotations to trait heritability, we utilized a metric from S-LDSC: the Z score of per-SNP heritability. This metric allows us to discern the unique contributions of candidate annotations while accounting for contributions from other functional annotations in the baseline model. The p-values were derived from the Z score under the assumption of a normal distribution, using a two-tailed test. FDR was computed from the p-values using the Benjamini & Hochberg procedure.

Single-nucleus DNAm data from schizophrenia cases and controls

We processed sequencing reads by implementing a versatile mapping pipeline (http://cemba-data.readthedocs.io/) for all the methylome-based technologies developed by our group, as detailed in our previous study.[7] After allc files were generated, the methylcytosine (*mc*) and total cytosine basecalls (*cov*) were summed up for each 100kb bin across the hg19 genome for each sequence context (CG, CH). After filtering cells by various mapping metrics, 2,534 nuclei were retained for further analysis. Using DNAm levels of CpHs in nonoverlapping 100kb bins, we clustered these nuclei into five major cell types: two excitatory neuron subtypes from the

superficial and deep cortical layers (SupExc and DeepExc), two inhibitory neuron subtypes from the cortical ganglionic eminence (InhCGE) and the medial ganglionic eminence (InhMGE), and a glial cell type. We also clustered these nuclei using DNAm levels of CpGs in nonoverlapping 100kb bins after imputation employing functions from the scanorama package.[31] We imputed CpGs DNAm states in each cell through fine-tuning the model trained on sn-m3C-seq dataset for each sample we collected for DNAm. We applied a filtering score of 0.3 to the imputed data to enhance its quality, balancing precision and recall based on our evaluation for precision using two broad cell types. We employed the DMRfind function from methylpy to identify SCZ-associated DMRs for each cell type. DMRs were called for regions with at least two DMSs (FDR <0.01) within 250bp, and each DMS had the same direction of effect in at least two samples from either the case or control group.

Assignment of DMRs to target genes

SCZ-associated DMRs detected in each cell type were assigned to target genes by leveraging reported cell type-specific chromatin loops detected in the prefrontal cortex in a previous study.[27] Given the limited number of loops detected in each individual cell type, we combined loops from cell types belonging to the broad neuronal or glial cell types. We then linked DMRs in neuronal cell types (DeepExc, SupExc, CGEInh, MGEInh) to their potential target genes based on chromatin loops collected for the broad neuronal cell type. Similarly, DMRs in glial cell types were linked to potential target genes using loops collected for the broad glial category. We used clusterProfiler[32] for gene ontology enrichment analysis for genes regulated by DMRs detected in each cell type.

Gene-set analysis of SCZ GWAS signals

We used MAGMA[28] gene-set analysis to test for enrichment of SCZ GWAS signals for genes linked to SCZ-associated DMR detected in our dataset. SNPs were assigned to genes if they were located within gene boundaries, based on NCBI 37.3 gene annotation, or within 20 kb upstream or downstream of the gene, to capture regulatory variants. We first performed gene-based association test using SCZ PGC3 GWAS summary statistics of European ancestry samples,[26] employing the "gene-wise" model of MAGMA. Linkage disequilibrium (LD) reference was based on the genotype data of unrelated EUR samples from the 1000 Genomes Project. Gene-set analysis were performed using MAGMA with the "–set-annot" option.

Enrichment analysis for SCZ-associated differentially expressed genes

To evaluate whether genes linked to SCZ-associated DMRs were enriched for SCZ-associated DEGs, we utilized DEG analysis results from a recent scRNA-Seq study of SCZ.[25] To generate a DEG list for each broad cell type defined in our DNAm dataset, we aggregated DEGs ($p_{adj} < 0.05$) identified in the original cell types from the scRNA-Seq study that corresponded to each broad cell type. Fisher's exact test was used to test the enrichment of DEGs among genes linked to DMRs in each broad cell type compared to the background gene set, which were defined as all loop-connected genes in the broad neuronal or glial cell types.

Cell Genomics, Volume *5*

Supplemental information

Deep learning imputes DNA methylation states in single cells and enhances the detection of epigenetic alterations in schizophrenia

Jiyun Zhou, Chongyuan Luo, Hanqing Liu, Matthew G. Heffel, Richard E. Straub, Joel E. Kleinman, Thomas M. Hyde, Joseph R. Ecker, Daniel R. Weinberger, and Shizhong Han

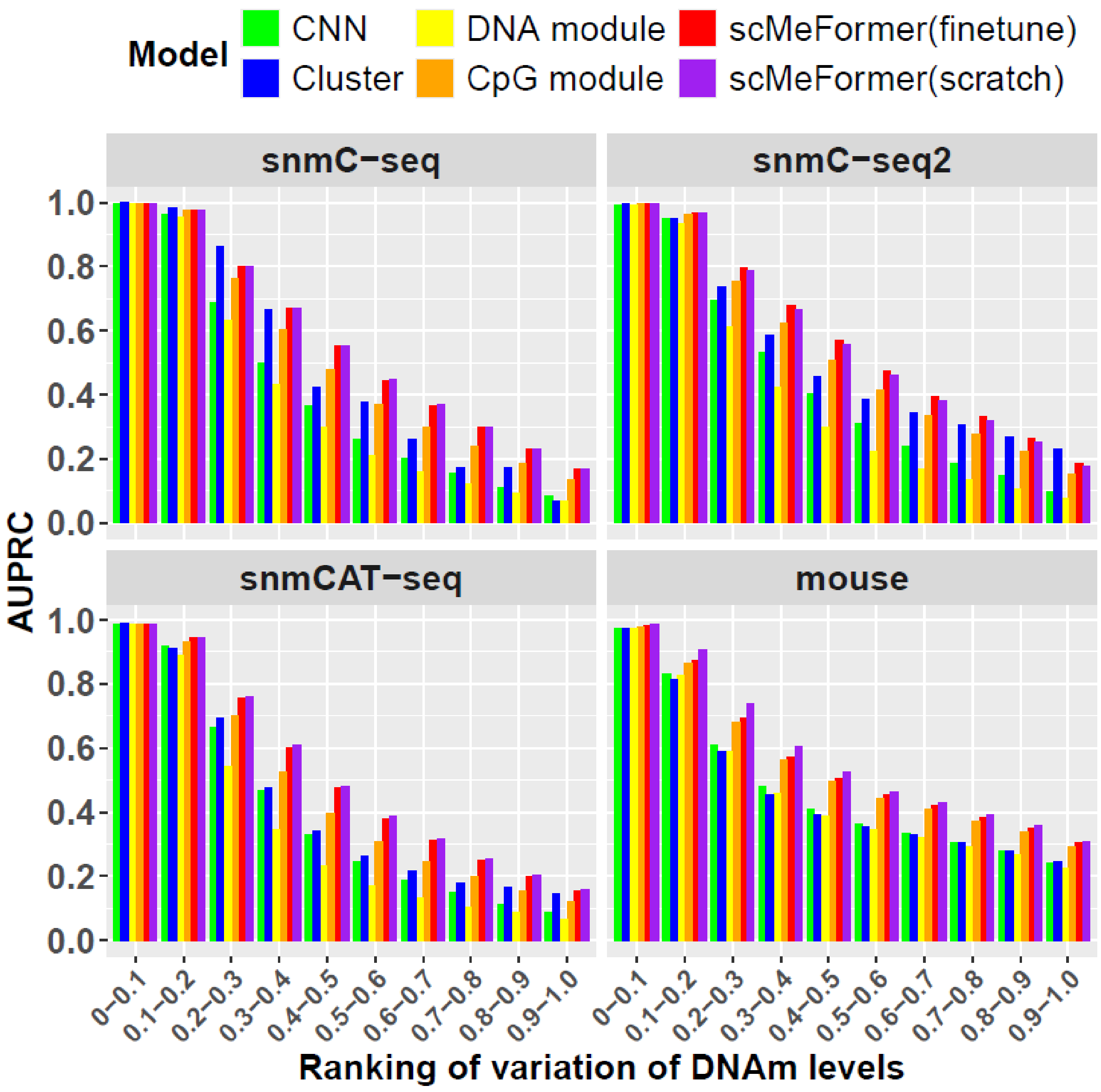

Figure S1. Comparison of model prediction performance between scMeFormer and four alternative models across four single-nucleus DNAm datasets, Related to Figure 2. Comparison was based on subsets of independent testing CpG sites, stratified by their levels of variations across all cells in each dataset. The “0-0.1” group represents the bottom 10% least variable CpG sites. The “0.9-1.0” group represents the top 10% most variable CpG sites.

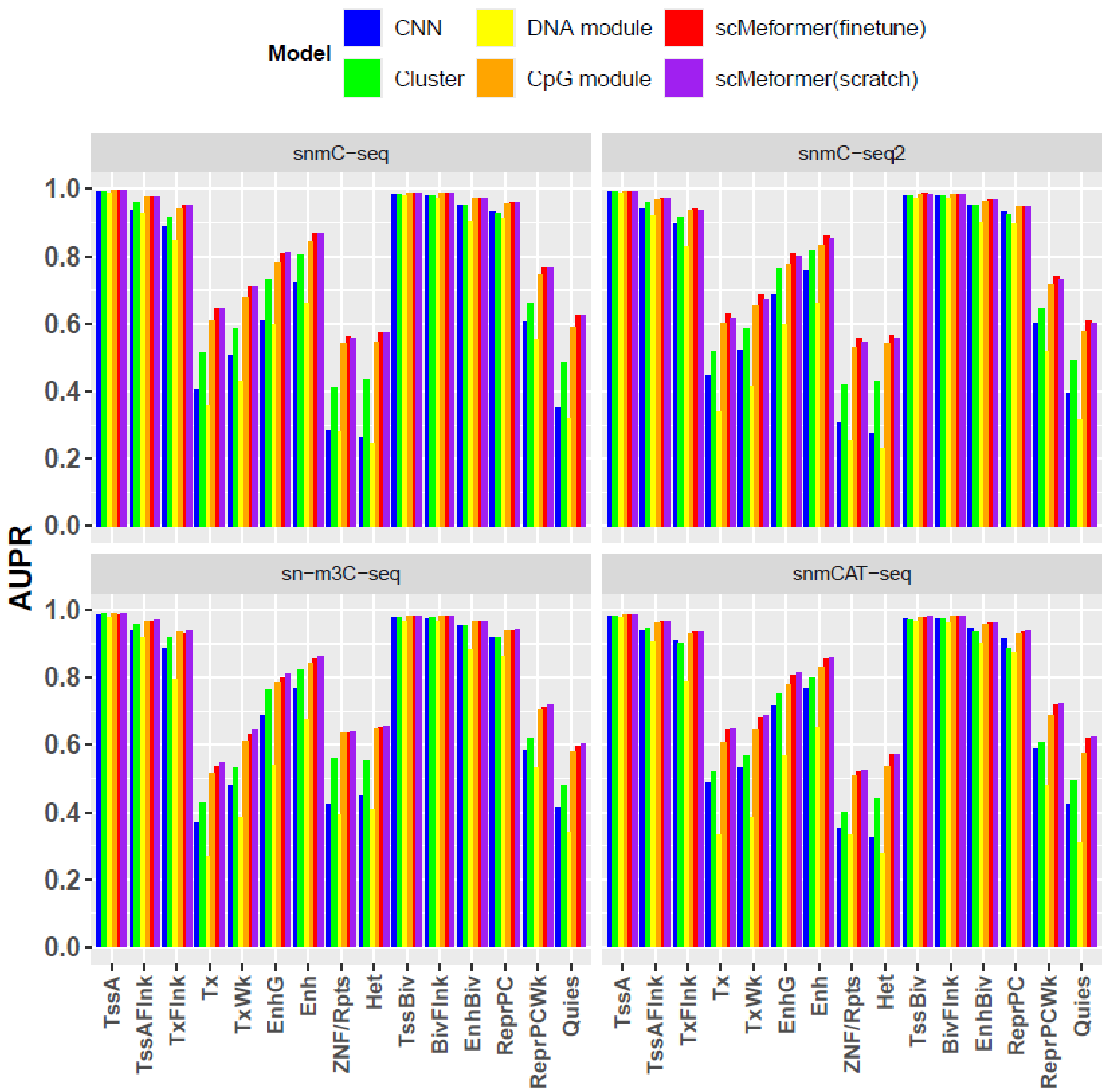

Figure S2. Prediction performance of scMeFormer and alternative models across four single-nucleus DNAm datasets from the human brain, Related to Figure 2 and Table S3. Performance was evaluated on subsets of independent testing CpG sites, stratified by their chromatin states defined in bulk dorsolateral prefrontal cortex derived from the ROADMAP Epigenomics Project.

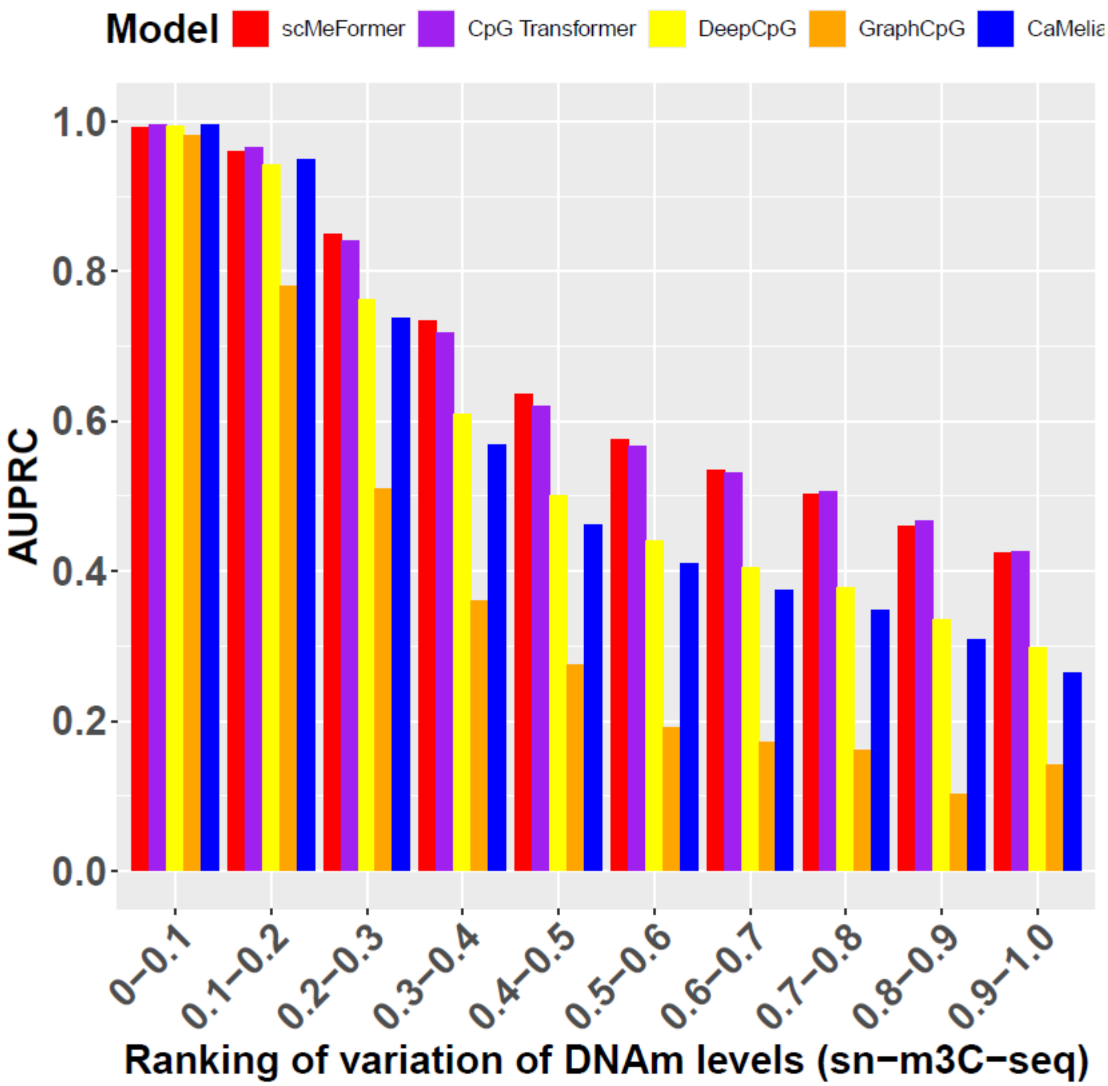

Figure S3. Comparison of model prediction performance between scMeFormer and three previous models (DeepCpG, GraphCpG, and CaMelia), Related to Figure 2. Comparison was based on subsets of independent testing CpG sites, stratified by their levels of variations across all cells in each dataset. The “0-0.1” group represents the bottom 10% least variable CpG sites. The “0.9-1.0” group represents the top 10% most variable CpG sites.

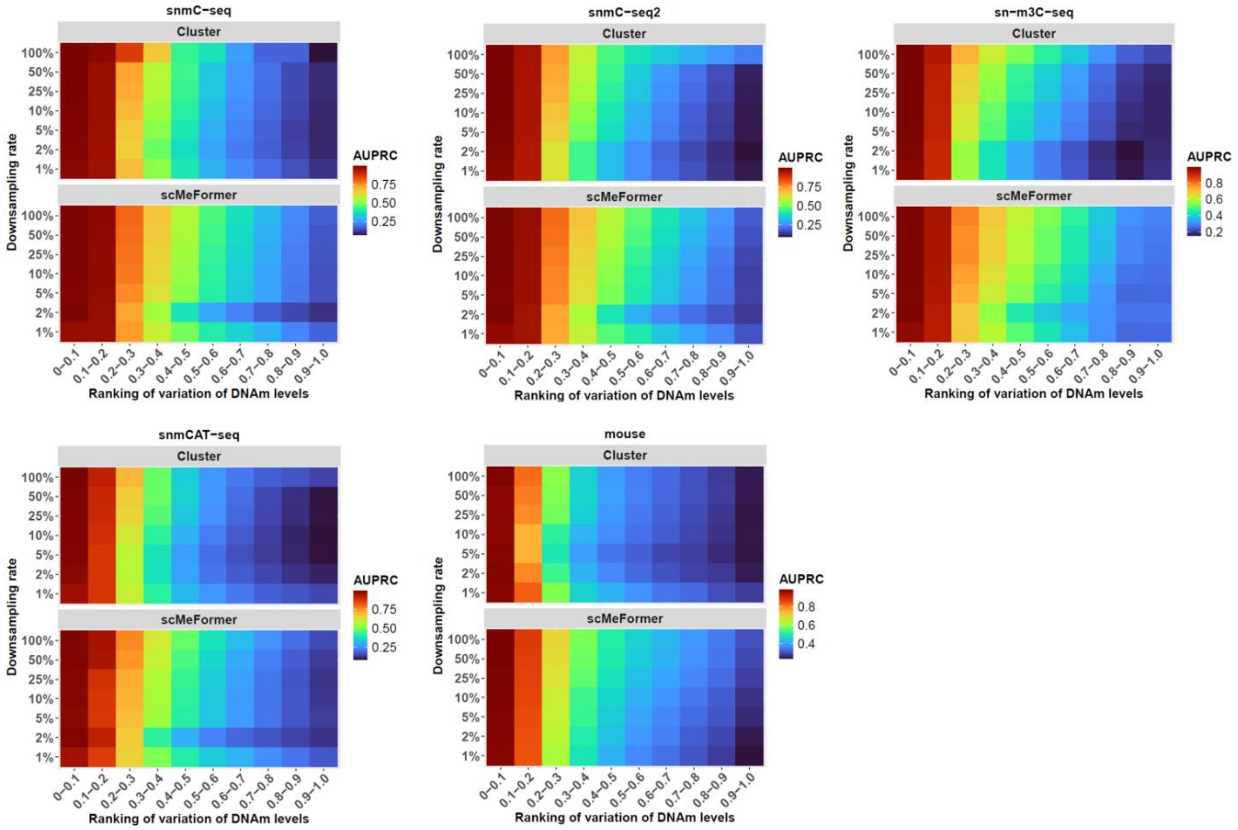

Figure S4. Comparison of prediction performance between scMeformer and the cluster model across five datasets under lower CpG coverage through downsampling, Related to Figure 3 and Table S5. Comparison was based on subsets of independent testing CpG sites, stratified by their levels of variation across cells. The “0-0.1” group represents the bottom 10% least variable CpG sites. The “0.9-1.0” group represents the top 10% most variable CpG sites.

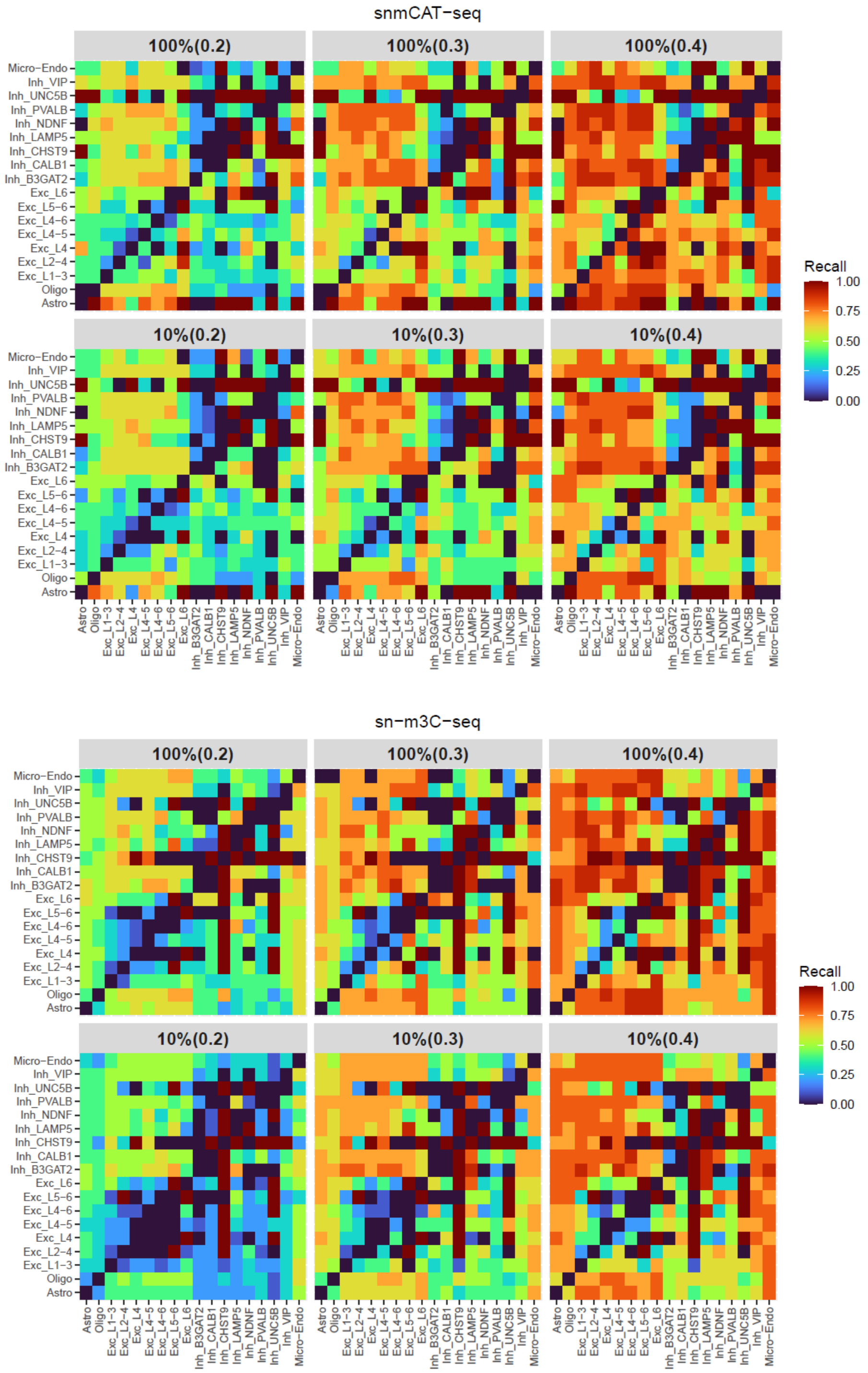

Figure S5. Recall rates for cell type-specific DMRs identified between each pair of cell types in the snmCAT-seq and sn-m3c-seq datasets, Related to Figures 3-4, Table S8. Cell type-specific DMRs were defined by the presence of at least two differentially methylated CpG sites (DMSs). The upper panel in each plot displays recall rates with imputation performed on the original, non-downsampled data, while the lower panel shows recall rates on data downsampled to 10% coverage. Filtering scores applied in each panel are indicated in brackets. The x-axis represents the cell types for which specific DMRs were defined, and the y-axis shows the reference cell types used to identify DMRs for each cell type on the x-axis.

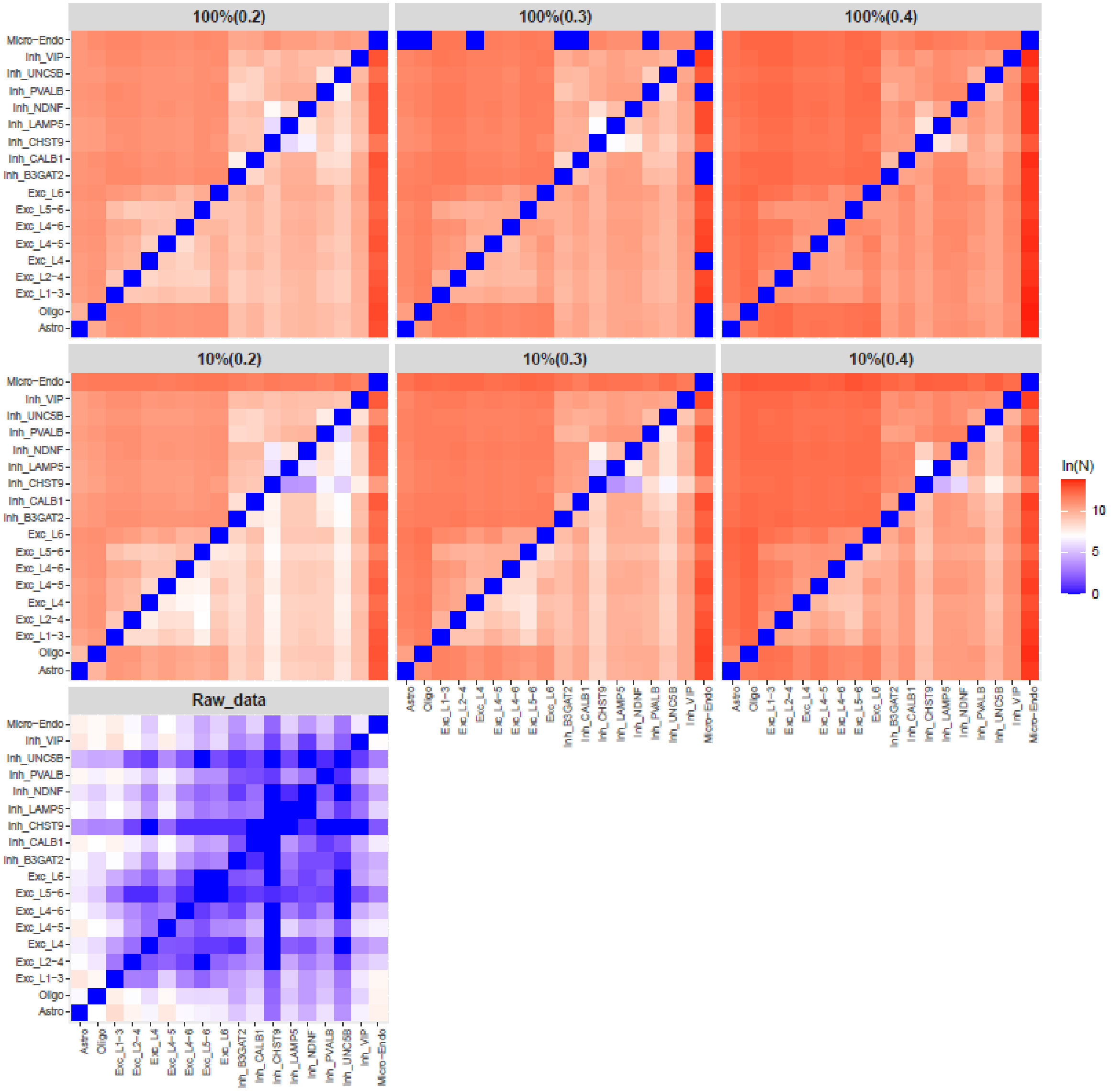

Figure S6. Number of cell type-specific DMRs (natural log scale) detected between each pair of cell types in the sn-m3C-seq dataset, Related to Figures 3-4 and Table S9. The upper panel displays DMR counts identified from imputed data based on the original, non-downsampled dataset; the middle panel shows DMR counts from imputed data based on data downsampled to 10% coverage; and the lower left panel shows DMR counts from raw data without imputation. Filtering scores for imputed data are indicated in brackets. The x-axis represents the cell types for which specific DMRs were defined, and the y-axis shows the reference cell types used to identify DMRs for each cell type on the x-axis.

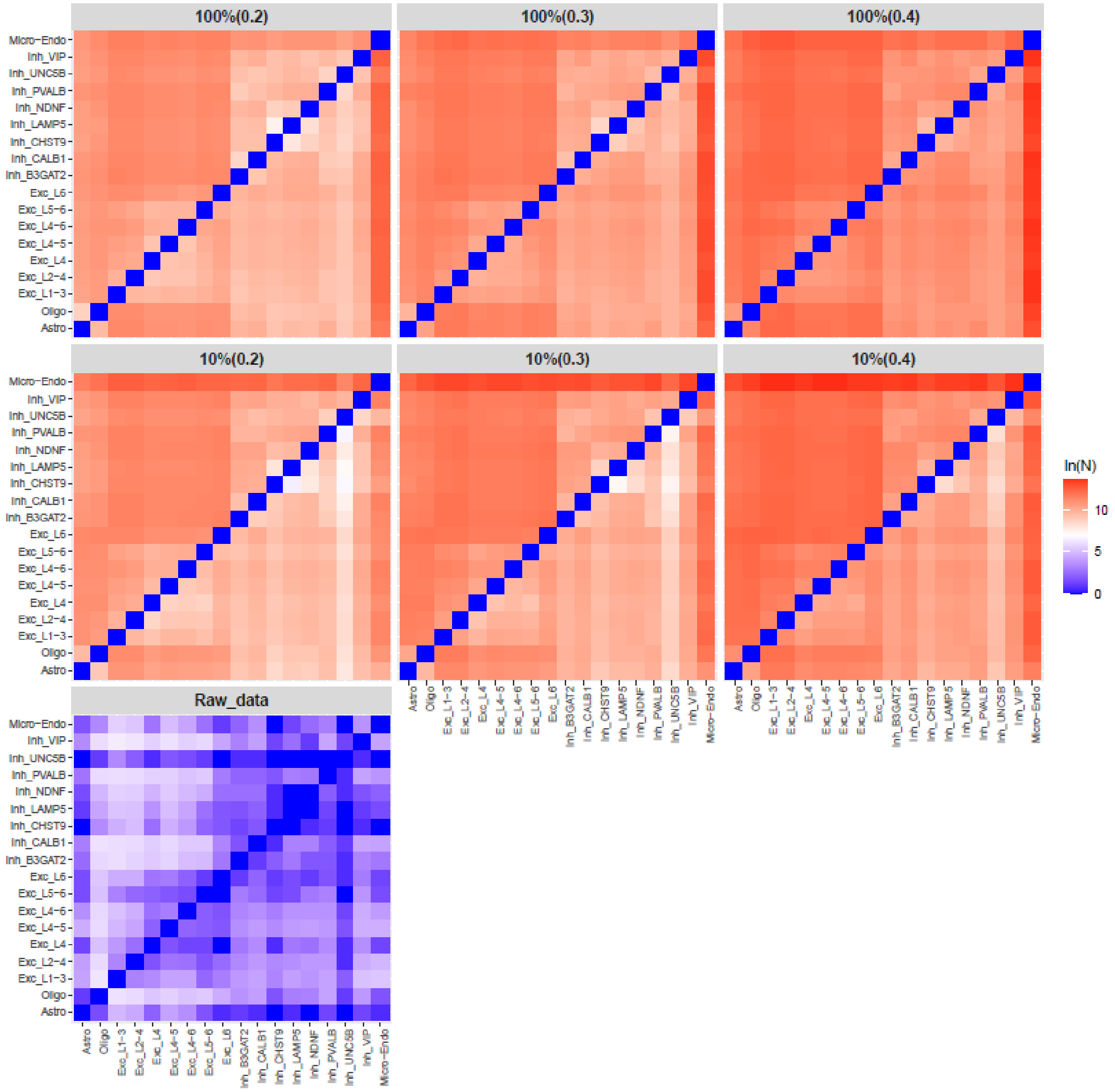

Figure S7. Number of cell type-specific DMRs (natural log scale) detected between each pair of cell types in the snmCAT-seq dataset, Related to Figures 3-4 and Table S9. The upper panel displays DMR counts identified from imputed data based on the original, non-downsampled dataset; the middle panel shows DMR counts from imputed data based on data downsampled to 10% coverage; and the lower left panel shows DMR counts from raw data without imputation. Filtering scores for imputed data are indicated in brackets. The x-axis represents the cell types for which specific DMRs were defined, and the y-axis shows the reference cell types used to identify DMRs for each cell type on the x-axis.

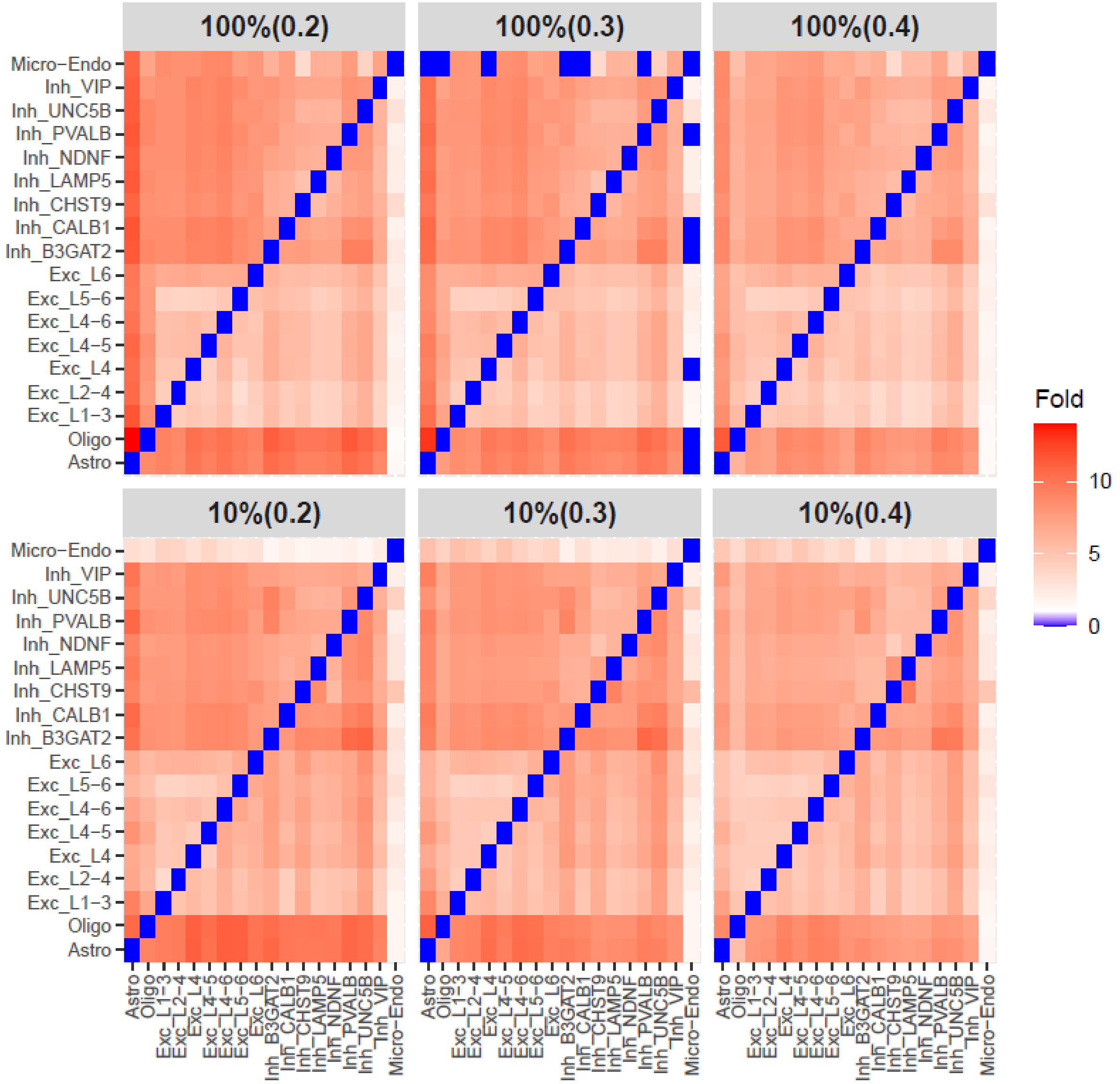

Figure S8. Enrichment fold for H3K27ac marks in the corresponding broad cell type for cell type-specific DMRs detected between each pair of cell types in the sn-m3C-seq dataset, Related to Figures 3-4 and Table S11. The upper panel displays enrichment fold for DMRs identified from imputed data based on the original, non-downsampled dataset, while the lower panel shows enrichment fold for DMRs identified from imputed data based on data downsampled to 10% coverage. Filtering scores for imputed data are indicated in brackets. The x-axis represents cell types for which specific DMRs were defined, and the y-axis shows the reference cell types used to identify DMRs for each cell type on the x-axis.

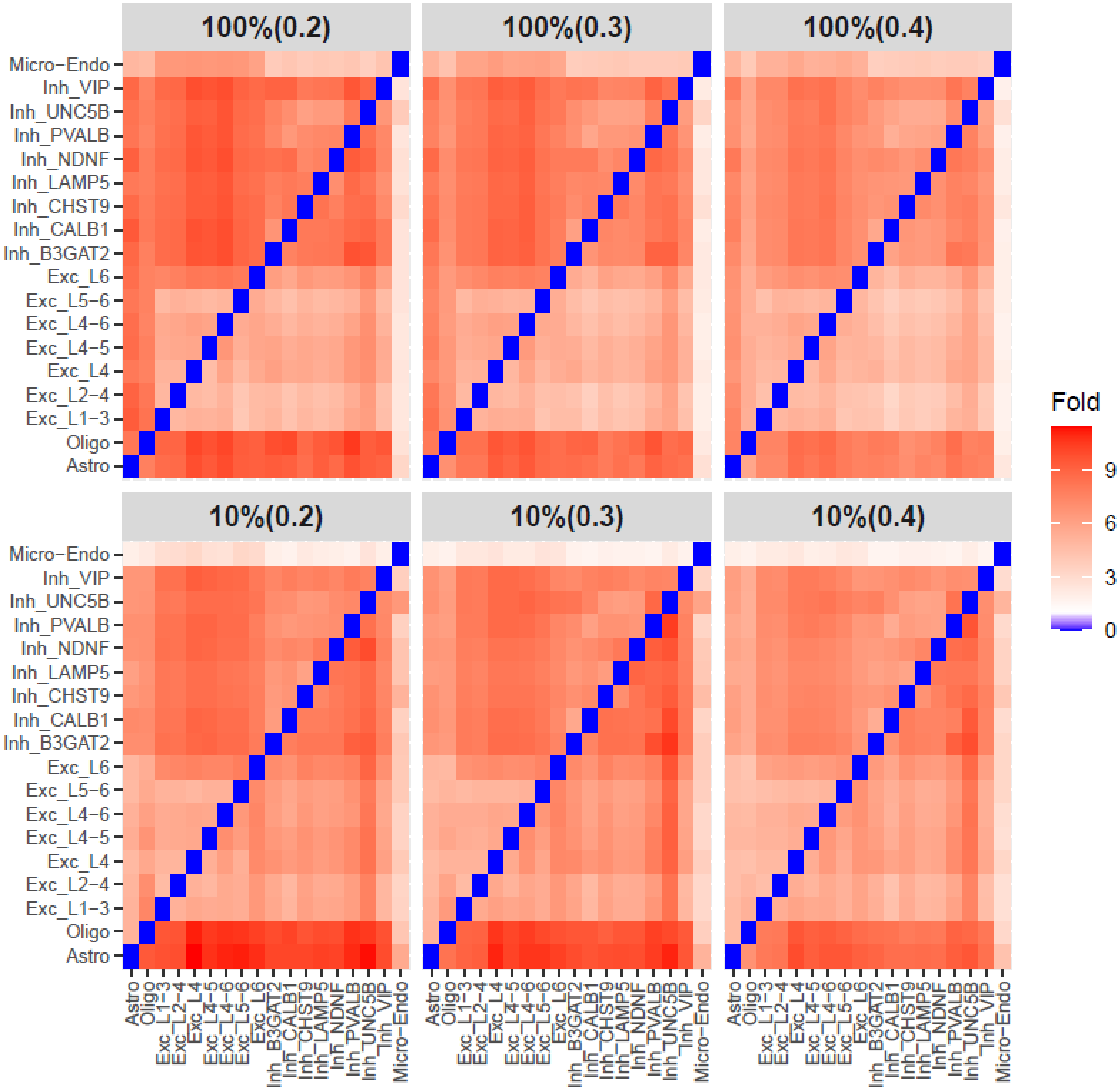

Figure S9. Enrichment fold for H3K27ac marks in the corresponding broad cell type for cell type-specific DMRs detected between each pair of cell types in the snmCAT-seq dataset, Related to Figures 3-4 and Table S11. The upper panel displays enrichment fold for DMRs identified from imputed data based on the original, non-downsampled dataset, while the lower panel shows enrichment fold for DMRs identified from imputed data based on data downsampled to 10% coverage. Filtering scores for imputed data are indicated in brackets. The x-axis represents cell types for which specific DMRs were defined, and the y-axis shows the reference cell types used to identify DMRs for each cell type on the x-axis.

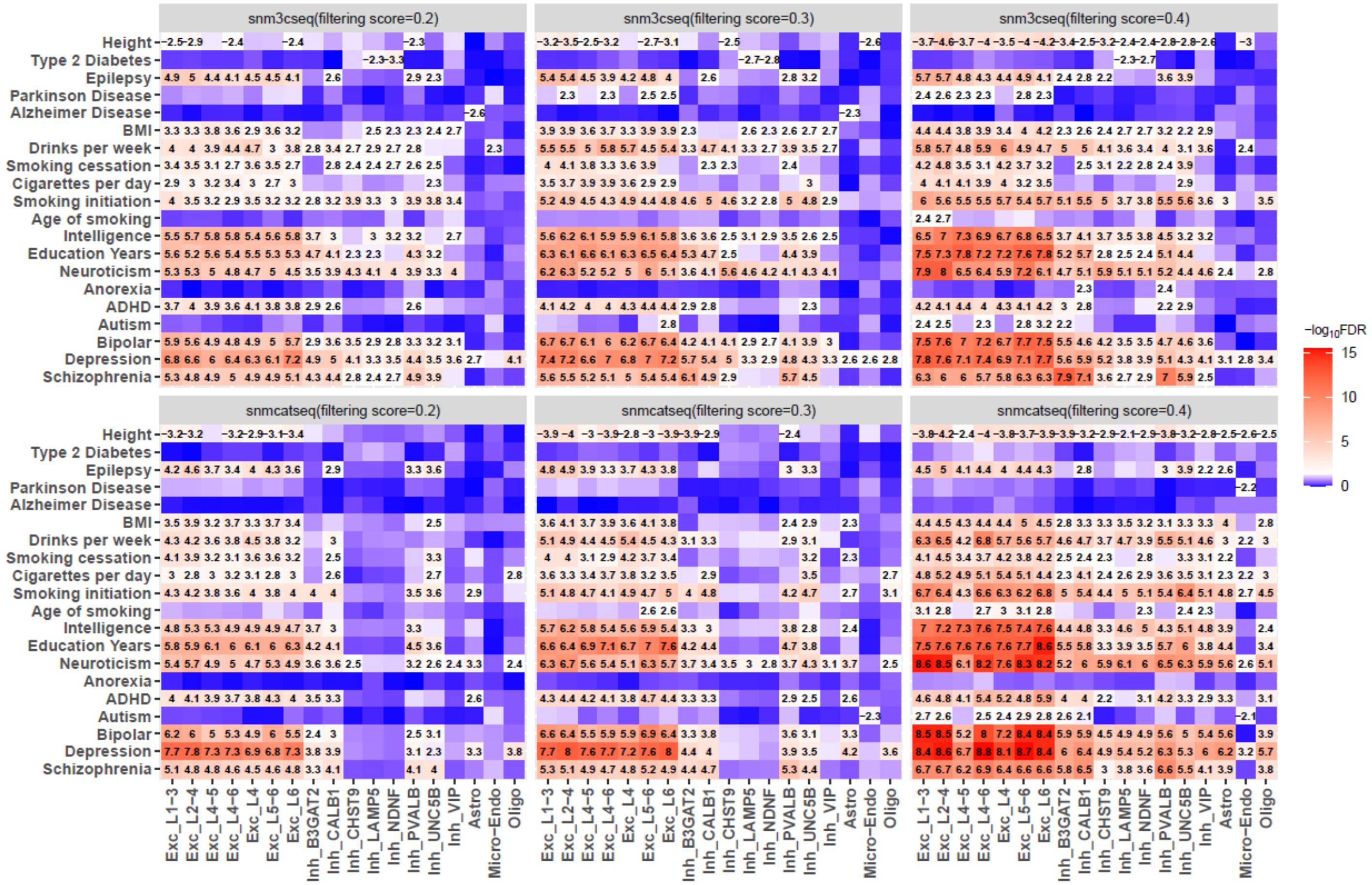

Figure S10. Heritability enrichment analysis for cell type-specific DMRs identified from imputed data of the sn-m3C-seq and snmCAT-seq datasets using three different filtering scores (0.2, 0.3, and 0.4), Related to Figure 6 and Table S12. The x-axis represents cell types for which DMRs were defined relative to all other cell types, while the y-axis lists various GWAS traits. The color scale represents the -$\log_{10}$(FDR) values derived from the z-score of per-SNP heritability calculated by stratified LDSC regression. White color denotes FDR = 0.05. Numbers within the squares show z-scores of per-SNP heritability that are significant after multiple testing correction (FDR < 0.05). Significant negative z-scores indicate a depletion of heritability.

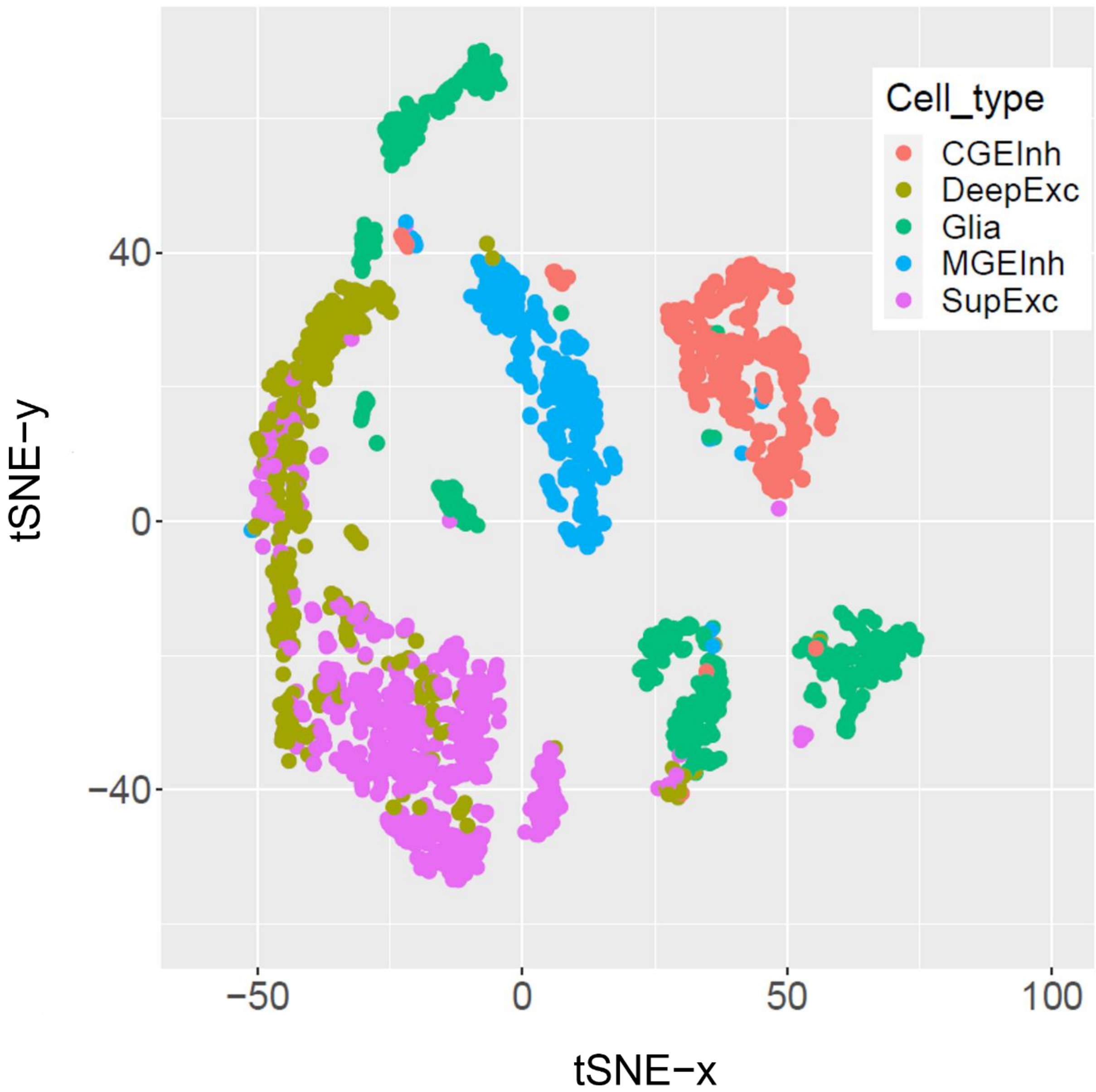

Figure S11. T-SNE plot using imputed CpG sites resemble clusters obtained using CpHs in the raw data, Related to Figure 7 and STAR Methods.

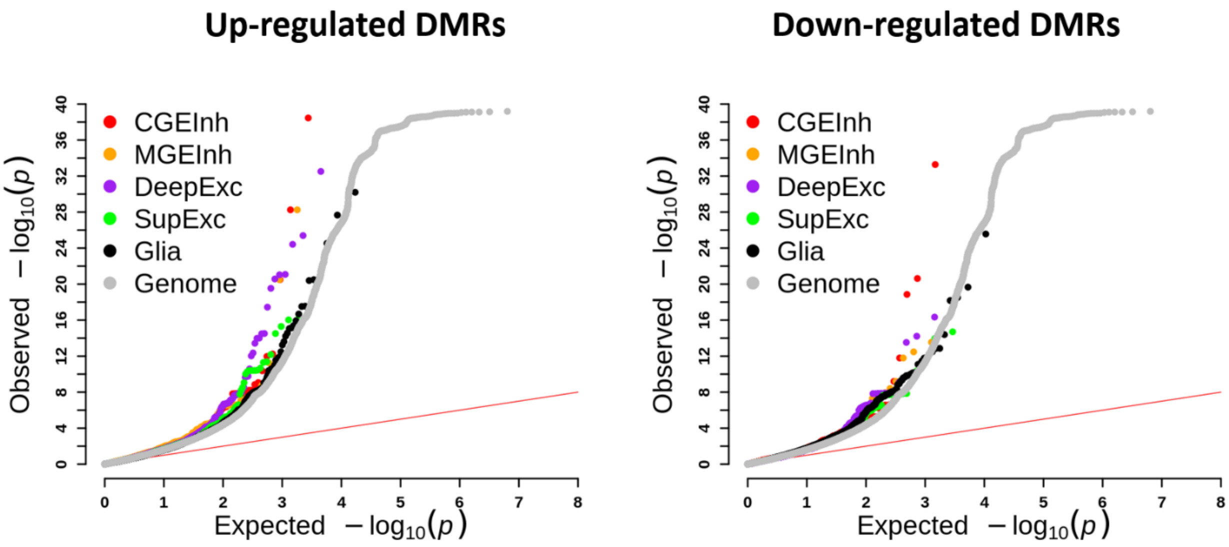

Figure S12. Quantile-Quantile (Q-Q) plot of SCZ GWAS p-values for SNPs located within or near up-regulated DMRs (left panel) and down-regulated DMRs (right panel) identified in each cell type, Related to Figure 7.

Table S1. Prediction performance of scMeFormer and alternative models across five single-nucleus DNAm datasets, Related to Figure 2. Performance was evaluated using all independent testing CpG sites on chromosome 22.

Method	Dataset	AUPRC
scMeFormer(finetune)	snmC-seq	0.906
scMeFormer(scratch)	snmC-seq	0.906
DNA module	snmC-seq	0.803
CpG module	snmC-seq	0.898
Cluster	snmC-seq	0.845
CNN	snmC-seq	0.839
scMeFormer(finetune)	snmC-seq2	0.888
scMeFormer(scratch)	snmC-seq2	0.886
DNA module	snmC-seq2	0.779
CpG module	snmC-seq2	0.878
Cluster	snmC-seq2	0.834
CNN	snmC-seq2	0.827
scMeFormer(finetune)	sn-m3C-seq	0.855
scMeFormer(scratch)	sn-m3C-seq	0.859
DNA module	sn-m3C-seq	0.746
CpG module	sn-m3C-seq	0.848
Cluster	sn-m3C-seq	0.795
CNN	sn-m3C-seq	0.794
scMeFormer(finetune)	snmCAT-seq	0.857
scMeFormer(scratch)	snmCAT-seq	0.858
DNA module	snmCAT-seq	0.719
CpG module	snmCAT-seq	0.839
Cluster	snmCAT-seq	0.773
CNN	snmCAT-seq	0.791
scMeFormer(finetune)	mouse	0.843
scMeFormer(scratch)	mouse	0.844
DNA module	mouse	0.788
CpG module	mouse	0.838
Cluster	mouse	0.731
CNN	mouse	0.792

Table S4. Prediction performance of scMeFormer and the cluster model across five datasets under reduced CpG coverage through downsampling, Related to Figure 3. Performance was assessed using all independent testing CpG sites on chromosome 22.

Proportion	Dataset	Method	AUPRC
100%	snmC-seq	scMeformer	0.90601
100%	snmC-seq	Cluster	0.845974
50%	snmC-seq	scMeformer	0.903674
50%	snmC-seq	Cluster	0.819333
25%	snmC-seq	scMeformer	0.901682
25%	snmC-seq	Cluster	0.825288
10%	snmC-seq	scMeformer	0.897843
10%	snmC-seq	Cluster	0.841087
5%	snmC-seq	scMeformer	0.893423
5%	snmC-seq	Cluster	0.844065
2%	snmC-seq	scMeformer	0.879157
2%	snmC-seq	Cluster	0.829555
1%	snmC-seq	scMeformer	0.867643
1%	snmC-seq	Cluster	0.806888
100%	snmC-seq2	scMeformer	0.888574
100%	snmC-seq2	Cluster	0.834183
50%	snmC-seq2	scMeformer	0.885001
50%	snmC-seq2	Cluster	0.790978
25%	snmC-seq2	scMeformer	0.882625
25%	snmC-seq2	Cluster	0.786746
10%	snmC-seq2	scMeformer	0.876656
10%	snmC-seq2	Cluster	0.799944
5%	snmC-seq2	scMeformer	0.87258
5%	snmC-seq2	Cluster	0.808064
2%	snmC-seq2	scMeformer	0.857521
2%	snmC-seq2	Cluster	0.785802
1%	snmC-seq2	scMeformer	0.847684
1%	snmC-seq2	Cluster	0.759909
100%	sn-m3C-seq	scMeformer	0.854636
100%	sn-m3C-seq	Cluster	0.794826
50%	sn-m3C-seq	scMeformer	0.847252
50%	sn-m3C-seq	Cluster	0.744647
25%	sn-m3C-seq	scMeformer	0.847505
25%	sn-m3C-seq	Cluster	0.740003
10%	sn-m3C-seq	scMeformer	0.839885
10%	sn-m3C-seq	Cluster	0.739125
5%	sn-m3C-seq	scMeformer	0.833364
5%	sn-m3C-seq	Cluster	0.740883

2%	sn-m3C-seq	scMeformer	0.816339
2%	sn-m3C-seq	Cluster	0.738501
1%	sn-m3C-seq	scMeformer	0.800184
1%	sn-m3C-seq	Cluster	0.717626
100%	snmCAT-seq	scMeformer	0.856788
100%	snmCAT-seq	Cluster	0.77363
50%	snmCAT-seq	scMeformer	0.851361
50%	snmCAT-seq	Cluster	0.733988
25%	snmCAT-seq	scMeformer	0.834607
25%	snmCAT-seq	Cluster	0.735762
10%	snmCAT-seq	scMeformer	0.825876
10%	snmCAT-seq	Cluster	0.739009
5%	snmCAT-seq	scMeformer	0.814037
5%	snmCAT-seq	Cluster	0.735743
2%	snmCAT-seq	scMeformer	0.799167
2%	snmCAT-seq	Cluster	0.721702
1%	snmCAT-seq	scMeformer	0.779305
1%	snmCAT-seq	Cluster	0.69699
100%	Mouse_embryo	scMeformer	0.843165
100%	Mouse_embryo	Cluster	0.731858
50%	Mouse_embryo	scMeformer	0.832881
50%	Mouse_embryo	Cluster	0.732428
25%	Mouse_embryo	scMeformer	0.830852
25%	Mouse_embryo	Cluster	0.728033
10%	Mouse_embryo	scMeformer	0.824373
10%	Mouse_embryo	Cluster	0.713479
5%	Mouse_embryo	scMeformer	0.820564
5%	Mouse_embryo	Cluster	0.700707
2%	Mouse_embryo	scMeformer	0.817195
2%	Mouse_embryo	Cluster	0.667145
1%	Mouse_embryo	scMeformer	0.809612
1%	Mouse_embryo	Cluster	0.658802

Table S10. Precision estimates for DMRs identified from imputed data using various filtering criteria, Related to Figure 4.

Filter	total DMR number	overlapped with ground truth	precision
0.2	407,120	326,700	0.802
0.3	443,676	335,611	0.756
0.4	806,234	562,838	0.698
no filter	1,729,786	956,042	0.553
**Total number of ground truth DMRs**	**1,163,204**		

Table S13. Numbers of SCZ-associated DMRs detected in each cell type, stratified by direction of effect, Related to Figure 7

CellType	Direction	Count
MGEInh	All	3468
MGEInh	Up	2288
MGEInh	Down	1180
CGEInh	All	3868
CGEInh	Up	2415
CGEInh	Down	1453
DeepExc	All	5808
DeepExc	Up	4586
DeepExc	Down	1222
Glia	All	19265
Glia	Up	11993
Glia	Down	7272
SupExc	All	6055
SupExc	Up	3963
SupExc	Down	2092

Table S14. MAGMA gene set-based association with SCZ for genes linked to DMRs in each cell type, Related to Figure 7. Default parameters were used using a one-sided positive test, conditional on gene size, gene density, sample size, inverse MAC, log(gene size), log(gene density), log(sample size), and log(inverse MAC)

Gene sets	CellType	DMR Direction	NGENES	BETA	BETA_STD	SE	P	FDR
CGEInh	CGEInh	All	563	0.13803	0.024603	0.052707	0.004416	0.013248
CGEInh_up	CGEInh	Up	328	0.12664	0.017351	0.06822	0.031709	0.06645
CGEInh_down	CGEInh	Down	235	0.14616	0.016996	0.080666	0.035011	0.06645
MGEInh	MGEInh	All	514	0.097976	0.01671	0.054238	0.03544	0.06645
MGEInh_up	MGEInh	Up	277	0.11232	0.014163	0.074343	0.065428	0.098142
MGEInh_down	MGEInh	Down	237	0.079106	0.009238	0.078072	0.15548	0.1794
DeepExc	DeepExc	All	774	0.13711	0.028471	0.044408	0.001011	0.007583
DeepExc_up	DeepExc	Up	598	0.13714	0.025165	0.050595	0.003363	0.013248
DeepExc_down	DeepExc	Down	176	0.12089	0.012187	0.087101	0.082594	0.112628
SupExc	SupExc	All	783	0.13992	0.029214	0.044946	0.000928	0.007583
SupExc_up	SupExc	Up	477	0.15213	0.025023	0.057805	0.004252	0.013248
SupExc_down	SupExc	Down	306	0.11346	0.015025	0.069177	0.050494	0.084157
Glia	Glia	All	1181	0.028283	0.007164	0.036326	0.21812	0.2337
Glia_up	Glia	Up	601	0.051093	0.009398	0.049264	0.14985	0.1794
Glia_down	Glia	Down	580	0.00103	0.000186	0.051902	0.49209	0.49209

Table S15. Enrichment of genes linked to DMRs for differentially expressed genes identified in a single-cell RNA-seq study of SCZ, Related to Figure 7.

Cell type	DMR direction	enrichment fold	standard error	p
CGEInh	Up	1.369	0.288	1.73E-01
CGEInh	Down	0.557	1.008	8.343-1
MGEInh	Up	1.912	0.346	5.67E-02
MGEInh	Down	0.476	1.007	8.77E-01
DeepExc	Up	2.065	0.117	7.26E-09
DeepExc	Down	1.434	0.511	3.13E-01
SupExc	Up	1.546	0.164	7.05E-03
SupExc	Down	1.236	0.364	3.31E-01
Glia	Up	1.294	0.375	2.99E-01
Glia	Down	1.162	0.318	3.64E-01

Table S18. Estimated training time (hours) required for each model over 10 epochs across varying numbers of cells, Related to STAR Methods

Number of cells	CpG-Transformer	DeepCpG	GraphCpG	scMeFormer
4000	5560	2380	30820	72
2000	2780	530	17220	70
1000	440	290	5830	70
500	250	140	2610	69
200	80	80	970	70
100	50	60	510	68

Table S19. hyperparameters used in scMeFormer, Related to STAR Methods

**Hyperparameters**	**DNA module**	**CpG module**
Number of layers in CNN module	3	3
Number of kernels in each CNN layer	256	256
Kernel size for each CNN layer	10	11
Number of max-pooling layers	1	NA
Kernel size for max-pooling layer	20	NA
Step size for max-pooling layer	20	NA
Dropout rate for dropout layer	0.2	0.2
Number of attention layers in Transformer module	8	8
Number of attention headers	8	8
Input size	256	256
Hidden size	256	256
Intermediate size	1024	1024
Output size	256	256
Dropout rate	0.1	0.1
Number hidden layer in fully connected network	1	
Input size for fully connected network	768	
Hidden size for fully connected network	768	
Number of tasks (output size for fully connected	The number of cells	
Initial learning rate	0.000176	
Number of learning rate warmup steps	10000	
